# A rapid review of early guidance to prevent and control COVID-19 in custodial settings

**DOI:** 10.1186/s40352-021-00150-w

**Published:** 2021-10-15

**Authors:** Lindsay A. Pearce, Alaina Vaisey, Claire Keen, Lucas Calais-Ferreira, James A. Foulds, Jesse T. Young, Louise Southalan, Rohan Borschmann, Ruth Gray, Sunita Stürup-Toft, Stuart A. Kinner

**Affiliations:** 1 Justice Health Unit, Melbourne School of Population and Global Health, University of Melbourne, Level 4, 207 Bouverie Street, Carlton, Victoria 3053 Australia; 2 Centre for Adolescent Health, Murdoch Children’s Research Institute, Melbourne, Victoria Australia; 3 Mater Research Institute, University of Queensland, Brisbane, Queensland Australia; 4 Department of Psychological Medicine, University of Otago, Christchurch, New Zealand; 5 School of Population and Global Health, The University of Western Australia, Perth, Western Australia Australia; 6 National Drug Research Institute, Curtin University, Perth, Western Australia Australia; 7 Law School, University of Western Australia, Perth, Western Australia Australia; 8 Health Service and Population Research Department, Institute of Psychiatry, Psychology and Neuroscience, King’s College London, London, UK; 9 Melbourne School of Psychological Sciences, The University of Melbourne, Melbourne, Victoria Australia; 10 Healthcare in Prison, South Eastern Health and Social Care Trust, Belfast, North Ireland UK; 11 Global Public Health, Public Health England, London, England; 12 Griffith Criminology Institute, Griffith University, Brisbane, Queensland Australia; 13 School of Public Health and Preventive Medicine, Monash University, Melbourne, Victoria Australia

**Keywords:** COVID-19, Custodial settings, Prisons, Youth detention, Immigration detention, Forensic psychiatric facilities, Prevention and control, Guidance, Recommendations, Rapid review

## Abstract

**Background:**

With over 11 million people incarcerated globally, prevention and control of COVID-19 in custodial settings is a critical component of the public health response. Given the risk of rapid transmission in these settings, it is important to know what guidance existed for responding to COVID-19 in the early stages of the pandemic. We sought to identify, collate, and summarise guidance for the prevention and control of COVID-19 in custodial settings in the first six months of 2020. We conducted a systematic search of peer-reviewed and grey literature, and manually searched relevant websites to identify publications up to 30 June 2020 outlining recommendations to prevent and/or control COVID-19 in custodial settings. We inductively developed a coding framework and assessed recommendations using conventional content analysis.

**Results:**

We identified 201 eligible publications containing 374 unique recommendations across 19 domains including: preparedness; physical environments; case identification, screening, and management; communication; external access and visitation; psychological and emotional support; recreation, legal, and health service adaptation; decarceration; release and community reintegration; workforce logistics; surveillance and information sharing; independent monitoring; compensatory measures; lifting control measures; evaluation; and key populations/settings. We identified few conflicting recommendations.

**Conclusions:**

The breadth of recommendations identified in this review reflects the complexity of COVID-19 response in custodial settings. Despite the availability of comprehensive guidance early in the pandemic, important gaps remain in the implementation of recommended prevention and control measures globally, and in the availability of evidence assessing their effectiveness on reducing COVID-19 disease, impact on people in custody and staff, and implementation.

**Supplementary Information:**

The online version contains supplementary material available at 10.1186/s40352-021-00150-w.

## Background

With over 11 million people incarcerated on any given day and an estimated 30 million people released from custody each year globally (Penal Reform International, 2020b; United Nations Office on Drugs and Crime, 2013), the prevention and control of COVID-19 in custodial settings is a critical component of the public health response. However, custodial settings – including prisons, jails and police cells, youth detention, immigration detention, and forensic psychiatric facilities – present a multitude of challenges for the prevention and control of COVID-19. These settings are often characterised by overcrowding, poor ventilation, inadequate access to sanitation, and substandard access to, and quality of, healthcare relative to the community (Dolan et al., 2016; Penal Reform International, 2020b). Furthermore, infectious diseases can be easily transmitted between people in custody, staff, and visitors through facility transfers and staff cross-deployment, and to and from the community via intakes and releases. These conditions make custodial settings high-risk environments for COVID-19 transmission, and subsequent community spread (Beaudry et al., 2020; Penal Reform International, 2020b).

Custodial settings concentrate marginalised populations with disproportionately high rates of mental illness (Fazel et al., 2016), substance dependence (Fazel et al., 2006), communicable (Dolan et al., 2016) and non-communicable disease (Herbert et al., 2012), intellectual disability (Fazel et al., 2008), and multimorbidity (Kinner & Young, 2018; Penal Reform International, 2020b; World Health Organization Europe, 2014). People in custody are therefore more likely than people in the general population to be susceptible to severe COVID-19 disease (World Health Organization and the United Nations Development Programme, 2020). However, they are also highly vulnerable to the deleterious physical and mental health impacts of intensified and sustained confinement, which are common to COVID-19 prevention and control measures. Strategies that restrict freedoms and meaningful social interaction, such as facility lockdown and isolation, increase psychological distress and adverse outcomes for people in custody, with particularly profound impacts on those with pre-existing mental illness (Hewson et al., 2020; Stewart et al., 2020). These are important considerations for COVID-19 response in custodial settings, which must balance obligations to protect the health and human rights of people in custody (United Nations Human Rights Office of the High Commissioner, 1985; United Nations Office on Drugs and Crime, 2011; United Nations Office on Drugs and Crime, 2015) with the need to minimise COVID-19 morbidity and mortality.

Recognising these vulnerabilities and their implications for public health, the COVID-19 pandemic prompted a rapid influx of published guidance to prevent the introduction and transmission of COVID-19 in custodial settings. Based on this guidance, governments and correctional authorities were tasked to quickly mount a response to COVID-19 within justice and immigration detention systems that, in many settings, historically operated in isolation from community and public health sectors. To date, there has been no systematic effort to collate and summarise these initial recommendations and to assess the extent to which clear guidance was available in the crucial early stages of the pandemic. This is important to identify areas requiring clarification or additional guidance, and to inform evaluation and research. We therefore aimed to identify, collate, and summarise guidance for the prevention and control of COVID-19 in custodial settings in the first six months of 2020.

## Methods

### Overview

We conducted a rapid review of peer-reviewed and grey literature to identify guidance for COVID-19 prevention and control in custodial settings. We defined custodial settings as inclusive of prisons, jails and police cells, youth detention settings, immigration detention settings, and forensic psychiatric facilities. We registered a protocol with the International Prospective Register of Systematic Reviews (PROSPERO; CRD42020191735).

### Information sources

We searched the following databases: Medline (Ovid), PsycINFO, Embase, Web of Science, CINAHL, Global Health (CABI), Criminal Justice Abstracts, LILACS, LitCovid, and Google Scholar. We searched an additional three databases indexing grey literature: WorldWideScience, TRIP Database, and Google Search Engine. The first 20 pages of Google results were screened to identify relevant publications. We manually searched COVID-19 ‘information hubs’ that indexed resources relevant to custodial settings, websites of organisations with an interest in the health or human rights of people in custody, and reference lists.

### Search strategy

Our search strategy is detailed in Appendix S3. We used variants and combinations of search terms relating to two key concepts: COVID-19, and custodial settings. We used all common variants of COVID-19, based on a preliminary literature search. We included terms related to the criminal justice system, deprivation of liberty, and specific types of custodial settings. Terms relating to ‘recommendations’ or ‘guidance’ were used to focus Google results. All peer-reviewed database searches were conducted on 1 July 2020, capturing publications up to and including 30 June 2020. Grey literature data sources were searched between 1 July 2020 and 10 July 2020.

### Eligibility criteria

Eligible publications had to outline recommendations for the prevention and/or control of COVID-19 in custodial settings and be published in English. To ensure a feasible number of grey literature publications, we limited eligibility to those published by federal and state/provincial/territorial governments, international organisations (e.g., United Nations), specialised intergovernmental agencies (e.g., World Health Organization [WHO]), and organisations with an interest in the health and/or human rights of people in custody. We also excluded from grey literature news articles and blog posts; opinion pieces and commentaries not published in peer-reviewed journals (to act as a quality control via the peer-review process); publications focusing on policing measures; and legal documents including proceedings, summaries, and case reports.

### Selection process

**Fig. 1:**
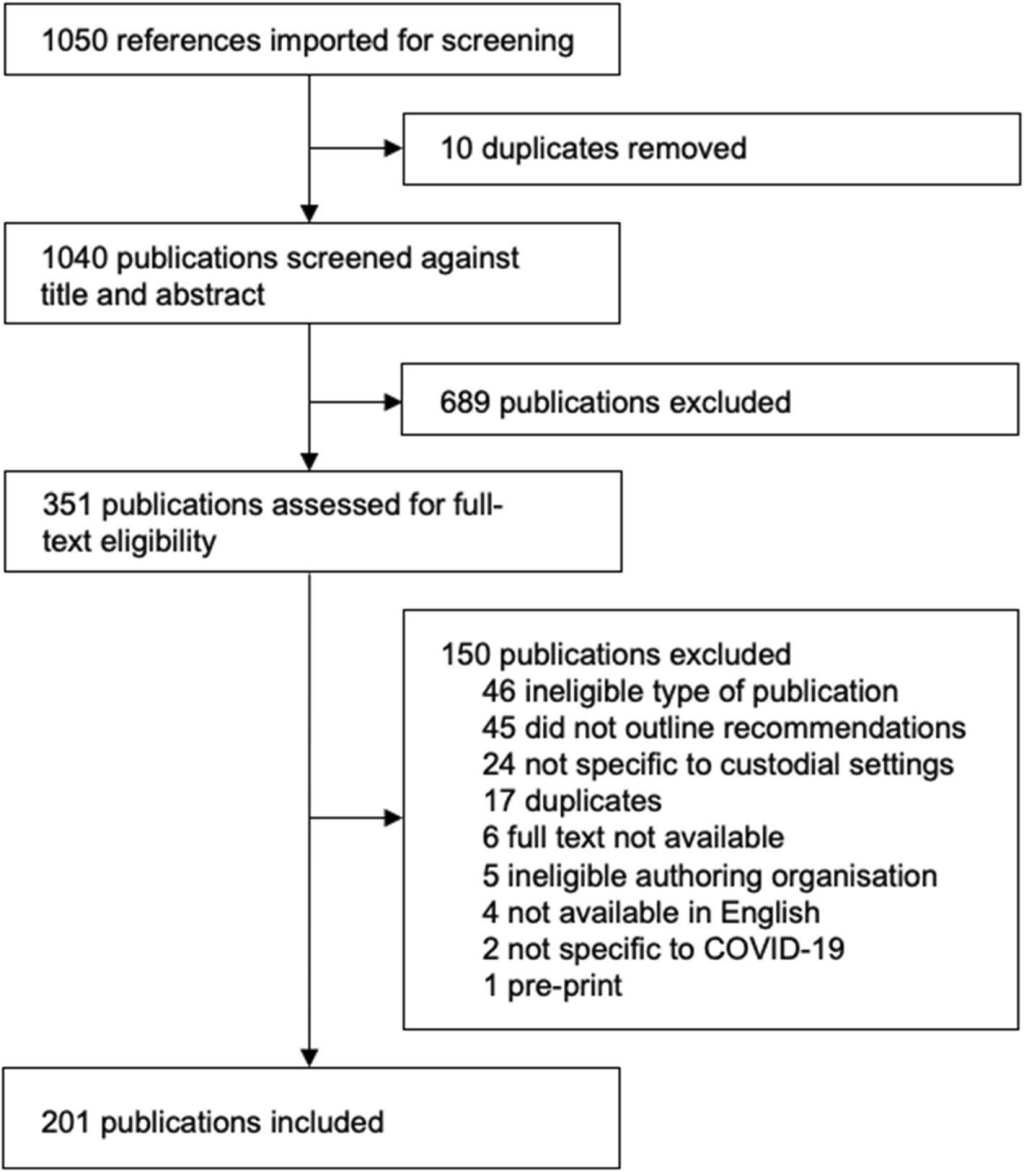
PRISMA flowchart

Initial eligibility of grey literature identified from Google and manual searches was decided by one reviewer (LP or JF) at the time of the search, by applying a priori eligibility criteria. References were uploaded to Covidence systematic review management software (Covidence, 2021) and duplicates removed. Title and abstract screening were conducted by two independent reviewers (CK, LP). If an abstract or summary was not available, the reviewer conducted a brief full-text screening to assess eligibility. Disagreements in eligibility were resolved through discussion with a third reviewer (LCF). Full-text review was conducted by two independent reviewers (CK, LP, AV) and disagreements in eligibility were resolved through consensus with a third reviewer. The selection process is illustrated in Fig. 1.

### Quality assessment

No formal quality assessment was applied due to our focus on extracting recommendations rather than assessing results.

### Analysis

We developed a data extraction form to collate information on all included publications (Appendix S2). We conducted a conventional content analysis to systematically examine text and develop domains within which to categorise recommendations (Hall & Steiner, 2020; Hsieh & Shannon, 2005). To inductively develop an initial coding framework, we purposively sampled 14 publications that comprehensively provided recommendations across key custodial settings and population subgroups. Three authors (CK, LP, AV) independently reviewed and coded each publication. Independent codes were combined and grouped into domains to develop a preliminary coding framework, which was reviewed and refined by co-authors. An additional 17 publications were coded against the interim framework to identify gaps and facilitate further refinement into a final coding framework (Table 2). Each remaining publication was coded by one author (CK, LP, AV) and any uncertainties were resolved by consensus with a second author. Recommendations were summarised by domain and sub-domain. We did not code detailed guidance for the clinical management of COVID-19 cases, testing, and contact tracing, unless recommendations were specific to custodial settings. We used NVivo 12 (QSR International, 2021) for coding and document storage.

## Results

We identified 201 eligible publications (Appendix S2). Of these, 142 (71%) were grey literature (67 general guidance, 44 statements, 16 reports, 6 press releases, 4 clinical guidance, 2 official plans, 3 policy briefs) and 59 (29%) were peer-reviewed (37 commentaries, 8 research articles, 7 letters to the editor, 7 opinion editorials). Eighty-six (43%) contained specific recommendations for adult prisons; 26 (13%) for youth detention; 31 (15%) for immigration detention; 5 (2%) for forensic psychiatric facilities; 9 (5%) for community-based detention, probation, or parole; 7 (3%) for legal proceedings; and 70 (35%) for deprivation of liberty more generally. Sixty-eight (34%) were not specific to any WHO-defined region (i.e., published by an international organisation), 67 (33%) were from the Americas, 35 (17%) were from Europe, 17 (8%) were from the Western Pacific, 6 (3%) were from the Eastern Mediterranean, 4 (2%) were from Africa, and 4 (2%) were from South-East Asia.

We identified 12 high-level, guiding principles (Table 1) and grouped the remaining 374 unique recommendations into 19 domains (Table 2). A brief summary of recommendations from each domain is presented below; a full list is provided in Appendix S1.

**Table 1:** Summary of guiding principles for the prevention and control of COVID-19 in custodial settings

Summary of guiding principles for the prevention and control of COVID-19 in custodial settings	
Correctional authorities and governments have a responsibility for, and must protect, the health and safety of people deprived of liberty and staff (American Academy of Pediatrics, 2020; Amnesty International, 2020a; Amnesty International and Justice Project Pakistan, 2020; Commonwealth Human Rights Initiative, 2020; Council of Europe Commissioner for Human Rights, 2020b; European Centre for Disease Prevention and Control, 2020a; European Committee for the Prevention of Torture and Inhuman or Degrading Treatment or Punishment (CPT), 2020; International Committee of the Red Cross (ICRC), 2020; International Corrections and Prisons Association (ICPA), 2020; International Detention Coalition, 2020; International Federation for Human Rights, 2020a; Lachsz & Hurley, 2020; Penal Reform International, 2020a; Sanchez et al., 2020; The Alliance for Child Protection in Humanitarian Action, 2020; UNICEF, 2020; United Nations Human Rights Office of the High Commissioner, 2020a, 2020c; United Nations Office on Drugs and Crime, 2020c, 2020e; World Health Organization, 2020c)	
Correctional authorities must uphold internationally recognised human rights standards (United Nations Human Rights Office of the High Commissioner, 1985; United Nations Office on Drugs and Crime, 2011; United Nations OFfice on Drugs and Crime (UNODC), 2015) to maintain a safe and humane custodial environment (Amnesty International and Justice Project Pakistan, 2020; Australia New Zealand Scholars, 2020; Australian Scholars, 2020; Barnert et al., 2020; Commonwealth Human Rights Initiative, 2020; Council of Europe Commissioner for Human Rights, 2020b; Crowley et al., 2020; European Centre for Disease Prevention and Control, 2020b; European Committee for the Prevention of Torture and Inhuman or Degrading Treatment or Punishment (CPT), 2020; Inter-Agency Standing Committee, 2020; International Federation for Human Rights, 2020b; Lachsz & Hurley, 2020; National Aboriginal & Torres Strait Islander Legal Services, 2020a; Sanchez et al., 2020; Special Rapporteur on Extrajudicial Summary or Arbitrary Killings, 2020; The Alliance for Child Protection in Humanitarian Action, 2020; UNICEF, 2020; United Nations Human Rights Office of the High Commissioner, 2020a, 2020b, 2020d, 2020f; United Nations Office on Drugs and Crime, 2020a, 2020c, 2020e; Waly et al., 2020; World Health Organization, 2020a, 2020c)	
COVID-19 prevention and control measures must be proportionate to the health risk, necessary, time-limited, non-discriminatory, legal, transparent, and the least intrusive option (AMEND, 2020a; Amnesty International, 2020a, 2020b; Association for the Prevention of Torture, 2020; Commonwealth Human Rights Initiative, 2020; Council of Europe, 2020; Council of Europe Commissioner for Human Rights, 2020b; Danish Institute Against Torture, 2020a, 2020b; European Committee for the Prevention of Torture and Inhuman or Degrading Treatment or Punishment (CPT), 2020; Human Rights Watch, 2020; Inter-Agency Standing Committee, 2020; International Committee of the Red Cross (ICRC), 2020; International Corrections and Prisons Association (ICPA), 2020; International Detention Coalition, 2020; International Federation for Human Rights, 2020a; New Zealand Office of the Ombudsman, 2020; Tahrir Institute for Middle East Policy (TIMEP) and Middle East and North Africa (MENA) Rights Group, 2020; The Alliance for Child Protection in Humanitarian Action, 2020; United Nations, 2020; United Nations Human Rights Office of the High Commissioner, 2020a, 2020b, 2020c, 2020d; United Nations Office on Drugs and Crime, 2020b; Waly et al., 2020; World Health Organization, 2020c; World Organization Against Torture, 2020a, 2020b, 2020c)	
People in custody must receive regular, timely, consistent, and transparent information on COVID-19 risk reduction, active outbreaks, and prevention and control measures implemented; communication strategies should be tailored to meet diverse physical, cultural, literary, and cognitive needs (AMEND, 2020a; Barnert et al., 2020; Communicable Diseases Network Australia, 2020; European Centre for Disease Prevention and Control, 2020a; European Committee for the Prevention of Torture and Inhuman or Degrading Treatment or Punishment (CPT), 2020; Hewson et al., 2020; Independent Advisory Panel on Deaths in Custody, 2020; Innovative Prison Systems, 2020; Inter-Agency Standing Committee, 2020; International Committee of the Red Cross (ICRC), 2020; Justice and Corrections Service, 2020; New Zealand Office of the Ombudsman, 2020; United Nations Human Rights Office of the High Commissioner, 2020a, 2020b, 2020d; US Centers for Disease Control and Prevention, 2020a; World Health Organization, 2020a; World Organization Against Torture, 2020a; Wurcel et al., 2020)	
People in custody must receive an equivalent standard of health care to that available in the community, including when it pertains to COVID-19 prevention, testing, and treatment (AMEND, 2020a; Amnesty International, 2020b; Amnesty International and Justice Project Pakistan, 2020; Association for the Prevention of Torture, 2020; Australian Scholars, 2020; Council of Europe, 2020; Council of Europe Commissioner for Human Rights, 2020b; Crowley et al., 2020; Danish institute Against Torture, 2020b; European Centre for Disease Prevention and Control, 2020a; Human Rights Watch, 2020; Inter-Agency Standing Committee, 2020; International Committee of the Red Cross (ICRC), 2020; New Zealand Office of the Ombudsman, 2020; Royal College of General Practitioners Secure Environments Group, 2020; Special Rapporteur on Extrajudicial Summary or Arbitrary Killings, 2020; Tahrir Institute for Middle East Policy (TIMEP) and Middle East and North Africa (MENA) Rights Group, 2020; The Alliance for Child Protection in Humanitarian Action, 2020; UNAIDS, 2020; United Nations, 2020; United Nations Human Rights Office of the High Commissioner, 2020a, 2020e; United Nations Network on Migration, 2020; United Nations Office on Drugs and Crime, 2020a; Waly et al., 2020; World Health Organization, 2020a, 2020c; World Organization Against Torture, 2020a)	
People in custody must maintain the right to legal representation and continued deprivation of liberty must consider the current conditions of detention, particularly within the context of compulsory medical isolation and other measures that introduce additional restrictions on personal freedom (Association for the Prevention of Torture, 2020; Council of Europe Commissioner for Human Rights, 2020a; Danish Institute Against Torture, 2020a; FIACAT, 2020; Inter-Agency Standing Committee, 2020; International Corrections and Prisons Association (ICPA), 2020; International Detention Coalition, 2020; National Juvenile Defender Center, 2020; Tahrir Institute for Middle East Policy (TIMEP) and Middle East and North Africa (MENA) Rights Group, 2020; Terres des hommes, 2020; The International Legal Foundation, 2020; United Nations Human Rights Office of the High Commissioner, 2020c, 2020e; United Nations Women, 2020)	
Compensatory measures that alleviate the potentially harmful impacts of restrictive COVID-19 prevention and control measures on the physical, emotional, and mental health of people in custody should be applied (Association for the Prevention of Torture, 2020; Australia New Zealand Scholars, 2020; Council of Europe, 2020; Hewson et al., 2020; Stewart et al., 2020; Tahrir Institute for Middle East Policy (TIMEP) and Middle East and North Africa (MENA) Rights Group, 2020)	
Independent monitoring and oversight, whether conducted in-person or remotely, must continue to monitor respect for fundamental rights (Association for the Prevention of Torture, 2020; Australian Scholars, 2020; Avocats Sans Frontières, 2020; Commonwealth Human Rights Initiative, 2020; Council of Europe Commissioner for Human Rights, 2020b; Danish institute Against Torture, 2020b; European Committee for the Prevention of Torture and Inhuman or Degrading Treatment or Punishment (CPT), 2020; Inspectorate of Prisons for Scotland, 2020; Inter-Agency Standing Committee, 2020; International Corrections and Prisons Association (ICPA), 2020; Lachsz & Hurley, 2020; New South Wales Government, 2020; Penal Reform International, 2020a; The Alliance for Child Protection in Humanitarian Action, 2020; United Nations Human Rights Office of the High Commissioner, 2020a, 2020b, 2020c; World Health Organization, 2020b; World Organization Against Torture, 2020a)	
Immediate action to reduce prison population density is needed to address widespread overcrowding in correctional settings, and must be applied with adequate transition planning to facilitate safe reintegration into the community (Alohan & Calvo, 2020; American Academy of Pediatrics, 2020; Amnesty International, 2020a; Annie E. Casey Foundation, 2020; Council of Europe Commissioner for Human Rights, 2020a, 2020b; Henry, 2020; Piel, 2020; Rubenstein, 2020; Simpson & Butler, 2020; Sivashanker et al., 2020; Terres des hommes, 2020; The Alliance for Child Protection in Humanitarian Action, 2020; UNICEF, 2020; World Organization Against Torture, 2020c)	
A sustainable response to the COVID-19 pandemic requires carceral system reform rooted in health equity to address ongoing crises of overcrowding, poor living conditions, and substandard health care in custodial settings (Alohan & Calvo, 2020; Crowley et al., 2020; Minkler et al., 2020; The Alliance for Child Protection in Humanitarian Action, 2020)	
COVID-19 prevention and control measures should be tailored to the local and cultural context, resource availability, and specific needs of key vulnerable populations (Council of Europe Commissioner for Human Rights, 2020b; European Committee for the Prevention of Torture and Inhuman or Degrading Treatment or Punishment (CPT), 2020; International Federation for Human Rights, 2020b; The Alliance for Child Protection in Humanitarian Action, 2020; United Nations, 2020; United Nations Office on Drugs and Crime, 2020e; United Nations Women, 2020; World Health Organization, 2020c; World Organization Against Torture, 2020a)	
Close collaboration between health and justice sectors is essential for an effective, coordinated, whole-of-government response (Akiyama et al., 2020; AMEND, 2020a; Amnesty International, 2020a; Association for the Prevention of Torture, 2020; Australian Scholars, 2020; Barnert et al., 2020; Centers for Disease Control and Prevention, 2020; Communicable Diseases Network Australia, 2020; Danish institute Against Torture, 2020b; Gorman & Ramaswamy, 2020; Hewson et al., 2020; Human Rights Watch, 2020; Innovative Prison Systems, 2020; Inter-Agency Standing Committee, 2020; Kinner et al., 2020; Liebrenz et al., 2020; Montoya-Barthelemy et al., 2020; National Commission on Correctional Health Care, 2020b; Penal Reform International, 2020a; Simpson & Butler, 2020; The Alliance for Child Protection in Humanitarian Action, 2020; United Nations Human Rights Office of the High Commissioner, 2020d; United Nations Office on Drugs and Crime, 2020d, 2020f; US Centers for Disease Control and Prevention, 2020b; Wallace et al., 2020; World Health Organization, 2020c; Yang & Thompson, 2020)

**Table 2:** Key domains of COVID-19 response derived from recommendations

#	Domain	Sub-domain
1	Planning and preparedness	Facility-level Regional
2	Creating safer physical environments	Personal and hand hygiene Cleaning and sanitation Physical distancing Cohorting Day-to-day personal protective equipment (PPE) Ventilation Non-medical transfers
3	Case identification and screening	
4	Case management	Clinical management Medical isolation PPE Medical referral and transfer Contact tracing
5	Communicating to people in custody, staff, and families	
6	External access and visitation	In-person access and visitation Remote access and visitation
7	Psychological and emotional support	Support for people in custody Support for staff Support for families
8	Adapting healthcare provision	
9	Adapting recreation, programming, and services	
10	Adapting legal services and processes	Hearings and court proceedings Access to legal representation Bail, remand, probation, parole and community supervision
11	Decarceration	Reducing justice or immigration system involvement Releasing people in custody
12	Release and community reintegration	Pre-release needs assessment Post-release support
13	Workforce logistics	Staff briefings and training Staffing policies and protocols Managing staff as confirmed cases or close contacts
14	Surveillance and information sharing	
15	Independent monitoring and inspection	
16	Compensatory measures	
17	Lifting control measures	
18	Learning systems and evaluative frameworks	
19	Key populations and settings*	Youth detention Immigration detention Forensic psychiatric Low-middle income countries (LMIC) Women Elderly Indigenous peoples People with a disability People who use alcohol and other drugs (AOD) People with mental illness Other key populations

* Key populations were identified in the data when a specific recommendation was made for a population subgroup, and therefore do not represent all populations of people in custody

### Domain 1: planning and preparedness

Early intervention was recommended to quickly identify and respond to COVID-19 outbreaks. Guidelines and checklists for facility-level planning and preparedness (Justice and Corrections Service, 2020; World Health Organization, 2020a) were developed and recommended. Outbreak management plans that identify steps for rapid case identification, isolation, and treatment; communication plans that facilitate rapid decision making; and contingency plans for staff shortages were recommended. This included collaboration with other custodial facilities for joint surveillance and between-facility transfers (Communicable Diseases Network Australia, 2020; US Centres for Disease Control and Prevention, 2020a; US Centres for Disease Control and Prevention, 2020b; US Centres for Disease Control and Prevention, 2020c), and with local health services to support regional contact tracing and medical case management (Communicable Diseases Network Australia, 2020; Kinner et al., 2020; Lachsz & Hurley, 2020; Penal Reform International, 2020a; Wang et al., 2020; Yang & Thompson, 2020).

### Domain 2: creating safer physical environments

‘Cohorting’, the practice of restricting movement between groups of people in custody (e.g., housing units, close contacts and confirmed cases, or medically vulnerable people from the remainder of the custodial population), was recommended to increase biosecurity (AMEND, 2020b; Association for the Prevention of Torture, 2020; Commonwealth Human Rights Initiative, 2020; Communicable Diseases Network Australia, 2020; European Centre for Disease Prevention and Control, 2020b; Public Health England, 2020a; Public Health England, 2020b; Royal College of General Practitioners Secure Environments Group, 2020; US Centres for Disease Control and Prevention, 2020a; US Centres for Disease Control and Prevention, 2020b; US Centres for Disease Control and Prevention, 2020c). It was recommended that cohorted groups and their allocated staff remain together for all daily activities and maintain physical distance from other cohorts (AMEND, 2020b; Communicable Diseases Network Australia, 2020; National Commission on Correctional Health Care, 2020a; National Commission on Correctional Health Care, 2020b; Public Health England, 2020b; United Nations Institute for Training and Research (UNITAR), 2020). Free access to personal hygiene supplies (e.g., soap, hand sanitiser, clean towels) was widely recommended (Centers for Disease Control and Prevention, 2020; European Committee for the Prevention of Torture and Inhuman or Degrading Treatment or Punishment (CPT), 2020; Seal, 2020; U.S. Immigration and Customs Enforcement, 2020; US Centres for Disease Control and Prevention, 2020a). A comprehensive list of recommendations to create safer physical environments is provided in Appendix S1.

### Domain 3: case identification and screening

Universally accessible, free, and equitable COVID-19 testing of symptomatic and asymptomatic people in custody and staff was recommended for early detection and management of COVID-19 (The Kirby Institute, 2020). It was recommended that all admissions and visitors entering the facility be screened and admissions be tested for COVID-19 (Centers for Disease Control and Prevention, 2020; Commonwealth Human Rights Initiative, 2020; General Directorate “Execution of Sentences” Bulgaria, 2020; Inter-Agency Standing Committee, 2020; Meyer et al., 2020; United Nations Office on Drugs and Crime, 2020g; US Centres for Disease Control and Prevention, 2020a; US Centres for Disease Control and Prevention, 2020b; US Centres for Disease Control and Prevention, 2020c; Wang et al., 2020). Recommendations regarding routine quarantine of all intakes were mixed; we identified five publications recommending routine quarantine at intake (Centers for Disease Control and Prevention, 2020; U.S. Immigration and Customs Enforcement, 2020; Njuguna et al., 2020; O’Moore & Farrar, 2020; US Centres for Disease Control and Prevention, 2020b), whereas two publications discouraged its use due to mental health implications and preference towards comprehensive testing and screening (Lachsz & Hurley, 2020; World Health Organization, 2020b). If testing capacity is limited, it was recommended to prioritise people at high risk of complications from COVID-19, people at high risk of transmitting COVID-19, and symptomatic individuals (AMEND, 2020a; International Federation for Human Rights, 2020).

### Domain 4: case management

It was recommended that all suspected and confirmed cases of COVID-19 have immediate access to healthcare and be safely transferred to community health services when required (AMEND, 2020c; Communicable Diseases Network Australia, 2020; Inter-Agency Standing Committee, 2020; New Zealand Office of the Ombudsman, 2020; Penal Reform International, 2020a; Royal College of Psychiatrists, 2020; UNAIDS, 2020). Use of medical isolation was recommended only to protect the health of individuals and people around them, with distinct conditions from punitive solitary confinement (AMEND, 2020c; Council of Europe, 2020; Inter-Agency Standing Committee, 2020; US Centres for Disease Control and Prevention, 2020a) including access to additional psychological support and meaningful daily human contact (AMEND, 2020c; Association for the Prevention of Torture, 2020; Australian Scholars, 2020; European Committee for the Prevention of Torture and Inhuman or Degrading Treatment or Punishment (CPT), 2020; Innovative Prison Systems, 2020; International Corrections and Prisons Association (ICPA), 2020; Penal Reform International, 2020a) (see ‘Remote access & visitation’ in Appendix S1). Other measures that protect the emotional and social wellbeing of people in medical isolation, including prevention of violence and discrimination towards suspected or confirmed cases (Lachsz & Hurley, 2020), were recommended to convey the non-punitive nature of treatment, encourage symptom reporting and early healthcare intervention, and improve health and mortality outcomes (AMEND, 2020c; Barnert et al., 2020; Penal Reform International, 2020a).

### Domain 5: communicating to people in custody, staff, and families

Consistent, timely, transparent, and accessible information sharing with people in custody, staff, families, and the public was recommended to reduce fear and anxiety, establish and maintain trust, maximise compliance with preventive measures, promote access to medical care, reduce unrest, and hold custodial authorities accountable to the health and human rights of people in custody (AMEND, 2020b; Commonwealth Human Rights Initiative, 2020; European Centre for Disease Prevention and Control, 2020a; Hewson et al., 2020; International Corrections and Prisons Association (ICPA), 2020; Pyrooz et al., 2020; United Nations Office on Drugs and Crime, 2020e). This included clear explanations of COVID-19 symptoms and prevention, new restrictions and their impact on daily routine, and containment procedures for confirmed cases. Several recommendations highlighted that communications must meet diverse cognitive, disability, health literacy, and language needs (Barnert et al., 2020; European Centre for Disease Prevention and Control, 2020a; International Corrections and Prisons Association (ICPA), 2020; United Nations Office on Drugs and Crime, 2020d; US Centres for Disease Control and Prevention, 2020a; World Health Organization, 2020a).

### Domain 6: external access and visitation

Recommendations for external access and visitation ranged from allowing some in-person visitation with protective measures in place (Innovative Prison Systems, 2020; Justice and Corrections Service, 2020; US Centres for Disease Control and Prevention, 2020a; US Centres for Disease Control and Prevention, 2020b; US Centres for Disease Control and Prevention, 2020c; World Organization Against Torture, 2020a) to restricting all non-essential vendors, volunteers, and visitors from entering facilities (AMEND, 2020b; Innovative Prison Systems, 2020; Penal Reform International, 2020a; Prison department of the Republic of Lithuania, 2020; Seal, 2020). It was recommended that restrictions on in-person visitation be offset by temporary reductions or elimination of costs for telephone calls, videoconferencing, and e-mail (American Academy of Pediatrics, 2020; Commonwealth Human Rights Initiative, 2020; Council of Europe, 2020; U.S. Immigration and Customs Enforcement, 2020; Lachsz & Hurley, 2020; National Aboriginal & Torres Strait Islander Legal Services, 2020a; New Zealand Office of the Ombudsman, 2020; Royal College of Psychiatrists, 2020; The Alliance for Child Protection in Humanitarian Action, 2020; United Nations Human Rights Office of the High Commissioner, 2020a; United Nations Human Rights Office of the High Commissioner, 2020c; United Nations Office on Drugs and Crime, 2020d; US Centres for Disease Control and Prevention, 2020a; US Centres for Disease Control and Prevention, 2020b; US Centres for Disease Control and Prevention, 2020c; Vera Institute of Justice, 2020a; Vera Institute of Justice, 2020b; World Organization Against Torture, 2020a; Youth Correctional Leaders for Justice, 2020). It was recommended that decisions to restrict in-person visitation recognise the diverse roles of visitors, including the provision of money, food, and other essential supplies; two publications recommended that custodial authorities adapt protocols so that these resources continue to safely reach people in custody (Amnesty International, 2020a; Amnesty International, 2020b; Association for the Prevention of Torture, 2020).

### Domain 7: psychological and emotional support

The protection of the mental and emotional health of people in custody and staff was a recurrent theme. It was recommended that additional psychological support and compensatory measures be made available during periods of sustained restriction or isolation (*see Domain 16 ‘Compensatory measures’*). Several publications (Knox, 2020; Kothari et al., 2020; Wang et al., 2020; World Health Organization, 2020b; World Organization Against Torture, 2020a) recommended that custodial settings develop capacity to monitor stress, burnout, and fatigue among staff, and/or counteract these through enhanced, no-cost psychological support programs and opportunities for debriefing with colleagues.

### Domain 8: adapting healthcare provision

Several recommendations reinforced human rights standards (United Nations Office on Drugs and Crime, 2015) that hold custodial authorities accountable for the provision of adequate medical care for persons in their custody. It was recommended that healthcare services adapt to respond to COVID-19 whilst ensuring that the broader healthcare needs of people in custody were not unjustifiably compromised (European Centre for Disease Prevention and Control, 2020a; International Corrections and Prisons Association (ICPA), 2020; United Nations Office on Drugs and Crime, 2020b; United Nations Office on Drugs and Crime, 2020c). Free access to healthcare, at a minimum for respiratory symptoms, was recommended to facilitate early detection and treatment of COVID-19 (Mukherjee & El-Bassel, 2020; Rubenstein, 2020; US Centres for Disease Control and Prevention, 2020a; US Centres for Disease Control and Prevention, 2020b; US Centres for Disease Control and Prevention, 2020c; Wagner & Widra, 2020). In publications addressing healthcare provision, telemedicine was widely recommended (European Centre for Disease Prevention and Control, 2020a; HM Prison & Probation Service, 2020; Innovative Prison Systems, 2020; Royal College of General Practitioners Secure Environments Group, 2020; Royal College of Psychiatrists, 2020). Several publications recommended seasonal influenza vaccinations for all people in custody and staff to discount seasonal flu to the greatest extent possible from assessment of suspected COVID-19 cases and to reduce demand for healthcare services (Communicable Diseases Network Australia, 2020; U.S. Immigration and Customs Enforcement, 2020; Mukherjee & El-Bassel, 2020; Sanchez et al., 2020).

### Domain 9: adapting recreation, programming, and services

Educational, vocational, social, and religious programs can help to prepare people in custody for successful integration into the community (León et al., 2020) and reduce recidivism (Pyrooz et al., 2020). In circumstances where these programs cannot be adapted to meet infection prevention and control standards, it was recommended that steps be taken to compensate for suspended vocational programs and provide electronic entertainment and social activities, online education, and virtual religious services (Council of Europe, 2020; Innovative Prison Systems, 2020; León et al., 2020). In accordance with human rights standards (United Nations Human Rights Office of the High Commissioner, 1985; United Nations Office on Drugs and Crime, 2011; United Nations Office on Drugs and Crime, 2015), it was asserted that outdoor access not fall below a minimum of 1 hour per day (United Nations Office on Drugs and Crime (UNODC), 2020b; World Health Organization, 2020b).

### Domain 10: adapting legal services and processes

Continued functioning of courts and access to legal services, including the establishment of emergency courts (Inter-Agency Standing Committee, 2020), was recommended to support decarceration (Association for the Prevention of Torture, 2020; Tahrir Institute for Middle East Policy (TIMEP) and Middle East and North Africa (MENA) Rights Group, 2020; The Alliance for Child Protection in Humanitarian Action, 2020; The International Legal Foundation, 2020; United Nations, 2020) by reducing numbers of unsentenced people held in pre-trial detention - currently over 3 million people globally (Penal Reform International, 2020b). In contrast, one publication recommended the temporary suspension of judicial hearings to reduce transmission, with exception of remote hearings for urgent cases (Innovative Prison Systems, 2020). Virtual court hearings were recommended with careful consideration of due process, data security, and the vulnerabilities of children and people with a disability (United Nations Office on Drugs and Crime, 2020a). An important consideration was that adaptations to legal proceedings must not compromise the right to a fair trial and the safety of defendants, witnesses, and victims (United Nations Office on Drugs and Crime, 2020a).

### Domain 11: Decarceration

Decarceration strategies (Henry, 2020) were widely recommended to reduce prison overcrowding (Table 1). One common recommendation was that continued detention must be justified as necessary and proportionate within the context of COVID-19, particularly for those at high risk of harm from COVID-19 infection and/or restrictive prevention and control measures (United Nations Human Rights Office of the High Commissioner, 2020b; United Nations Human Rights Office of the High Commissioner, 2020c). Broad reviews of criminal justice and immigration policies were recommended to address an over-reliance on incarceration that disproportionately impacts medically vulnerable and marginalised populations (Danish Institute Against Torture, 2020a; Danish Institute Against Torture, 2020b; Mukherjee & El-Bassel, 2020; Nowotny et al., 2020; The Alliance for Child Protection in Humanitarian Action, 2020; UNAIDS, 2020; Wurcel et al., 2020). Recommended non-custodial measures to reduce detention at pre-trial, sentencing, and post-trial are detailed in Appendix S1.

### Domain 12: release and community re-integration

Post-release transitional support was recommended to ensure that people leaving custody during the pandemic are able to access health, social, and housing services that allow them to comply with local public health advice (Montoya-Barthelemy et al., 2020; Mukherjee & El-Bassel, 2020; United Nations Network on Migration, 2020). Pre-release needs assessment and transitional planning were recommended for all people leaving custody, particularly for those at highest risk of harm post-release, including people with substance use disorders, mental illness, chronic illness, and housing instability (Gorman & Ramaswamy, 2020). It was recommended that custodial authorities carefully weigh the benefits of early release with capacity for transitional planning and support (Piel, 2020; Shepherd & Spivak, 2020).

### Domain 13: workforce logistics

Publications emphasised the protection of staff safety and wellbeing (Openshaw & Travassos, 2020). Consistent and transparent communication regarding policy changes, revised duties, and responsibilities during COVID-19 outbreaks were recommended (Emory Center for the Health of Incarcerated Persons, 2020; European Centre for Disease Prevention and Control, 2020b; Justice and Corrections Service, 2020). It was recommended that management plans and policies (e.g., paid sick leave) be in place to prepare for workforce disruptions, reduce unnecessary staff contact, protect high-risk staff members, and support quarantine and isolation when required (US Centres for Disease Control and Prevention, 2020a; US Centres for Disease Control and Prevention, 2020b; US Centres for Disease Control and Prevention, 2020c; World Health Organization, 2020a).

### Domain 14: surveillance and information sharing

Several publications recommended regular and transparent surveillance and information sharing with the public and local health authorities to inform local COVID-19 responses and hold authorities accountable for the fair treatment of people in custody (Communicable Diseases Network Australia, 2020; European Centre for Disease Prevention and Control, 2020b; Government of Canada Office of the Correctional Officer, 2020; Lachsz & Hurley, 2020). This included information on COVID-19 testing, case numbers, deaths, incidents of harm or unrest, outbreak management plans, and other contingency plans. One publication recommended that regions unable to immediately implement surveillance consider a phased approach involving voluntary reporting or sentinel surveillance that can serve as indicators for the wider region (European Centre for Disease Prevention and Control, 2020b).

### Domain 15: independent monitoring and inspection

The World Health Organization (WHO) stated that COVID-19 “must not be used as a justification for objecting to external inspection of prisons and other places of detention”(p.6) (World Health Organization, 2020b). It was recommended that monitoring and inspection continue with due caution regarding infection prevention and control (Association for the Prevention of Torture, 2020; International Corrections and Prisons Association (ICPA), 2020), attention towards the justified and appropriate use of medical isolation and lockdown measures (Commonwealth Human Rights Initiative, 2020), and increased implementation of remote reporting and complaint mechanisms for people in custody and staff (International Corrections and Prisons Association (ICPA), 2020; New South Wales Government, 2020; United Nations Human Rights Office of the High Commissioner, 2020a; United Nations Human Rights Office of the High Commissioner, 2020b; World Organization Against Torture, 2020a). Guidance for remote inspection was developed (International Corrections and Prisons Association (ICPA), 2020).

### Domain 16: compensatory measures

Several publications recommended that COVID-19 prevention and control measures that further restrict the freedoms of people in custody be offset by compensatory measures that maintain the rehabilitative qualities of custody and an acceptable quality of life (e.g., increase frequency and/or time allowances for phone calls; access to virtual education, vocational programs, and free psychological support services). A comprehensive list of recommended compensatory measures is provided in Appendix S1.

### Domain 17: lifting control measures

A consistent recommendation was that measures restricting individual freedoms be in place only for the period required for public health purposes, and be lifted as soon as conditions allow (Danish Institute Against Torture, 2020a; Danish Institute Against Torture, 2020b; Royal College of Psychiatrists, 2020). Close monitoring of the local epidemiological context and local public health advice was recommended to inform decisions to lift or modify control measures (Communicable Diseases Network Australia, 2020; Danish Institute Against Torture, 2020a; Danish Institute Against Torture, 2020b; US Centres for Disease Control and Prevention, 2020a; US Centres for Disease Control and Prevention, 2020b; US Centres for Disease Control and Prevention, 2020c). Few concrete recommendations were made for the termination of prevention and control measures such as cohorting and changes to recreation, programming, and services. Recommendations for lifting restrictions on in-person visitation were context-dependent; they ranged from once screening and containment policies are in place (AMEND, 2020b), to after an outbreak is declared over (Communicable Diseases Network Australia, 2020), to as long as COVID-19 remains prevalent in the community (European Centre for Disease Prevention and Control, 2020a; United Nations Human Rights Office of the High Commissioner, 2020d). Recommendations for terminating medical isolation are provided in Appendix S1.

### Domain 18: learning systems and evaluative frameworks

Data-driven evaluation and policy analysis were recommended to assess the effectiveness of response measures in reducing COVID-19 infection, to understand their impacts on the health and human rights of people in custody and staff, and to identify beneficial policy changes to adopt in standard operations (AMEND, 2020b; Buchanan et al., 2020; Communicable Diseases Network Australia, 2020; Dalton et al., 2009; Martyn et al., 2020; Nature, 2020; New Zealand Office of the Ombudsman, 2020). Regional and international knowledge sharing was recommended to inform future planning and response for similar health crises (AMEND, 2020b; Communicable Diseases Network Australia, 2020; United Nations Office on Drugs and Crime, 2020a) and to advocate for broader carceral system reform (Inter-Agency Standing Committee, 2020; Lachsz & Hurley, 2020; Mukherjee & El-Bassel, 2020; United Nations Office on Drugs and Crime, 2020a; World Organization Against Torture, 2020a). It was recommended that both people in custody and staff be involved in evaluation (AMEND, 2020b; Buchanan et al., 2020; Gagnon, 2020).

### Domain 19: key populations and settings

We identified several recommendations that were specific to key populations (women, elderly, Indigenous peoples, people with a disability, people who use drugs, and people with mental illness) and custodial settings (youth detention, immigration detention, forensic psychiatric facilities, and in low-middle income countries [LMIC]) (Appendix S1). Notably, this review found fewer recommendations specific to LMIC (Amnesty International, 2020a; Amnesty International, 2020b; European Centre for Disease Prevention and Control, 2020a; FIACAT, 2020), which may require more pragmatic interventions that account for resource constraints, and forensic psychiatry (Innovative Prison Systems, 2020; Simpson et al., 2020), where release may not be possible.

## Discussion

From over 200 eligible publications, we documented a considerable volume of recommendations to prevent and/or control COVID-19 in custodial settings during the first six months of 2020. In total, we identified 374 unique recommendations spanning 19 domains; each domain represented a distinct and important area to consider for a comprehensive COVID-19 response. We determined that, overall, comprehensive guidance was available. However, no individual publication addressed all identified domains.

Numerous publications called for immediate reductions in the number of people incarcerated, and a moratorium on immigration detention to address pre-existing crises of widespread overcrowding in custodial settings (Alohan & Calvo, 2020; American Academy of Pediatrics, 2020; Amnesty International, 2020a; Amnesty International, 2020b; Annie E. Casey Foundation, 2020; Council of Europe Commissioner for Human Rights, 2020a; Council of Europe Commissioner for Human Rights, 2020b; Henry, 2020; Piel, 2020; Rubenstein, 2020; Simpson & Butler, 2020; Sivashanker et al., 2020; Terres des hommes, 2020; The Alliance for Child Protection in Humanitarian Action, 2020; UNICEF, 2020; World Organization Against Torture, 2020b). These recommendations build on a strong history of advocacy to reduce prison overcrowding globally (Penal Reform International, 2019; World Health Organization, 2007), with COVID-19 starkly revealing the health and human rights implications of overcrowding. National prison systems in 124 countries exceed their maximum occupancy rate, with 22 countries reporting that their prisons contain over twice as many people as they were designed to house (Penal Reform International, 2020b). Overcrowding to this degree renders physical distancing and other infection prevention and control measures near impossible, severely inhibits access to healthcare, and undermines all aspects of COVID-19 response (Penal Reform International, 2020b). It was therefore unsurprising that several publications reinforced that the COVID-19 pandemic provides an unprecedented opportunity for carceral system reform to reduce overreliance on mass incarceration and create safer physical environments that enable the prevention and control of infectious disease (McKenzie & Mishori, 2020; Minkler et al., 2020). With a strong focus on prison depopulation during the COVID-19 pandemic, efforts to reduce populations in custody need to be matched with adequate supports that facilitate safe community reintegration after release (Franco-Paredes et al., 2021; Gorman & Ramaswamy, 2020; Montoya-Barthelemy et al., 2020; Mukherjee & El-Bassel, 2020; Piel, 2020; Shepherd & Spivak, 2020; United Nations Network on Migration, 2020).

### Conflicting recommendations

Four main conflicts in recommendations were identified. First, one guideline recommended the temporary suspension of judicial hearings with exception of remote hearings for urgent cases, a recommendation implemented in Brazil, Latvia, the Netherlands, and Pakistan (Innovative Prison Systems, 2020). However, no information was provided to outline when judicial hearings should be resumed or how to distinguish ‘urgent’ cases from ‘non-urgent’ cases. This contrasted with the majority of publications on this topic calling for the continuation of all hearings during the pandemic (Association for the Prevention of Torture, 2020; Tahrir Institute for Middle East Policy (TIMEP) and Middle East and North Africa (MENA) Rights Group, 2020; The Alliance for Child Protection in Humanitarian Action, 2020; The International Legal Foundation, 2020; United Nations, 2020), including the immediate establishment of emergency courts to reduce the number of unsentenced people held in pre-trial detention (Inter-Agency Standing Committee, 2020). Second, we identified mixed recommendations regarding in-person visitation. The restriction of all non-essential visitors from entering custodial settings was recommended in several publications on this topic (AMEND, 2020b; Innovative Prison Systems, 2020; Penal Reform International, 2020a; Prison department of the Republic of Lithuania, 2020; Seal, 2020). However, several others cautiously recommended some in-person visitation with protective measures in place (Innovative Prison Systems, 2020; Justice and Corrections Service, 2020; US Centres for Disease Control and Prevention, 2020a; US Centres for Disease Control and Prevention, 2020b; US Centres for Disease Control and Prevention, 2020c; World Organization Against Torture, 2020a), and one guideline (National Police Chief’s Council, 2020) recommended that essential visitors, including parents of youth in custody and legal representatives, be permitted to visit in-person. Third, several publications recommended the use of routine quarantine of new facility admissions regardless of COVID-19 infection or exposure (Centers for Disease Control and Prevention, 2020; U.S. Immigration and Customs Enforcement, 2020; Njuguna et al., 2020; O’Moore & Farrar, 2020; US Centres for Disease Control and Prevention, 2020b), while two publications (Lachsz & Hurley, 2020; World Health Organization, 2020b) discouraged its use due to potentially harmful mental health impacts and preference towards comprehensive testing and screening. Lastly, three publications recommended the use of fines as an alternative to incarceration (Danish Institute Against Torture, 2020a; Danish Institute Against Torture, 2020b; Seal, 2020; The International Legal Foundation, 2020) whereas others expressed concerns about the disproportionate impacts of these measures on Indigenous peoples (National Aboriginal & Torres Strait Islander Legal Services, 2020b) and people living in poverty (United Nations Office on Drugs and Crime (UNODC), 2020b).

### Key gaps identified

Definitive guidance on when to remove or reduce the intensity of COVID-19 prevention and control measures was notably absent, except for terminating medical isolation and declaring an outbreak to be over. While there was clear consensus that these decisions should be based upon close monitoring of the local epidemiological context and public health advice, no specific benchmarks (e.g., local infection rates) were provided in any of the included publications. Furthermore, there was limited guidance over who should be responsible for these decisions; designated officials (US Centres for Disease Control and Prevention, 2020b), correctional authorities (Danish Institute Against Torture, 2020a; Danish Institute Against Torture, 2020b; United Nations Office on Drugs and Crime, 2020d), and outbreak management teams (Communicable Diseases Network Australia, 2020) were identified. Clear and externally verifiable criteria to guide the de-escalation of COVID-19 prevention and control measures in custody are needed to ensure that they are not in place for longer than necessary. This is especially pertinent for measures that restrict personal freedoms (e.g., out-of-cell time) and social interaction (e.g., visitation), due to their profound impacts on the psychological wellbeing of people in custody (Hewson et al., 2020; Stewart et al., 2020). Another gap related to guidance for specific populations. We developed a sub-domain within the ‘Key populations and settings’ domain, when coding recommendations that were targeted for specific population subgroups. Recommendations for some groups were notably absent from this domain (e.g., LGBTQ people), were categorised broadly (e.g., Indigenous peoples), or provided minimal guidance (e.g. people with a disability). Guidance that addresses the specific needs of all people in custody and acknowledges local diversity and cultural differences within the broad subgroups identified in this review are needed. Finally, to our knowledge, few publications actively included the perspectives of people in custody, their families, or staff in the development of recommendations.

### Strengths and limitations

This review included a wide range of recommendations spanning multiple custodial settings and population subgroups, identified from a systematic search of both peer-reviewed and grey literature (Appendix S3). Our broad approach allowed for the inclusion of guidance from various stakeholders represented in the COVID-19 response, and consideration of broader social, physical, and mental health needs of people in custody and staff. However, there are some limitations. First, although English is an official language of key international bodies that contributed 34% of included publications, our decision to restrict the search strategy to English-language publications may have led to exclusion of some publications from LMIC. Second, our search strategy targeted publications explicitly focused on COVID-19 and custodial settings. Recommendations relevant to custodial settings that were made in broader pandemic response publications were therefore not included in this review. Third, this review focused on initial guidance from the first 6 months of the pandemic. It therefore did not include recommendations from emergent areas such as COVID-19 vaccination or variants of concern. Recommendations identified in this review reflect information at the time of their publication – when governments around the world were out of necessity mounting a rapid response to the pandemic – and should be considered within the context of evolving COVID-19 knowledge.

### Implementation and next steps

Comprehensive guidance is necessary, but not sufficient, for effective COVID-19 response. Custodial settings and systems vary widely in their structure, stability, resource availability, and cultural context, and are impacted by external factors including political will and the broader public health response to COVID-19, which greatly influence the actions of governments and correctional authorities. Despite the availability of comprehensive guidance early in the pandemic, early reports have suggested that the global response to COVID-19 in custodial settings has largely been inadequate. A recent review of 69 countries’ response to COVID-19 in custodial settings identified shortages of testing capacity, lack of preventive and protective measures, insufficient rates of release to address overcrowding, and inappropriate use of solitary confinement (Amnesty International, 2021).

We have identified several priorities to address this implementation gap that align with recommendations identified in this review. Firstly, there is an urgent need for reliable data collection, analysis, and public disclosure on basic epidemiology (e.g., numbers of infections and deaths) and responses (e.g., measures implemented) in custodial settings globally (Amnesty International, 2021). This information is rarely reported, but is critical to maximise transparency and hold governments and custodial authorities accountable for the health of people in custody and staff. Secondly, a comprehensive programme of research documenting and examining the implementation of recommended prevention and control measures in custodial settings, with due consideration to priority groups and LMIC, is needed to identify factors facilitating and inhibiting an effective COVID-19 response. To date, we are not aware of any published implementation studies. A fit-for-purpose Optional Protocol to the Convention Against Torture (OPCAT) (United Nations Human Rights Office of the High Commissioner, 2002) could act as a framework to assess the adequacy of the COVID-19 response from a human rights perspective. Finally, there remains a dearth of research evidence examining the effectiveness of recommended prevention and control measures, and combinations of measures, for reducing COVID-19 morbidity and mortality and associated impacts on people in custody and staff. This represents a critical knowledge gap that will be essential to optimising and adapting responses in custodial settings globally. The framework presented in this review (Table 2) is a useful starting point for organising this important body of work.

## Conclusions

A comprehensive response to COVID-19 in custodial settings is highly complex and must carefully balance the need for restrictive infection prevention and control strategies with their potentially harmful impacts on health and human rights. Despite the availability of comprehensive guidance early in the pandemic, important gaps remain in the implementation of recommended prevention and control measures globally, and in the availability of evidence assessing their effectiveness on reducing COVID-19 disease, impact on people in custody and staff, and implementation. Evaluation of the implementation of these measures in custodial settings, and their diverse impacts on health and wellbeing, are crucial next steps.

## Supplementary Information

**Additional file 1: Appendix S1.** List of recommendations by domain and sub-domain; Description of data: Appendix S1 provides a complete list of all 374 recommendations categorised by domain and sub-domain.

**Additional file 2: Appendix S2.** Summary of included publications; Description of data: Appendix S2 provides a complete list of all 201 eligible publications analysed in this review and provides information on (1) WHO region (country), (2) Date of publication, (3) Type of author, (4) Type of publication, (5) Targeted audience, and (6) Targeted setting.

**Additional file 3: Appendix S3.** Search strategy; Description of data: Appendix S3 provides the comprehensive search strategy to allow for reproduction of the search results.

## Data Availability

Data sharing is not applicable to this article because no datasets were generated or analysed during the current study. All publications included in this review are publicly available; a list is included in Appendix S2.

## Abbreviations

COVID-19: Coronavirus Disease 2019
WHO: World Health Organization
LMIC: Low-middle income countries
