# Correction to: A rapid review of early guidance to prevent and control COVID-19 in custodial settings

**DOI:** 10.1186/s40352-021-00160-8

**Published:** 2021-11-22

**Authors:** Lindsay A. Pearce, Alaina Vaisey, Claire Keen, Lucas Calais-Ferreira, James A. Foulds, Jesse T. Young, Louise Southalan, Rohan Borschmann, Ruth Gray, Sunita Stürup-Toft, Stuart A. Kinner

**Affiliations:** 1 Justice Health Unit, Melbourne School of Population and Global Health, University of Melbourne, Level 4, 207 Bouverie Street, Carlton, Victoria 3053 Australia; 2 Centre for Adolescent Health, Murdoch Children’s Research Institute, Melbourne, Victoria Australia; 3 Mater Research Institute, University of Queensland, Brisbane, Queensland Australia; 4 Department of Psychological Medicine, University of Otago, Christchurch, New Zealand; 5 School of Population and Global Health, The University of Western Australia, Perth, Western Australia Australia; 6 National Drug Research Institute, Curtin University, Perth, Western Australia Australia; 7 Law School, University of Western Australia, Perth, Western Australia Australia; 8 Health Service and Population Research Department, Institute of Psychiatry, Psychology and Neuroscience, King’s College London, London, UK; 9 Melbourne School of Psychological Sciences, The University of Melbourne, Melbourne, Victoria Australia; 10 Healthcare in Prison, South Eastern Health and Social Care Trust, Belfast, North, Ireland, UK; 11 Global Public Health, Public Health England, London, England; 12 Griffith Criminology Institute, Griffith University, Brisbane, Queensland Australia; 13 School of Public Health and Preventive Medicine, Monash University, Melbourne, Victoria Australia

**Correction to:** **Health Justice** **9, 27 (2021).**

**https://doi.org/10.1186/s40352-021-00150-w**

Following the publication of the original article (Pearce et al., 2021) the authors noticed that there are still issues of concern with the publication. The following were flagged by the authors at the proof stage that have still not been addressed.

- There are multiple, repeated references for ‘United Nations Office on Drugs and Crime’ in the reference list.
- These include:
  - (2 instances) United Nations Office on Drugs and Crime (UNODC). (2015). The United Nations Standard Minimum Rules for the Treatment of Prisoners (the Nelson Mandela Rules). Retrieved from https://www.unodc.org/documents/justice-and-prison- reform/Nelson_Mandela_Rules-E-ebook.pdf
  - (2 instances) United Nations Office on Drugs and Crime (UNODC). (2020). COVID-19 prevention and control among people working in prison
- In the reference list, ‘Retrieved from’ still remains in many instances where there is no URL; we had asked for these to be removed prior to publication.
- Supplementary appendix 1 is the version submitted with tracked changes (highlights), formatting as requested in the review process. These highlights should have been removed in the publication process, once approved by reviewers.

The original article (Pearce et al., 2021) has been updated.

## Supplementary Information

**Additional file 1: Appendix S1.** List of recommendations by domain and sub-domain.

## References

[CR1] Pearce LA, Vaisey A, Keen C, Calais-Ferreira L, Foulds JA, Young JT, Southalan L, Borschmann R, Gray R, Stürup-Toft S, Kinner SA (2021). A rapid review of early guidance to prevent and control COVID-19 in custodial settings. Health Justice.

