# Supplementary material for: A rapid review of early guidance to prevent and control COVID-19 in custodial settings
Source: Health Justice. 2021 Oct 15;9:27. doi: 10.1186/s40352-021-00150-w (PMC8518275; doi:10.1186/s40352-021-00150-w)
Supplement: Supplementary file 2 — Additional file 2: Appendix S2. Summary of included publications; Description of data: Appendix S2 provides a complete list of all 201 eligible publications analysed in this review and provides information on (1) WHO region (country), (2) Date of publication, (3) Type of author, (4) Type of publication, (5) Targeted audience, and (6) Targeted setting. [file 40352_2021_150_MOESM2_ESM.docx]

**Appendix S2** Summary of included publications

| **Author [reference]** | **WHO region (country*)** | **Date of publication (if available)** | **Type of author** | **Type of publication** | **Targeted audience**** | **Targeted setting***** |
| --- | --- | --- | --- | --- | --- | --- |
| Aboriginal Legal Service[1] | Western Pacific (Australia) | 29-Apr-20 | RO | GL - statement/letter | National | P |
| Akiyama et al.[2] | Not specific | 28-May-20 | A | PR - commentary | International | P |
| Alexander et al.[3] | Not specific |  | A | PR - commentary | International | P |
| Alohan et al.[4] | Not specific | 3-Jun-20 | A | PR - commentary | International | NS |
| AMEND 1[5] | Americas (USA) | 30-Mar-20 | RO | GL - clinical guideline | International | P |
| AMEND 2[6] | Americas (USA) | 23-Mar-20 | RO | GL - general guidance | International | P |
| AMEND 3[7] | Americas (USA) | 9-Apr-20 | RO | GL - general guidance | International | P |
| AMEND 4[8] | Americas (USA) | 30-Mar-20 | RO | GL - general guidance | National | P |
| American Academy of Pediatrics[9] | Americas (USA) | 7-Jun-20 | RO | GL - general guidance | International | YD |
| American Civil Liberties Union[10] | Americas (USA) |  | RO | GL - report | National | P |
| Amnesty International 1[11] | Africa (Tanzania) | 20-May-20 | RO | GL - statement/letter | National | P |
| Amnesty International 2[12] | Europe (Turkey) | 30-Mar-20 | RO | GL - statement/letter | National | P |
| Amnesty International 3[13] | Africa (Madagascar) | 3-Apr-20 | RO | GL - statement/letter | National | P |
| Amnesty International & Justice Project Pakistan[14] | Eastern Mediterranean (Pakistan) |  | RO | GL - statement/letter | National | P |
| Amon et al.[15] | Americas (USA) | 23-Mar-20 | A | GL - report | International | NS |
| Annie E. Casey Foundation[16] | Americas (USA) | 6-Apr-20 | RO | GL - general guidance | National | YD |
| Association for the Prevention of Torture[17] | Europe |  | RO | GL - general guidance | International | NS |
| Australian and New Zealand Scholars[18] | Western Pacific (Australia) |  | A | GL - statement/letter | National | P, YD |
| Australian Scholars[19] | Western Pacific (Australia) |  | A | GL - statement/letter | National | P, YD |
| Avocats Sans Frontières[20] | Africa |  | RO | GL - statement/letter | International | P |
| Bar Human Rights Committee of England & Wales[21] | Europe (Turkey) | 3-Apr-20 | RO | GL - statement/letter | National | P |
| Barnert et al.[22] | Americas (USA) | Jul-20 | A | PR - commentary | National | NS |
| Buchanan et al.[23] | Americas (USA) | 23-Jun-20 | A | PR - commentary | National | YD |
| Caputo et al.[24] | Not specific |  | A | PR - commentary | International | NS |
| Centres for Disease Control and Prevention[25] | Americas (USA) | 9-Apr-20 | G | GL - general guidance | National | NS |
| Child Rights International Network[26] | Not specific | 26-Mar-20 | RO | GL - general guidance | International | YD |
| Children of Prisoners Europe[27] | Europe | May-20 | RO | GL - general guidance | International | NS |
| Clarke et al.[28] | Europe (Ireland) |  | A | PR - original research | International | P |
| Commonwealth Human Rights Initiative[29] | Not specific (Commonwealth) |  | RO | GL - general guidance | International | P |
| Centres for Disease Control and Prevention[30] | Americas (USA) | 23-Mar-20 | RO | GL - general guidance | National | NS |
| Communicable Diseases Network Australia[31] | Western Pacific (Australia) | 31-Mar-20 | RO | GL - general guidance | National | NS |
| Council of Europe 1[32] | Europe | 17-Apr-20 | G | GL - statement/letter | International | P, CPP, LP |
| Council of Europe 2[33] | Europe | 26-Mar-20 | G | GL - statement/letter | International | ID |
| Council of Europe 3[34] | Europe | 6-Apr-20 | G | GL - statement/letter | International | P |
| Crowley et al.[35] | Not specific | 10-Jun-20 | A | PR - commentary | International | P |
| Danish Institute Against Torture 1[36] | Europe (Denmark) | Jun-20 | RO | GL - general guidance | International | P |
| Danish Institute Against Torture 2[37] | Europe (Denmark) | 4-May-20 | RO | GL - general guidance | International | P |
| Drogin, E.[38] | Not specific | 4-Jun-20 | A | PR - commentary | International | NS |
| Emmanuel et al.[39] | Not specific |  | A | GL - report | International | ID |
| Emory Center for the Health of Incarcerated Persons[40] | Americas (USA) | 20-Mar-20 | A | GL - general guidance | National | P, ID |
| European Prison Litigation Network[41] | Europe |  | RO | GL - general guidance | International | P |
| European Centre for Disease Prevention and Control 1[42] | Europe | 15-Jun-20 | G | GL - general guidance | International | ID |
| European Centre for Disease Prevention and Control 2[43] | Europe | 3-Jul-20 | G | GL - general guidance | International |  |
| European Committee for the Prevention of Torture and Inhuman or Degrading Treatment or Punishment[44] | Europe | 20-Mar-20 | G | GL - statement/letter | International | NS |
| Executives Transforming Probation & Parole[45] | Americas (USA) |  | RO | GL - statement/letter | National | CPP |
| Fair and Just Prosecution[46] | Americas (USA) | 25-Mar-20 | RO | GL - statement/letter | National | NS |
| FIACAT[47] | Africa | 24-Mar-20 | RO | GL - statement/letter | International | NS |
| Franco-Paredes et al.[48] | Not specific | 22-Jun-20 | A | PR - commentary | International | P |
| Fróis, C.[49] | Not specific | Jun-20 | A | PR - commentary | International | P |
| Gagnon, J.C.[50] | Americas (USA) |  | A | PR - letter | National | YD |
| Garcini et al.[51] | Americas (USA) | 13-May-20 | A | PR - commentary | National | YD |
| General Directorate “Execution of Sentences” Bulgaria[52] | Europe (Bulgaria) |  | G | GL - general guidance | National | NS |
| Gorman et al.[53] | Americas (USA) |  | A | PR - opinion editorial | Local | P |
| Government of Canada Office of the Correctional Officer 1[54] | Americas (Canada) | 23-Apr-20 | G | GL - report | National | P |
| Government of Canada Office of the Correctional Officer 2[55] | Americas (Canada) | 19-Jun-20 | G | GL - report | National | P |
| Gulati et al.[56] | Not specific |  | A | PR - commentary | International | P |
| Hargreaves et al.[57] | Europe | 26-Mar-20 | A | PR - opinion editorial | International | ID |
| Hawks et al.[58] | Americas (USA) | 28-Mar-20 | A | PR - opinion editorial | International | P |
| Henry, B.F.[59] | Not specific |  | A | PR - commentary | International | NS |
| Hewson et al.[60] | Not specific | Jul-20 | A | PR - commentary | International | NS |
| HM Inspectorate of Prisons[61] | Europe (UK) | 20-Apr-20 | G | GL - clinical guidance | National | P, YD |
| HM Prison & Probation Service[62] | Europe (UK) | 28-Apr-20 | G | GL - general guidance | National | P, FP |
| Human Rights First[63] | Americas (USA) | 17-Mar-20 | RO | GL - statement/letter | National | ID |
| Human Rights Law Centre[64] | Western Pacific (Australia) |  | RO | GL - general guidance | National | P, YD |
| Human Rights Watch 1[65] | Americas (USA) |  | RO | GL - general guidance | National | P |
| Human Rights Watch 2[66] | Not specific |  | RO | GL - general guidance | International | P, NS |
| Human Rights Watch 3[67] | Not specific | 19-Mar-20 | RO | GL - general guidance | International | P, ID, LP |
| Independent Advisory Panel on Deaths in Custody[68] | Europe (UK) | 1-Jun-20 | G | GL - report | National | P |
| Innovative Prison Systems[69] | Not specific |  | RO | GL - general guidance | International | P, YD, FP |
| Inspector of Custodial Services[70] | Western Pacific (Australia) |  | G | GL - official plan | Local | P, YD |
| Inter-agency Standing Committee[71] | Not specific | Mar-20 | IGO | GL - general guidance | International | NS |
| International Committee of the Red Cross[72] | Not specific |  | IGO | GL - general guidance | International | NS |
| International Corrections and Prisons Association[73] | Not specific | 20-Apr-20 | RO | GL - general guidance | International | P |
| International Detention Coalition[74] | Not specific |  | RO | GL - statement/letter | International | ID |
| International Drug Policy Consortium[75] | South-East Asia | Apr-20 | RO | GL - policy brief | International | P, NS |
| International Federation for Human Rights 1[76] | Not specific | 7-Apr-20 | RO | GL - general guidance | International | NS |
| International Federation for Human Rights 2[77] | Not specific | 22-Apr-20 | RO | GL - statement/letter | International | P |
| International Federation for Human Rights 3[78] | Eastern Mediterranean (Palestine) | 17-Apr-20 | RO | GL - statement/letter | National | P, NS |
| International Federation for Human Rights 4[79] | South-East Asia (Thailand) | 15-Apr-20 | RO | GL - statement/letter | National | P |
| Irvine et al.[80] | Americas (USA) | 15-May-20 | A | PR - original research | National | ID |
| Italian Department of Prison Administration[81] | Europe (Italy) | 13-Mar-20 | G | GL - general guidance | National | P, YD |
| Johnson, S.[82] | Americas (USA) |  | RO | GL - report | National | CPP |
| Justice and Corrections Service[83] | Not specific |  | IGO | GL - general guidance | International | P |
| Keller et al.[84] | Americas (USA) | 31-Mar-20 | A | PR - commentary | National | ID |
| Kinner et al.[85] | Not specific | 17-Mar-20 | A | PR - commentary | International | NS |
| Knox, C.M.[86] | Not specific | Jun-20 | A |  | International | NS |
| Kothari et al.[87] | Europe (UK) |  | A | PR - opinion editorial | National | NS |
| Lachsz et al.[88] | Western Pacific (Australia & New Zealand) | 27-May-20 | IGO | GL - statement/letter | International | P, YD, NS |
| León et al.[89] | Not specific | 24-Jun-20 | A | PR - commentary | International | YD |
| Liebrenz et al.[90] | Not specific |  | A | PR - commentary | International | NS |
| Malta et al.[91] | Europe (Italy) |  | A | PR - commentary | National | NS |
| Manthorpe et al.[92] | Western Pacific (Australia) | 1-Jul-20 | G | GL - statement/letter | National | ID |
| Marcum, C.D.[93] | Americas (USA) | 7-Jun-20 | A | PR - commentary | National | NS |
| Martyn et al.[94] | Americas (USA) | Jun-20 | A | GL - policy brief | National | NS |
| Mauer, M.[95] | Americas (USA) | 2-Jun-20 | RO | GL - statement/letter | National | P |
| McKenzie et al.[96] | Americas (USA) | 15-Jun-20 | A | PR - commentary | National | ID |
| Meyer et al.[97] | Americas (USA) | 15-Apr-20 | A | PR - commentary | National | ID |
| Mijatovic, D.[98] | Europe | 26-Mar-20 | G | GL - statement/letter | International | ID |
| Minkler et al.[99] | Americas (USA) |  | A | PR - commentary | National | NS |
| Minnesota Department of Health[100] | Americas (USA) | 1-May-20 | G | GL - general guidance | Local | P |
| Montoya-Barthelemy et al.[101] | Not specific | Jun-20 | A | PR - commentary | International | P |
| Mukherjee et al.[102] | Americas (USA) | 11-Jun-20 | A | PR - commentary | National | P |
| National Aboriginal & Torres Strait Islander Legal Services 1[103] | Western Pacific (Australia) | 8-Apr-20 | RO | GL - statement/letter | National | NS |
| National Aboriginal & Torres Strait Islander Legal Services 2[104] | Western Pacific (Australia) | 23-Mar-20 | RO | GL - statement/letter | National | P, YD |
| National Aboriginal & Torres Strait Islander Legal Services 3[105] | Western Pacific (Australia) | 1-May-20 | RO | GL - press release | National | P, NS |
| National Commission on Correctional Health Care 1[106] | Americas (USA) |  | RO | GL - general guidance | National | NS |
| National Commission on Correctional Health Care 2[107] | Americas (USA) | 27-Mar-20 | RO | GL - clinical guidance | National | NS |
| National Commission on Correctional Health Care 3[108] | Americas (USA) | 27-Mar-20 | RO | GL - general guidance | National | NS |
| National Commission on Correctional Health Care 4[109] | Americas (USA) | 27-Mar-20 | RO | GL - general guidance | National | NS |
| National Commission on Correctional Health Care 5[110] | Americas (USA) | 27-Mar-20 | RO | GL - general guidance | National | NS |
| National Council on Crime & Delinquency[111] | Not specific |  | RO | GL - general guidance | International | CPP |
| National Juvenile Defender Center[112] | Americas (USA) | 27-Mar-20 | RO | GL - general guidance | National | LP |
| National Police Chiefs' Council[113] | Europe (UK) | 8-Apr-20 | RO | GL - general guidance | National |  |
| Nature[114] | Not specific | 19-May-20 | A | PR - opinion editorial | International | NS |
| Njuguna et al.[115] | Americas (USA) | 29-Jun-20 | A | PR - original research | Local | P |
| Norwegian Institute of Public Health[116] | Europe (Norway) | 24-Jun-20 | G | GL - general guidance | National | NS |
| Nowotny et al.[117] | Americas (USA) | Jul-20 | A | PR - opinion editorial | National | P |
| Office of the Ombudsman[118] | Western Pacific (New Zealand) | Jun-20 | G | GL - report | National | FP |
| OHCHR OPT, ICHR, and the Palestinian Ministry of the Interior[119] | Eastern Mediterranean (Palestine) |  | IGO | GL - general guidance | National | LP, NS |
| Okano & Blower[120] | Americas (USA) | 16-May-20 | A | PR - letter | National | P |
| Okonkwo et al.[121] | Americas (USA) | 3-Jun-20 | A | PR - commentary | National | P, ID |
| O'Moore & Farrar[122] | Europe (UK) | 24-Apr-20 | G | GL - report | National | P |
| Openshaw & Travassos 1[123] | Americas (USA) | 31-May-20 | A | PR - commentary | National | ID |
| Openshaw & Travassos 2[124] | Americas (USA) | 5-Aug-20 | A | PR - commentary | National | NS |
| Orcutt et al.[125] | Not specific | 27-Apr-20 | A | PR - letter | International | ID |
| Page et al.[126] | Americas (USA) | 21-May-20 | A | PR - commentary | National | ID |
| Pan American Health Organization[127] | Americas |  | IGO | GL - general guidance | International | NS |
| Penal Reform International 1[128] | Not specific | 16-Mar-20 | RO | GL - report | International | P |
| Penal Reform International 2[129] | Not specific | Apr-20 | RO | GL - report | International | P, CPP |
| Piel, J.[130] | Americas (USA) | Jul-20 | A | PR - letter | National | CPP |
| Prison Department of the Republic of Lithuania[131] | Europe (Lithuania) | 19-Mar-20 | G | GL - official plan | National | P |
| Protection International[132] | South-East Asia (Thailand) | 15-Apr-20 | RO | GL - statement/letter | National | NS |
| Public Health England 1[133] | Europe (UK) | 20-May-20 | G | GL - general guidance | National | P, NS |
| Public Health England 2[134] | Europe (UK) | 4-Aug-20 | G | GL - general guidance | National | NS |
| Pyrooz et al.[135] | Americas (USA) | 12-Jun-20 | A | PR - original research | National | P |
| Refugee Council of Australia[136] | Western Pacific (Australia) | 9-Apr-20 | RO | GL - general guidance | National | ID, CPP, LP |
| Refugees International[137] | Not specific |  | RO | GL - report | International | ID, CPP, LP |
| Reinhardt & Chen[138] | Americas (USA) | Aug-20 | A | PR - original research | National | P, CPP |
| Royal College of General Practitioners Secure Environments Group[139] | Europe (UK) | 20-Apr-20 | RO | GL - clinical guidance | National | P, NS |
| Royal College of Psychiatrists[140] | Europe (UK) |  | RO | GL - general guidance | National | P, FP |
| Rubenstein, L.[141] | Americas (USA) | 25-Mar-20 | RO | GL - statement/letter | Local | P, YD |
| Sánchez et al.[142] | Americas (Brazil) | 8-May-20 | A | PR - commentary | National | P |
| Seal, D.[143] | Americas (USA) | Mar-20 | A | PR - commentary | International | P, YD |
| Shepherd & Spivak[144] | Western Pacific (Australia) | 22-Jun-20 | A | PR - commentary | National | P |
| Simpson et al.[145] | Americas (Canada) | 23-Jun-20 | A | PR - original research | International | FP |
| Simpson & Butler[146] | Not specific | 20-Apr-20 | A | PR - opinion editorial | International | P |
| Síndic El Defensor de les Personas[147] | Europe (Spain) | 3-Apr-20 | G | GL - statement/letter | Local | NS |
| Sivashanker et al.[148] | Americas (USA) | 29-Apr-20 | A | PR - commentary | National | P |
| Special Rapporteur on Extrajudicial, Summary or Arbitrary Killings[149] | Not specific |  | IGO | GL - general guidance | International | NS |
| Stewart et al.[150] | Western Pacific (Australia) | 22-Jun-20 | A | PR - letter | International | P, YD |
| Subcommittee on Prevention of Torture and Other Cruel, Inhuman or Degrading Treatment or Punishment[151] | Not specific | 25-Mar-20 | IGO | GL - general guidance | International | NS |
| Surprenant, C.[152] | Americas (USA) | 27-Mar-20 | A | PR - commentary | National |  |
| Syrian Network for Human Rights[153] | Eastern Mediterranean (Syria) | 8-May-20 | RO | GL - report | National | NS |
| The Tahrir Institute for Middle East Policy[154] | Eastern Mediterranean | 23-Mar-20 | RO | GL - statement/letter | International | NS |
| Terre des hommes[155] | Not specific | 27-Mar-20 | RO | GL - statement/letter | International | YD, ID |
| The Alliance for Child Protection in Humanitarian Action[156] | Not specific |  | RO | GL - general guidance | International | NS |
| The Bail Project[157] | Americas (USA) |  | RO | GL - statement/letter | National | P |
| The International Legal Foundation[158] | Not specific |  | RO | GL - general guidance | International | NS |
| The Kirby Institute[159] | Western Pacific (Australia) | 16-Apr-20 | RO | GL - report | Local | P, YD, ID |
| UNHCR, IOM, OHCHR, WHO[160] | Not specific |  | IGO | GL - press release | International | ID |
| UN Women, IDO, UNDP, UNODC, World Bank, Pathfinders[161] | Not specific | May-20 | IGO | GL - report | International | NS |
| UNAIDS[162] | Not specific |  | IGO | GL - report | International | NS |
| UNICEF 1[163] | Not specific | 13-Apr-20 | IGO | GL - statement/letter | International | NS |
| UNICEF 2[164] | Eastern Mediterranean (Palestine & Israel) | 11-May-20 | IGO | GL - statement/letter | National | YD |
| UNICEF 3[165] | Not specific |  | IGO | GL - general guidance | International | ID |
| United Nations[166] | Not specific | Apr-20 | IGO | GL - general guidance | International | NS |
| UNODC, PAHO/WHO, OCHCR Mexico[167] | Not specific | Mar-20 | IGO | GL - general guidance | International | P |
| United Nations Human Rights Office of the High Commissioner 1[168] | Not specific | 20-Mar-20 | IGO | GL - press release | International | ID |
| United Nations Human Rights Office of the High Commissioner 2[169] | Americas | 5-May-20 | IGO | GL - press release | International | P, YD, ID, NS |
| United Nations Human Rights Office of the High Commissioner 3[170] | Not specific | 25-Mar-20 | IGO | GL - press release | International | NS |
| United Nations Human Rights Office of the High Commissioner 4[171] | Not specific | 7-Apr-20 | IGO | GL - general guidance | International | ID |
| United Nations Human Rights Office of the High Commissioner 5[172] | Americas (USA) | 29-May-20 | IGO | GL - press release | National | NS |
| United Nations Institute for Training and Research[173] | Not specific |  | IGO | GL - general guidance | International | NS |
| United Nations Network on Migration 1[174] | Not specific |  | IGO | GL - general guidance | International | ID |
| United Nations Network on Migration 2[175] | Not specific | 13-May-20 | IGO | GL - statement/letter | International | ID |
| United Nations Office on Drugs and Crime 1[176] | Not specific |  | IGO | GL - general guidance | International | P |
| United Nations Office on Drugs and Crime 2[177] | Not specific |  | IGO | GL - general guidance | International | P |
| United Nations Office on Drugs and Crime 3[178] | Not specific | May-20 | IGO | GL - general guidance | International | LP |
| United Nations Office on Drugs and Crime 4[179] | Not specific | 31-Mar-20 | IGO | GL - statement/letter | International | P, YD |
| United Nations Office on Drugs and Crime 5[180] | Not specific | 6-Apr-20 | IGO | GL - general guidance | International | YD |
| United Nations Office on Drugs and Crime 6[181] | Not specific |  | IGO | GL - general guidance | International | NS |
| University of Oxford Faculty of Law[182] | Europe (UK) | 13-May-20 | RO | GL - statement/letter | National | ID |
| Vera Institute of Justice 1[183] | Americas (USA) | 24-Mar-20 | RO | GL - general guidance | National | YD |
| Vera Institute of Justice 2[184] | Americas (USA) | 11-May-20 | RO | GL - general guidance | International | NS |
| Vose et al.[185] | Americas (USA) | 19-Jun-20 | A | PR - commentary | National | P |
| Wagner & Widra[186] | Americas (USA) | 27-Mar-20 | RO | GL - statement/letter | National | P |
| Wallace et al. 1[187] | Americas (USA) | 6-May-20 | A | PR - original research | National | P |
| Wallace et al. 2[188] | Americas (USA) | 15-May-20 | A | PR - original research | National | NS |
| Waly et al.[189] | Not specific | 13-May-20 | IGO | GL - statement/letter | International | NS |
| Wang et al.[190] | Not specific |  | A | PR - commentary | International | P |
| Working Group on Arbitrary Detention[191] | Not specific | 8-May-20 | IGO | GL - general guidance | International | ID, NS |
| World Health Organization 1[192] | Not specific |  | IGO | GL - general guidance | International | NS |
| World Health Organization 2[193] | Not specific |  | IGO | GL - general guidance | International | NS |
| World Health Organization 3[194] | Not specific | 15-Mar-20 | IGO | GL - general guidance | International | NS |
| World Organisation Against Torture 1[195] | Not specific | 15-Apr-20 | IGO | GL - policy brief | International | NS |
| World Organisation Against Torture 2[196] | Not specific | Mar-20 | IGO | GL - statement/letter | International | NS |
| World Organisation Against Torture 3[197] | South-East Asia (Philippines) | 6-Apr-20 | IGO | GL - statement/letter | National | P |
| World Organisation Against Torture 4[198] | Europe (Turkey) | 31-Mar-20 | IGO | GL - statement/letter | National | P |
| Wurcel et al.[199] | Americas (USA) | 1-Aug-20 | A | PR - letter | National | P |
| Yang & Thompson[200] | Western Pacific (China) | 2-Apr-20 | A | PR - letter | National | P |
| Youth Correctional Leaders for Justice[201] | Americas (USA) |  | RO | GL - statement/letter | International | YD |
| *Abbreviations:* WHO: world health organisation, RO: relevant organisation, A: academic, G: government, IGO: inter-governmental organisation/specialised agency, GL: grey literature, PR: peer-reviewed literature, P: prison, NS: not specific, YD: youth detention, CPP: community/probation/parole, LP: legal process, ID: immigration detention, FP: forensic psychiatric  * Country specified when applicable  ** *International* refers to recommendations without directive to a specified region. *Local* refers to recommendations made with directive towards provincial, state, territory, or lower level of locality.  *** Reviewing author coded each document as providing recommendations pertaining to: prisons, youth detention, immigration detention, forensic psychiatric, community/probation/parole, court/legal process, other, not specified. All applicable custodial settings were noted. Not specific (NS) referred to documents providing recommendations for ‘deprivation of liberty’ or ‘detention’ without specifying type of custodial setting. | | | | | | |

**References Appendix S2**

1. Aboriginal Legal Service. *Open letter: Urgent action from Australian Governments needed to prevent COVID-19 Black deaths in custody*. 2020 29 April 2020 [cited 2020 3 July 2020]; Available from: <https://www.alsnswact.org.au/open_letter_from_families_clean_out_prisons>.

2. Akiyama, M.J., A.C. Spaulding, and J.D. Rich, *Flattening the curve for incarcerated populations: Covid-19 in jails and prisons.* New England Journal of Medicine, 2020. **382**(22): p. 2075-2077.

3. Alexander, A.A., H. Allo, and H. Klukoff, *Sick and Shut In: Incarceration During a Public Health Crisis.* Journal of Humanistic Psychology, 2020: p. 0022167820930556.

4. Alohan, D. and M. Calvo, *COVID-19 Outbreaks at Correctional Facilities Demand a Health Equity Approach to Criminal Justice Reform.* Journal of Urban Health-Bulletin of the New York Academy of Medicine: p. 6.

5. AMEND, *Clinical Guidelines for COVID-19 in Correctional Settings.* 2020.

6. AMEND, *COVID-19 in Correctional Settings: Unique Challenges and Proposed Responses.* 2020.

7. AMEND, *The Ethical Use of Medical Isolation – Not Solitary Confinement – to Reduce COVID-19 Transmission in Correctional Settings*, D. Cloud, et al., Editors. 2020, AMEND.

8. Williams, B., et al., *Immediate Population Reduction Recommendations*. 2020, AMEND.

9. American Academy of Pediatrics, *Responding to the Needs of Youth Involved With the Justice System During the COVID-19 Pandemic.* 2020.

10. American Civil Liberties Union, *COVID-19 model finds nearly 100,000 more deaths than current estimates, due to failure to reduce jails*. 2020, ACLU.

11. Amnesty International, *Joint CSO letter to President Magufuli on the rights of prison detainees in Tanzania during the COVID-19 pandemic* 2020.

12. Amnesty International, *Joint public statement: Turkey: Rights groups call for urgent release of imprisoned journalists, human rights defenders and others, now at risk of COVID-19*. 2020, Amnesty International.

13. Amnesty International, *Madagascar: Authorities should reduce prison population as mitigation against COVID-19* 2020.

14. Amnesty International and Justice Project Pakistan, *Joint statement: Protect prisoners during COVID-19 outbreak*. 2020.

15. Amon, J.J., *COVID-19 and detention: Respecting human rights.* Health and Human Rights Journal, 2020.

16. Annie E. Casey Foundation, *Juvenile Justice Priorities During and After the COVID-19 Pandemic.* 2020.

17. Association for the Prevention of Torture, *Guidance: Monitoring Places of Detention through the COVID-19 Pandemic*. 2020.

18. Australia New Zealand Scholars, *Open letter to Australian governments on COVID-19 and the criminal justice system*. 2020.

19. Australian Scholars, *Second national open letter to Australian governments on COVID-19 and prisons and youth detention centres* 2020.

20. Avocats Sans Frontières, *The spread of COVID-19 requires urgent and immediate measures to be taken to protect the rights of detainees in Africa: Joint Statement adressed to the member states of the African Union and to human rights international organisations in Africa*. 2020.

21. Bar Human Rights Committee of England & Wales, *Political prisoners in Turkey in the face of the COVID-19 pandemic*. 2020, BHRC: London, UK.

22. Barnert, E., C. Ahalt, and B. Williams, *Prisons: Amplifiers of the COVID-19 Pandemic Hiding in Plain Sight.* American Journal of Public Health, 2020. **110**(7): p. 964-966.

23. Buchanan, M., et al., *It’s F** ing Chaos: COVID-19’s Impact on Juvenile Delinquency and Juvenile Justice.* American Journal of Criminal Justice, 2020: p. 1-23.

24. Caputo, F., et al., *Covid-19 emergency in prison: Current management and forensic perspectives.* Medico-Legal Journal, 2020: p. 0025817220923693.

25. Centers for Disease Control and Prevention, *FAQs for administrators, staff, people who are incarcerated, and families*. 2020, CDC.

26. Child Rights International Network, *Coronavirus and children in detention*. 2020.

27. Children of Prisoners Europe, *COVID-19: Call to action to protect vulnerable families and children in alternative care across Europe*. 2020.

28. Clarke, M., et al., *Establishing prison-led contact tracing to prevent outbreaks of COVID-19 in prisons in Ireland.* Journal of Public Health, 2020.

29. Commonwealth Human Rights Initiative, *COVID-19 and prisons in the commonwealth: Ensuring an effective response.* 2020.

30. US Centres for Disease Control and Prevention, *Interim Guidance on Management of Coronavirus Disease 2019 (COVID-19) in Correctional and Detention Facilities.* 2020.

31. Communicable Diseases Network Australia, *National Guidelines for the Prevention, Control and Public Health Management of COVID‐19 Outbreaks in Correctional and Detention Facilities in Australia.* 2020.

32. Council of Europe, *COVID-19 related statement by the members of the council for penological co-operation working group (PC-CP WG)*. 2020.

33. Council of Europe Commissioner for Human Rights, *Commissioner calls for release of immigration detainees during Covid-19 crisis*. 2020.

34. Council of Europe Commissioner for Human Rights, *COVID-19 pandemic: Urgent steps are needed to protect the rights of prisoners in Europe*. 2020.

35. Crowley, D., et al., *Prison and opportunities for the management of COVID-19.* BJGP Open, 2020.

36. Danish Institute Against Torture, *Reducing prison overcrowding in pre-trial detention and prison in the context of COVID-19* 2020.

37. Danish institute Against Torture, *Synthesis of Global guidance and recommendations on how to prevent and manage COVID-19 in prisons*. 2020.

38. Drogin, E.Y., *Forensic mental telehealth assessment (FMTA) in the context of COVID-19.* International Journal of Law and Psychiatry, 2020: p. 101595.

39. Emmanuel, R. and C. Hooper, *‘Sitting ducks’ For COVID-19: Detention or Death Trap?* AMSA Journal of Global Health, 2020. **14**(1): p. 48-51.

40. Emory Center for the Health of Incarcerated Persons, *Provisional Guidance on Management of COVID-19 in Jails, Prisons and Other Detention Settings.* 2020.

41. Europe Prison Litigation Network, *Appeal by European NGOs involved in the field of prison health and in the defence of the right to health protection for prisoners*. 2020.

42. European Centre for Disease Prevention and Control, *Guidance on infection prevention and control of coronavirus disease (COVID-19) in migrant and refugee reception and detention centres in the EU/EEA and the United Kingdom*. 2020.

43. European Centre for Disease Prevention and Control, *Infection prevention and control and surveillance for coronavirus disease (COVID-19) in prisons in EU/EEA countries and the UK*. 2020.

44. European Committee for the Prevention of Torture and Inhuman or Degrading Treatment or Punishment (CPT), *Statement of principles relating to the treatment of persons deprived of their liberty in the context of the coronavirus disease (COVID-19) pandemic*. 2020.

45. Executives Transforming Probation & Parole (EXiT), *Statement from community supervision executives on the importance of using best practices during the COVID-19 crisis*. 2020.

46. Fair and Just Prosecution, *Elected Prosecutors Call for Dramatic Reduction in Incarcerated and Detained Populations in Response to Coronavirus.* Federal Sentencing Reporter, 2020. **32**(4): p. 235-236.

47. FIACAT, *Joint Statement to governments of the Member States of the African Union and international human rights organisations in Africa: Faced with the spreading of COVID-19, take urgent and immediate measures to protect the rights of detainees in Africa*. 2020.

48. Franco-Paredes, C., et al., *COVID-19 in jails and prisons: A neglected infection in a marginalized population.* PLOS Neglected Tropical Diseases, 2020. **14**(6): p. e0008409.

49. Fróis, C., *COVID‐19 pandemic and social distancing in prisons* Anthropology Today, 2020. **36**(3): p. 25-26.

50. Gagnon, J.C., *The solitary confinement of incarcerated American youth during COVID-19.* Psychiatry Research, 2020. **291**: p. 113219.

51. Garcini, L.M., et al., *A tale of two crises: The compounded effect of COVID-19 and anti-immigration policy in the United States.* Psychol Trauma, 2020.

52. General Directorate “Execution of Sentences” Bulgaria, *Protocols for action in case of COVID-19 in places of deprivation of liberty* 2020.

53. Gorman, G. and M. Ramaswamy, *Detained during a pandemic: A postcard from the Midwest.* Public Health Nursing, 2020. **37**(3): p. 325-326.

54. Government of Canada Office of the Correctional Officer, *COVID-19 Status Update*. 2020.

55. Government of Canada Office of the Correctional Officer, *COVID-19 Update for Federal Corrections – June 19, 2020*. 2020.

56. Gulati, G., C.P. Dunne, and B.D. Kelly, *Prisons and the COVID-19 Pandemic.* Irish Journal of Psychological Medicine, 2020.

57. Hargreaves, S., et al., *Europe's migrant containment policies threaten the response to covid-19.* The BMJ, 2020. **368**: p. m1213.

58. Hawks, L., S. Woolhandler, and D. McCormick, *COVID-19 in prisons and jails in the United States.* JAMA Internal Medicine, 2020.

59. Henry, B.F., *Social Distancing and Incarceration: Policy and Management Strategies to Reduce COVID-19 Transmission and Promote Health Equity Through Decarceration.* Health Education & Behavior, 2020. **0**(0): p. 1090198120927318.

60. Hewson, T., et al., *Effects of the COVID-19 pandemic on the mental health of prisoners.* The Lancet Psychiatry, 2020. **7**(7): p. 568-570.

61. Inspectorate of Prisons for Scotland, *Alternative approach to scrutiny during the COVID-19 pandemic*. 2020.

62. HM Prison & Probation Service, *Prison transfers and remissions to and from mental health inpatient hospitals in realtion to COVID-19*. 2020, NHS England: United Kingdom.

63. Human Rights First. *COVID-19 and immigration detention*. 2020 17 March 2020 [cited 2020 6 July]; Available from: <https://www.humanrightsfirst.org/resource/covid-19-and-immigration-detention>.

64. Human Rights Law Centre. *Explainer: Prisons and COVID-19*. 2020 [cited 2020 2 July 2020]; Available from: <https://www.hrlc.org.au/prisons-and-covid19>.

65. Human Rights Watch, *Averting an Imminent Catastrophe: Recommendations to US Local, State and Federal Officials to Covid-19 in Jails and Prisons*. 2020.

66. Human Rights Watch, *COVID-19: A human rights checklist*. 2020.

67. Human Rights Watch, *Human Rights Dimensions of COVID-19 Response*. 2020.

68. Independent Advisory Panel on Deaths in Custody, *“Keep talking, stay safe”: A rapid review of prisoners’ experience under Covid-19*. 2020.

69. Innovative Prison Systems, *A Guide for Decision-Makers on Worldwide Practices and Recommendations*. 2020.

70. New South Wales Government, *Inspector of Custodial Services COVID-19 plan*. 2020.

71. Inter-Agency Standing Committee, *Interim guidance: COVID-19: Focus on persons deprived of their liberty*. 2020, IASC.

72. International Committee of the Red Cross (ICRC), *COVID-19 preparedness and response: Safeguarding the health of detainees, staff and communities*. 2020.

73. International Corrections and Prisons Association (ICPA), *Adapting to COVID-19: Prison Oversight and Monitoring During a Pandemic*. 2020.

74. International Detention Coalition. *IDC Position on Covid-19*. 2020; Available from: <https://idcoalition.org/covid-19/>.

75. International Drug Policy Consortium, *COVID-19: Prisons and detention in Southeast Asia*. 2020.

76. International Federation for Human Rights, *COVID-19: States bear direct responsibility for the health of individuals in their custody*. 2020.

77. International Federation for Human Rights, *Free female prisoners of conscience as coronavirus overtakes prisons*. 2020.

78. International Federation for Human Rights, *On Palestinian Prisoners’ Day, Civil Society Calls for Urgent Release of Palestinian Prisoners and Detainees in Israeli Prisons*. 2020.

79. International Federation for Human Rights, *Thailand - COVID-19: Release prisoners, ensure the health and safety of all those in detention facilities*. 2020.

80. Irvine, M., et al., *Modeling COVID-19 and Its Impacts on U.S. Immigration and Customs Enforcement (ICE) Detention Facilities, 2020.* Journal of urban health : bulletin of the New York Academy of Medicine, 2020.

81. Italian Department of Prison Administration, *Italy: Provisions to fight the COVID-19 contagion in prisons* 2020.

82. Johnson, S. and L. Beletsky, *Helping People Transition from Incarceration to Society During a Pandemic.* Northeastern University School of Law Research Paper, 2020(375-2020).

83. Justice and Corrections Service, *COVID-19 preparedness and response information package*. 2020, United Nations Department of Peace Operations.

84. Keller, A.S. and B.D. Wagner, *COVID-19 and immigration detention in the USA: time to act.* The Lancet Public Health, 2020. **5**(5): p. e245-e246.

85. Kinner, S.A., et al., *Prisons and custodial settings are part of a comprehensive response to COVID-19.* The Lancet Public Health, 2020. **5**(4): p. e188-e189.

86. Knox, C.M., *Managing Stressors During COVID-19.* Correctional Health Care Report, 2020. **21**(4): p. 53-62.

87. Kothari, R., et al., *COVID-19 and prisons: Providing mental health care for people in prison, minimising moral injury and psychological distress in mental health staff.* Medicine, Science and the Law, 2020. **0**(0): p. 0025802420929799.

88. Lachsz, A. and M. Hurley, *OPCAT, places of detention, and covid-19: Joint submission to the Select Committee on covid-19*. 2020.

89. León, M., K. Rodas, and M. Greer, *Leisure Behind Bars: The Realities of COVID-19 for Youth Connected to the Justice System.* Leisure Sciences, 2020: p. 1-7.

90. Liebrenz, M., et al., *Caring for persons in detention suffering with mental illness during the Covid-19 outbreak.* Forensic Science International: Mind and Law, 2020. **1**: p. 100013-100013.

91. Malta, G., S. Zerbo, and A. Argo, *The risk of the 'prison-emptying' effect in Italy due to the Covid-19 pandemic.* Med Leg J, 2020.

92. Manthorpe, M., *Statement by the Commonwealth Ombudsman Michael Manthorpe on the management of COVID-19 risks in immigration detention facilities*. 2020, Commonwealth Ombudsman: Australia.

93. Marcum, C.D., *American corrections system response to covid-19: An examination of the procedures and policies used in spring 2020.* American Journal of Criminal Justice, 2020: p. No-Specified.

94. Martyn, K., et al., *U.S. & Indiana county jail populations during the COVID-19 pandemic*. 2020, Center for Health and Justice Research, Public Policy Institute: Indianapolis, USA.

95. Mauer, M., *Letter on examining best practices for incarceration and detention during COVID-19*. 2020, The Sentencing Project: Washington, DC.

96. McKenzie, K.C. and R. Mishori, *Releasing Migrants from Detention During the Covid-19 Pandemic.* Journal of General Internal Medicine, 2020.

97. Meyer, J.P., et al., *COVID-19 and the coming epidemic in US immigration detention centres.* Lancet Infectious Diseases, 2020. **20**(6): p. 646-648.

98. Mijatovic, D. *Commissioner calls for release of immigration detainees while Covid-19 crisis continues*. 2020 26 March 2020 [cited 2020 3 July 2020]; Available from: <https://www.coe.int/en/web/commissioner/-/commissioner-calls-for-release-of-immigration-detainees-while-covid-19-crisis-continues>.

99. Minkler, M., J. Griffin, and P. Wakimoto, *Seizing the Moment: Policy Advocacy to End Mass Incarceration in the Time of COVID-19.* Health Education & Behavior, 2020. **0**(0): p. 1090198120933281.

100. Minnesota Department of Health, *Jails and correctional settings: Interim guidance for responding to cases of confirmed or suspected COVID-19*. 2020, Minnesota Department of Health: Minnesota, USA.

101. Montoya-Barthelemy, A.G., et al., *COVID-19 and the Correctional Environment: The American Prison as a Focal Point for Public Health.* American journal of preventive medicine, 2020. **58**(6): p. 888-891.

102. Mukherjee, T.I. and N. El-Bassel, *The perfect storm: COVID-19, mass incarceration and the opioid epidemic.* International Journal of Drug Policy, 2020: p. 102819.

103. National Aboriginal & Torres Strait Islander Legal Services, *NATSILS policy statement on COVID-19*. 2020, NATSILS: Australia.

104. National Aboriginal & Torres Strait Islander Legal Services (NATSILS), *Joint Statement: Aboriginal and Torres Strait Islander Legal Services call on the Prime Minister for early release from prison and other urgent measures to protect Aboriginal and Torres Strait Islander people from COVID-19 in the justice system*. 2020.

105. National Aboriginal & Torres Strait Islander Legal Services (NATSILS), *Media Release: NATSILS and the Australian Indigenous Doctors Association back calls for the early release of prisoners to prevent the spread of COVID-19*. 2020.

106. National Commission on Correctional Health Care. *COVID-19 Coronavirus: What You Need to Know in Corrections*. 2020 3 July 2020]; Available from: <https://www.ncchc.org/COVID-Resources>.

107. National Commission on Correctional Health Care, *Medical Isolation of COVID-19 Cases*. 2020.

108. National Commission on Correctional Health Care, *Prevention Practices for Corrections*. 2020.

109. National Commission on Correctional Health Care, *Prevention Practices of Inmate Population*. 2020.

110. National Commission on Correctional Health Care, *Social Distancing When Distance is a 8 X 12 Cell*. 2020.

111. National Council on Crime & Delinquency, *Community Supervision COVID-19 Guidance and Tips*. 2020.

112. National Juvenile Defender Center, *Guidance to juvenile courts on conducting remote hearings during the COVID-19 pandemic* 2020.

113. National Police Chief’s Council, *Guidance and Recommendations for the Provision of Police Custody during the COVID-19 Pandemic.* 2020.

114. Nature, *Tackle coronavirus in vulnerable communities.* Nature, 2020. **581**(7808): p. 239-240.

115. Njuguna, H., et al., *Serial Laboratory Testing for SARS-CoV-2 Infection Among Incarcerated and Detained Persons in a Correctional and Detention Facility — Louisiana, April–May 2020.* Morbidity and Mortality Weekly Report, 2020. **69**(26): p. 836-840.

116. Norwegian Institute of Public Health, *Advice to sectors working with people who may be infected with COVID-19* 2020.

117. Nowotny, K., et al., *COVID-19 Exposes Need for Progressive Criminal Justice Reform.* American Journal of Public Health, 2020. **110**(7): p. 967-968.

118. New Zealand Office of the Ombudsman, *Report on inspections of mental health facilities under the Crimes of Torture Act 1989*. 2020.

119. OHCHR, I., and the Palestinian Ministry of Interior,,, *Deprivation of liberty amid the outbreak of COVID-19: Joint note by OHCHR, ICHR, and the Palestinian Ministry of Interior* 2020.

120. Okano, J.T. and S. Blower, *Preventing major outbreaks of COVID-19 in jails*. 2020, Lancet: Philadelphia, Pennsylvania. p. 1542-1543.

121. Okonkwo, N.E., et al., *COVID-19 and the US response: accelerating health inequities.* BMJ evidence-based medicine, 2020.

122. O’Moore, É. and J. Farrar, *Briefing paper: Interim assessment of impact of various population management strategies in prisons in response to COVID-19 pandemic in England*. 2020.

123. Openshaw, J.J. and M.A. Travassos, *COVID-19 outbreaks in U.S. immigrant detention centers: the urgent need to adopt CDC guidelines for prevention and evaluation.* Clinical infectious diseases : an official publication of the Infectious Diseases Society of America, 2020.

124. Openshaw, J.J. and M.A. Travassos, *COVID-19, Quarantines, Sheltering-in-Place, and Human Rights: The Developing Crisis.* The American journal of tropical medicine and hygiene, 2020.

125. Orcutt, M., et al., *Global call to action for inclusion of migrants and refugees in the COVID-19 response.* The Lancet, 2020. **395**(10235): p. 1482-1483.

126. Page, K.R., et al., *Undocumented U.S. Immigrants and Covid-19.* N Engl J Med, 2020.

127. Pan American Health Organization, *Recommendations for the Cleaning and Disinfection of Sites Where People are Closely Confined and Deprived of Their Liberty: Penitentiaries, Prisons, and Migrant Detention Centers.* 2020.

128. Penal Reform International, *Coronavirus: Healthcare and human rights of people in prison*. 2020.

129. Penal Reform International, *Global Prison Trends 2020*. 2020.

130. Piel, J., *Letter to the Editor-Behavioral Health Implications of Inmate Release During COVID-19.* Journal of forensic sciences, 2020.

131. PRISON DEPARTMENT OF THE REPUBLIC OF LITHUANIA, *Regulations set in Lithuania in regards to the COVID-19*. 2020.

132. Protection International, *COVID-19: Release prisoners, ensure the health and safety of all those in detention facilities.* 2020.

133. Public Health England. *COVID-19: Prisons and other prescribed places of detention guidance*. 2020 [cited 2020 2 July 2020]; Available from: <https://www.gov.uk/government/publications/covid-19-prisons-and-other-prescribed-places-of-detention-guidance/covid-19-prisons-and-other-prescribed-places-of-detention-guidance>.

134. Public Health England, *Preventing and controlling outbreaks of COVID-19 in prisons and places of detention.* 2020.

135. Pyrooz, D.C., et al., *Views on COVID-19 from Inside Prison: Perspectives of High-security Prisoners.* Justice Evaluation Journal, 2020: p. 1-13.

136. Refugee Council of Australia. *Leaving no-one behind: Ensuring people seeking asylum and refugees and included in COVID-19 strategies*. 2020 [cited 2020 3 July 2020]; Available from: <https://www.refugeecouncil.org.au/priorities-covid-19/>.

137. Refugees International, *COVID-19 and the Displaced: Addressing the Threat of the Novel Coronavirus in Humanitarian Emergencies*

. 2020.

138. Reinhart, E. and D. Chen, *Incarceration And Its Disseminations: COVID-19 Pandemic Lessons From Chicago's Cook County Jail.* Health affairs (Project Hope), 2020.

139. Royal College of General Practitioners Secure Environments Group, *COVID-19 guidance for healthcare in secure environments: Practical advice for clinicians*. 2020.

140. Royal College of Psychiatrists. *COVID-19: Secure hospital and criminal justice settings*. 2020 [cited 2020 6 July 2020].

141. Rubenstein, L., *John Hopkins faculty letter on COVID-19 in jails and prisons*. 2020, John Hopkins Berman Institute of Bioethics: Baltimore.

142. Sanchez, A., et al., *COVID-19 in prisons: an impossible challenge for public health?* Cadernos de saude publica, 2020. **36**(5): p. e00083520.

143. Seal, D.W., *Impact of COVID-19 on Persons in Correctional Facilities–A Commentary.* Health Behavior and Policy Review, 2020. **7**(2): p. 161-164.

144. Shepherd, S. and B.L. Spivak, *Reconsidering the immediate release of prisoners during COVID-19 community restrictions.* The Medical journal of Australia, 2020.

145. Simpson, A.I., et al., *Management of COVID-19 Response in a Secure Forensic Mental Health Setting.* The Canadian Journal of Psychiatry, 2020: p. 0706743720935648.

146. Simpson, P.L. and T.G. Butler, *Covid-19, prison crowding, and release policies.* The BMJ, 2020. **369**: p. m1551.

147. Síndic El Defensor de les Personas, *Statement from the Catalan Ombudsman on the prevention of coronavirus spreading in prisons of Catalonia*. 2020.

148. Sivashanker, K., et al., *Covid-19 and decarceration: Healthcare needs to lead the charge.* Bmj-British Medical Journal, 2020. **369**: p. 2.

149. Special Rapporteur on Extrajudicial, S.o.A.K., ,, *COVID-19 and Protection of right to life in places of detention*. 2020.

150. Stewart, A., R. Cossar, and M. Stoové, *The response to COVID-19 in prisons must consider the broader mental health impacts for people in prison.* Australian & New Zealand Journal of Psychiatry, 2020. **0**(0): p. 0004867420937806.

151. Office of the United Nations High Commissioner for Human Rights (OCHCR), *Advice of the Subcommittee on Prevention of Torture to States Parties and National Preventive Mechanisms relating to the Coronavirus Pandemic (adopted on 25th March 2020)*. 2020.

152. Surprenant, C., *COVID-19 and pretrial detention*, in *Special Edition Policy Brief*. 2020.

153. Syrian Network for Human Rights, *The Most Notable Challenges for Medical Personnel, IDPs, Detainees and the Needy in Syria Amid the Spread of the COVID-19*. 2020.

154. Tahrir Institute for Middle East Policy (TIMEP) and Middle East and North Africa (MENA) Rights Group. *TIMEP Calls for Urgent Action around Detention in MENA Amid COVID-19*. 2020; Available from: <https://timep.org/press/press-releases/timep-calls-for-urgent-action-around-detention-in-mena-amid-covid-19/>.

155. Terres des hommes. *Accelerate release of children from detention: Protect children from COVID-19*. 2020 27 March 2020 [cited 2020 6 July 2020]; Available from: <https://www.tdh.ch/en/statements/release-children-detention-covid-19>.

156. The Alliance for Child Protection in Humanitarian Action, *Technical Note: COVID-19 and Children Deprived of their Liberty.* 2020.

157. The Bail Project, *The Bail Project Urges Jail Releases Amid Coronavirus Spread*. 2020.

158. The International Legal Foundation, *Coronavirus Pandemic: Guidance for Legal Aid Providers to Protect Health and Human Rights of Detainees*. 2020.

159. The Kirby Institute, *Report on COVID-19 and the impact on New South Wales prisoners*. 2020.

160. The UN Refugee Agency (UNHRC), *The rights and health of refugees, migrants and stateless must be protected in COVID-19 response: Joint press release from OHCHR, IOM, UNHCR and WHO.* 2020.

161. UN Women, *Justice for women amidst COVID-19*. 2020.

162. UNAIDS, *Rights in the time of COVID-19: Lessons from HIV for an effective, community-led response*. 2020.

163. UNICEF, *Children in detention are at heightened risk of contracting COVID-19 and should be released*. 2020.

164. UNICEF, *In light of COVID-19 crisis, UN officials call for immediate release of all children in detention, including Palestinian children*. 2020.

165. UNICEF, *Refoulement, detention, push-backs and deportations of children in the context of the COVID-19 pandemic* 2020.

166. United Nations, *COVID-19 and Human Rights: We are all in this together*. 2020.

167. UNODC, P.W., OCHCR Mexico,,, *Special standards UNAPS COVID-19*. 2020.

168. United Nations Human Rights Office of the High Commissioner, *COVID-19 Does Not Discriminate; Nor Should Our Response*. 2020.

169. United Nations Human Rights Office of the High Commissioner, *COVID-19: Americas / prison conditions*. 2020: Geneva.

170. United Nations Human Rights Office of the High Commissioner. *Urgent action needed to prevent COVID-19 "rampaging through paces of detention"- Bachelet*. 2020 25 March 2020 [cited 2020 2 July 2020]; Available from: <https://www.ohchr.org/EN/NewsEvents/Pages/DisplayNews.aspx?NewsID=25745&LangID=E>.

171. United Nations Human Rights Office of the High Commissioner (OHCHR), *COVID-19 and the human rights of migrants: Guidance*. 2020.

172. United Nations Human Rights Office of the High Commissioner (OHCHR), *US Government urged to do more to prevent major outbreaks of COVID-19 in detention centres – UN experts*. 2020.

173. United Nations Institute for Training and Research (UNITAR), *Operational toolbox: COVID-19 preparedness and response in places of detention* 2020.

174. United Nations Network on Migration, *COVID-19 & Immigration Detention: What Can Governments and Other Stakeholders Do?* 2020.

175. United Nations Network on Migration, *Forced returns of migrants must be suspended in times of COVID-19*. 2020.

176. United Nations Office on Drugs and Crime (UNODC), *COVID-19 prevention and control among people living in prison*. 2020.

177. United Nations Office on Drugs and Crime (UNODC), *COVID-19 prevention and control among people working in prison* 2020.

178. United Nations Office on Drugs and Crime (UNODC), *Ensuring Access to Justice in the Context of COVID-19*. 2020.

179. United Nations Office on Drugs and Crime (UNODC), *Position paper: COVID-19 preparedness and responses in prisons*. 2020.

180. United Nations Office on Drugs and Crime (UNODC), *Protecting children deprived of liberty during the COVID-19 outbreak*. 2020.

181. United Nations Office on Drugs and Crime (UNODC), *Viruses and places of detention*. 2020.

182. University of Oxford Faculty of Law, *Statement on healthcare provisions for undocumented migrants and those in immigration detention*. 2020.

183. Vera Institute of Justice, *Guidance for preventive and responsive measures to coronavirus for youth agencies*. 2020.

184. Vera Institute of Justice, *We must urgently do more to address COVID-19 behind bars and avoid mass infection and death: Guidance for Attorney General Barr, governors, shrits, and corrections administrators*. 2020, Vera Institute of Justice: New York, USA.

185. Vose, B., F.T. Cullen, and H. Lee, *Targeted Release in the COVID-19 Correctional Crisis: Using the RNR Model to Save Lives.* American Journal of Criminal Justice, 2020.

186. Wagner, P. and E. Widra, *Five ways the criminal justice system could slow the pandemic*. 2020, Prison Policy Initiative.

187. US Centres for Disease Control and Prevention, *COVID-19 in Correctional and Detention Facilities — United States, February–April 2020.* Morbidity and Mortality Weekly Report, 2020.

188. Wallace, M., et al., *COVID-19 in Correctional and Detention Facilities - United States, February-April 2020.* MMWR: Morbidity & Mortality Weekly Report, 2020. **69**(19): p. 587-590.

189. Waly, F.G., et al., *UNODC, WHO, UNAIDS, and OHCHR joint statement on COVID-19 in prisons and other closed settings*. 2020, WHO: Geneva.

190. Wang, J., et al., *Prevention and control of COVID-19 in nursing homes, orphanages, and prisons.* Environmental Pollution, 2020. **266**: p. 115161.

191. Office of the United Nations High Commissioner for Human Rights (OCHCR), *Deliberation No. 11 on prevention of arbitrary deprivation of liberty in the context of public health emergencies*. 2020.

192. World Health Organisation, *Checklist to evaluate preparedness, prevention and control of COVID-19 in prisons and other places of detention*. 2020.

193. World Health Organisation. *FAQ: Prevention and control of COVID-19 in prisons and other places of detention*. 2020; Available from: [https://www.euro.who.int/en/health-topics/health-emergencies/coronavirus-covid-19/technical-guidance/prevention-and-control-of-covid-19-in-prisons-and-other-places-of-detention/faq-prevention-and-control-of-covid-19-in-prisons-and-other-places-of-detention#](https://www.euro.who.int/en/health-topics/health-emergencies/coronavirus-covid-19/technical-guidance/prevention-and-control-of-covid-19-in-prisons-and-other-places-of-detention/faq-prevention-and-control-of-covid-19-in-prisons-and-other-places-of-detention).

194. World Health Organisation, *Preparedness, prevention and control of COVID-19 in prisons and other places of detention: Interim Guidance*. 2020.

195. World Organisation Against Torture, *Building our response on COVID-19 and detention: OMCT guidance brief to the SOS-Torture Network and partner organizations*. 2020, SOS-Torture Network: Geneva, Switzerland.

196. World Organisation Against Torture, *Dignity in the time of COVID-19*. 2020.

197. World Organisation Against Torture, *Philippines: Immediately release Filipino political prisoners and end detention and ill-treatment of curfew violators*. 2020.

198. World Organisation Against Torture, *Turkey: Grant early release from overcrowded prisons without discrimination.* 2020.

199. Wurcel, A.G., et al., *Spotlight on Jails: COVID-19 Mitigation Policies Needed Now.* Clinical Infectious Diseases, 2020.

200. Yang, H. and J.R. Thompson, *Fighting covid-19 outbreaks in prisons.* BMJ, 2020. **369**: p. m1362.

201. Youth Correctional Leaders for Justice, *Recommendations for Youth Justice Systems During the COVID-19 Emergency*. 2020.
