# Supplementary material for: A rapid review of early guidance to prevent and control COVID-19 in custodial settings
Source: Health Justice. 2021 Oct 15;9:27. doi: 10.1186/s40352-021-00150-w (PMC8518275; doi:10.1186/s40352-021-00150-w)
Supplement: Supplementary file 3 — Additional file 3: Appendix S3. Search strategy; Description of data: Appendix S3 provides the comprehensive search strategy to allow for reproduction of the search results. [file 40352_2021_150_MOESM3_ESM.docx]

**Appendix S3** Search strategy

Medline(Ovid)

| # | Search | Results |
| --- | --- | --- |
| 1 | (prison* or incarcerat* or decarcer* or custod* or imprison* or internment or detention* or detain* or sentenc* or inmate* or jail* or penitentiary or gaol* or correctional* or probation* or parole* or remand* or offend* or convic* or felon* or criminal*).mp. | 106575 |
| 2 | ((pre or under or await*) adj2 trial).mp. | 2575 |
| 3 | (depriv* adj2 (liberty or freedom)).mp. | 224 |
| 4 | (solitary adj2 confine*).mp. | 144 |
| 5 | (secure* adj2 (facilit* or center* or centre* or complex*)).mp. | 261 |
| 6 | (holding adj2 (facilit* or center* or centre* or complex*)).mp. | 220 |
| 7 | (punitive adj2 (system* or setting* or centre* or complex* or facilit* or service* or unit* or environment*)).mp. | 84 |
| 8 | (correction* adj2 (system* or setting* or centre* or complex* or facilit* or service* or unit* or environment*)).mp | 3917 |
| 9 | (closed adj2 (setting or environment* or facilit* or unit* or camp* or institut*)).mp. | 1188 |
| 10 | (legal adj2 (system* or setting* or facilit* or involve* or service*)).mp. | 3249 |
| 11 | (penal adj2 (system* or setting* or centre* or center* or complex* or facilit* or service* or unit* or environment*)).mp. | 152 |
| 12 | (reform*-school* or delinquent).mp. | 3166 |
| 13 | (justice adj2 (youth* or juvenile* or adolescen* or system* or setting*)).mp. | 4137 |
| 14 | (forensic adj2 (psych* or order* or patient* or inpatient* or setting* or centre* or center* or containment* or facilit* or unit*)).mp. | 11801 |
| 15 | Forensic Psychiatry/ | 9044 |
| 16 | Prisons/ | 9646 |
| 17 | Prisoners/ | 16541 |
| 18 | Criminals/ | 4884 |
| 19 | or/1-18 | 124537 |
| 20 | (migrant* or refugee* or migrat* or immigrant* or emigrant* or emigrat* or transient* or asylum* or immigrat*).mp. | 732657 |
| 21 | "Emigration and Immigration"/ | 25134 |
| 22 | Refugees/ or "Transients and Migrants"/ | 20807 |
| 23 | or/20-22 | 732657 |
| 24 | (containment adj3 (center* or centre* or facilit* or complex* or policy or policies or authorit*)).mp. | 507 |
| 25 | (holding adj3 (center* or centre* or facilit* or complex* or policy or policies or authorit*)).mp. | 333 |
| 26 | (provisional adj3 (center* or centre* or facilit* or complex* or policy or policies or authorit*)).mp. | 64 |
| 27 | (deportation adj3 (center* or centre* or facilit* or complex* or policy or policies or authorit*)).mp. | 17 |
| 28 | (removal adj3 (center* or centre* or facilit* or complex* or policy or policies or authorit*)).mp. | 3153 |
| 29 | (ICE adj3 (enforcement* or operation* or center* or centre* or facilit* or complex* or policy or policies or authorit*)).mp. | 314 |
| 30 | or/24-29 | 4383 |
| 31 | 30 and 23 | 204 |
| 32 | 31 or 19 | 124688 |
| 33 | limit 32 to covid-19 | 126 |

PsycINFO

| # | Search | Results |
| --- | --- | --- |
| 1 | (prison* or incarcerat* or decarcer* or custod* or imprison* or internment or detention* or detain* or sentenc* or inmate* or jail* or penitentiary or gaol* or correctional* or probation* or parole* or remand* or offend* or convic* or felon* or criminal*).mp. | 159304 |
| 2 | ((pre or under or await*) adj2 trial).mp. | 820 |
| 3 | (depriv* adj2 (liberty or freedom)).mp. | 175 |
| 4 | (solitary adj2 confine*).mp. | 237 |
| 5 | (secure* adj2 (facilit* or center* or centre* or complex*)).mp. | 336 |
| 6 | (holding adj2 (facilit* or center* or centre* or complex*)).mp. | 70 |
| 7 | (punitive adj2 (system* or setting* or centre* or complex* or facilit* or service* or unit* or environment*)).mp. | 98 |
| 8 | (correction* adj2 (system* or setting* or centre* or complex* or facilit* or service* or unit* or environment*)).mp | 4239 |
| 9 | (closed adj2 (setting or environment* or facilit* or unit* or camp* or institut*)).mp. | 363 |
| 10 | (legal adj2 (system* or setting* or facilit* or involve* or service*)).mp. | 5225 |
| 11 | (penal adj2 (system* or setting* or centre* or center* or complex* or facilit* or service* or unit* or environment*)).mp. | 478 |
| 12 | (reform*-school* or delinquent).mp. | 10797 |
| 13 | (justice adj2 (youth* or juvenile* or adolescen* or system* or setting*)).mp. | 12639 |
| 14 | (forensic adj2 (psych* or order* or patient* or inpatient* or setting* or centre* or center* or containment* or facilit* or unit*)).mp. | 13208 |
| 15 | Forensic Psychiatry/ or Forensic Psychology/ | 9018 |
| 16 | exp Correctional Institutions/ or Incarceration/ | 13960 |
| 17 | Prisoners/ | 10704 |
| 18 | exp Criminal offenders/ | 19955 |
| 19 | exp Criminal Justice/ | 12965 |
| 20 | or/1-19 | 179429 |
| 21 | (migrant* or refugee* or migrat* or immigrant* or emigrant* or emigrat* or transient* or asylum* or immigrat*).mp. | 93466 |
| 22 | Immigration/ or Asylum Seeking/ | 22880 |
| 23 | Refugees/ | 6142 |
| 24 | or/21-23 | 93466 |
| 25 | 24 or 20 | 269403 |
| 26 | (COVID19* or COVID-19* or SARS-CoV* or 2019-novel-coronavirus* or 2019-novel-corona-virus* or 2019-nCOV or 2019nCOV or coronavirus-disease* or corona-virus-disease or ncovid* or new-coronavirus or new-corona-virus or novel-coronavirus or novel-corona-virus or ncov* or covid* or sars-coronavirus* or sars-corona-virus* or 2019-novel-cov* or sarscov*).mp. | 541 |
| 27 | 25 and 26 | 18 |

Embase

| # | Search | Records |
| --- | --- | --- |
| 1 | (prison* or incarcerat* or decarcer* or custod* or imprison* or internment or detention* or detain* or sentenc* or inmate* or jail* or penitentiary or gaol* or correctional* or probation* or parole* or remand* or offend* or convic* or felon* or criminal*).mp. | 154023 |
| 2 | ((pre or under or await*) adj2 trial).mp. | 4162 |
| 3 | (depriv* adj2 (liberty or freedom)).mp. | 290 |
| 4 | (solitary adj2 confine*).mp. | 173 |
| 5 | (secure* adj2 (facilit* or center* or centre* or complex*)).mp. | 390 |
| 6 | (holding adj2 (facilit* or center* or centre* or complex*)).mp. | 272 |
| 7 | (punitive adj2 (system* or setting* or centre* or complex* or facilit* or service* or unit* or environment*)).mp. | 104 |
| 8 | (correction* adj2 (system* or setting* or centre* or complex* or facilit* or service* or unit* or environment*)).mp | 4780 |
| 9 | (closed adj2 (setting or environment* or facilit* or unit* or camp* or institut*)).mp. | 1663 |
| 10 | (legal adj2 (system* or setting* or facilit* or involve* or service*)).mp. | 4632 |
| 11 | (penal adj2 (system* or setting* or centre* or center* or complex* or facilit* or service* or unit* or environment*)).mp. | 224 |
| 12 | (reform*-school* or delinquent).mp. | 4164 |
| 13 | (justice adj2 (youth* or juvenile* or adolescen* or system* or setting*)).mp. | 5152 |
| 14 | (forensic adj2 (psych* or order* or patient* or inpatient* or setting* or centre* or center* or containment* or facilit* or unit*)).mp. | 17560 |
| 15 | Forensic Psychiatry/ or Forensic psychology/ | 13688 |
| 16 | exp Prison/ or Detention/ or Criminal Justice/ | 24846 |
| 17 | Prisoner/ | 17704 |
| 18 | Offender/ | 15211 |
| 19 | or/1-18 | 179913 |
| 20 | (migrant* or refugee* or migrat* or immigrant* or emigrant* or emigrat* or transient* or asylum* or immigrat*).mp. | 993596 |
| 21 | exp Migrant/ | 38010 |
| 22 | or/20-21 | 993596 |
| 23 | (containment adj3 (center* or centre* or facilit* or complex* or policy or policies or authorit*)).mp. | 625 |
| 24 | (holding adj3 (center* or centre* or facilit* or complex* or policy or policies or authorit*)).mp. | 409 |
| 25 | (provisional adj3 (center* or centre* or facilit* or complex* or policy or policies or authorit*)).mp. | 103 |
| 26 | (deportation adj3 (center* or centre* or facilit* or complex* or policy or policies or authorit*)).mp. | 14 |
| 27 | (removal adj3 (center* or centre* or facilit* or complex* or policy or policies or authorit*)).mp. | 4151 |
| 28 | (ICE adj3 (enforcement* or operation* or center* or centre* or facilit* or complex* or policy or policies or authorit*)).mp. | 360 |
| 29 | or/23-28 | 5656 |
| 30 | 29 and 22 | 268 |
| 31 | 30 or 19 | 180119 |
| 32 | limit 31 to covid-19 | 143 |

Web of Science

| # | Search | Records |
| --- | --- | --- |
| 1 | TS=(prison* or incarcerat* or decarcer* or custod* or imprison* or internment or detention* or detain* or sentenc* or inmate* or jail* or penitentiary or gaol* or correctional* or probation* or parole* or remand* or offend* or convic* or felon* or criminal*) | 265237 |
| 2 | TS=((pre or under or await*) NEAR/2 trial) | 9166 |
| 3 | TS=(depriv* NEAR/2 (liberty or freedom)) | 586 |
| 4 | TS=(solitary NEAR/2 confine*) | 402 |
| 5 | TS=(secure* NEAR/2 (facilit* or center* or centre* or complex*)) | 1237 |
| 6 | TS=(holding NEAR/2 (facilit* or center* or centre* or complex*)) | 3004 |
| 7 | TS=(punitive NEAR/2 (system* or setting* or centre* or complex* or facilit* or service* or unit* or environment*)) | 245 |
| 8 | TS=(correction* NEAR/2 (system* or setting* or centre* or complex* or facilit* or service* or unit* or environment*)) | 11178 |
| 9 | TS=(closed NEAR/2 (setting or environment* or facilit* or unit* or camp* or institut*)) | 22992 |
| 10 | TS=(legal NEAR/2 (system* or setting* or facilit* or involve* or service*)) | 16672 |
| 11 | TS=(penal NEAR/2 (system* or setting* or centre* or center* or complex* or facilit* or service* or unit* or environment*)) | 914 |
| 12 | TS=(reform*-school* or delinquent) | 9881 |
| 13 | TS=(justice NEAR/2 (youth* or juvenile* or adolescen* or system* or setting*)) | 13804 |
| 14 | TS=(forensic NEAR/2 (psych* or order* or patient* or inpatient* or setting* or centre* or center* or containment* or facilit* or unit*)) | 8127 |
| 15 | #14 OR #13 OR #12 OR #11 OR #10 OR #9 OR #8 OR #7 OR #6 OR #5 OR #4 OR #3 OR #2 OR #1 | 338397 |
| 16 | TS=(migrant* or refugee* or migrat* or immigrant* or emigrant* or emigrat* or transient* or asylum* or immigrat*) | 1346442 |
| 17 | TS=(containment NEAR/3 (center* or centre* or facilit* or complex* or policy or policies or authorit*)) | 1169 |
| 18 | TS=(holding NEAR/3 (center* or centre* or facilit* or complex* or policy or policies or authorit*)) | 6160 |
| 19 | TS=(provisional NEAR/3 (center* or centre* or facilit* or complex* or policy or policies or authorit*)) | 203 |
| 20 | TS=(deportation NEAR/3 (center* or centre* or facilit* or complex* or policy or policies or authorit*)) | 143 |
| 21 | TS=(removal NEAR/3 (center* or centre* or facilit* or complex* or policy or policies or authorit*)) | 6250 |
| 22 | TS=(ICE NEAR/3 (enforcement* or operation* or center* or centre* or facilit* or complex* or policy or policies or authorit*)) | 3268 |
| 23 | #22 or #21 or #20 or #19 or #18 or #17 | 17162 |
| 24 | #16 and #23 | 834 |
| 25 | #15 or #24 | 338980 |
| 26 | TS=(COVID19* or COVID-19* or SARS-CoV* or 2019-novel-coronavirus* or 2019-novel-corona-virus* or 2019-nCOV or 2019nCOV or coronavirus-disease* or corona-virus-disease or ncovid* or new-coronavirus or new-corona-virus or novel-coronavirus or novel-corona-virus or ncov* or covid* or sars-coronavirus* or sars-corona-virus* or 2019-novel-cov* or sarscov*) | 16436 |
| 27 | #25 and #26 | 92 |

CINAHL

| # | Search | Records |
| --- | --- | --- |
| 1 | TI (prison* or incarcerat* or decarcer* or custod* or imprison* or internment or detention* or detain* or sentenc* or inmate* or jail* or penitentiary or gaol* or correctional* or probation* or parole* or remand* or offend* or convic* or felon* or criminal*) or AB (prison* or incarcerat* or decarcer* or custod* or imprison* or internment or detention* or detain* or sentenc* or inmate* or jail* or penitentiary or gaol* or correctional* or probation* or parole* or remand* or offend* or convic* or felon* or criminal*) | 46288 |
| 2 | TX ((pre or under or await*) n2 trial) | 8388 |
| 3 | TX (depriv* n2 (liberty or freedom)) | 1038 |
| 4 | TX (solitary n2 confine*) | 326 |
| 5 | TX (secure* n2 (facilit* or center* or centre* or complex*)) | 1535 |
| 6 | TX (holding n2 (facilit* or center* or centre* or complex*)) | 566 |
| 7 | TX (punitive n2 (system* or setting* or centre* or complex* or facilit* or service* or unit* or environment*)) | 483 |
| 8 | TX(correction* n2 (system* or setting* or centre* or complex* or facilit* or service* or unit* or environment*)) | 13550 |
| 9 | TX(closed n2 (setting or environment* or facilit* or unit* or camp* or institut*)) | 3095 |
| 10 | TX (legal n2 (system* or setting* or facilit* or involve* or service*)) | 13080 |
| 11 | TX (penal n2 (system* or setting* or centre* or center* or complex* or facilit* or service* or unit* or environment*)) | 381 |
| 12 | TX (reform*-school* or delinquent) | 6746 |
| 13 | TX (justice n2 (youth* or juvenile* or adolescen* or system* or setting*)) | 13172 |
| 14 | TX (forensic n2 (psych* or order* or patient* or inpatient* or setting* or centre* or center* or containment* or facilit* or unit*)) | 10210 |
| 15 | MH "Forensic Psychiatry" | 1940 |
| 16 | (MH "Correctional Facilities") or (MH "Correctional Health Services") | 7659 |
| 17 | (MH "Public Offenders+") or (MH "Prisoners") | 20702 |
| 18 | (MH "Mentally Ill Offenders") | 2211 |
| 19 | S1 or S2 or S3 or S4 or S5 or S6 or S7 or S8 or S9 or S10 or S11 or S12 or S13 or S14 or S15 or S16 or S17 or S18 | 101183 |
| 20 | TX (migrant* or refugee* or migrat* or immigrant* or emigrant* or emigrat* or transient* or asylum* or immigrat*) | 216621 |
| 21 | (MH "Immigrants+") | 1752 |
| 22 | (MH "Refugees") | 7779 |
| 23 | S20 or S21 or S22 | 216612 |
| 24 | TX (containment n3 (center* or centre* or facilit* or complex* or policy or policies or authorit*)) | 921 |
| 25 | TX (holding n3 (center* or centre* or facilit* or complex* or policy or policies or authorit*)) | 1109 |
| 26 | TX (provisional n3 (center* or centre* or facilit* or complex* or policy or policies or authorit*)) | 184 |
| 27 | TX (deportation n3 (center* or centre* or facilit* or complex* or policy or policies or authorit*)) | 96 |
| 28 | TX (removal n3 (center* or centre* or facilit* or complex* or policy or policies or authorit*)) | 2272 |
| 29 | TX (ICE n3 (enforcement* or operation* or center* or centre* or facilit* or complex* or policy or policies or authorit*)) | 877 |
| 30 | S24 or S25 or S26 or S27 or S28 or S29 | 5388 |
| 31 | S30 and S23 | 1070 |
| 32 | S31 or S19 | 101971 |
| 33 | TX (COVID19* or COVID-19* or SARS-CoV* or 2019-novel-coronavirus* or 2019-novel-corona-virus* or 2019-nCOV or 2019nCOV or coronavirus-disease* or corona-virus-disease or ncovid* or new-coronavirus or new-corona-virus or novel-coronavirus or novel-corona-virus or ncov* or covid* or sars-coronavirus* or sars-corona-virus* or 2019-novel-cov* or sarscov*) | 12198 |
| 34 | (MH "Coronavirus infections") | 2388 |
| 35 | (MH "Coronavirus") | 508 |
| 36 | S33 or S34 or S35 | 13016 |
| 37 | S32 and S36 | 211 |

Global Health (CABI)

| # | Search | Records |
| --- | --- | --- |
| 1 | title:((prison* or incarcerat* or decarcer* or custod* or imprison* or internment or detention* or detain* or sentenc* or inmate* or jail* or penitentiary or gaol* or correctional* or probation* or parole* or remand* or offend* or convic* or felon* or criminal*)) OR ab:((prison* or incarcerat* or decarcer* or custod* or imprison* or internment or detention* or detain* or sentenc* or inmate* or jail* or penitentiary or gaol* or correctional* or probation* or parole* or remand* or offend* or convic* or felon* or criminal*)) | 21041 |
| 2 | title:(((pre or under or await*) NEAR/2 trial)) OR ab:(((pre or under or await*) NEAR/2 trial)) | 65339 |
| 3 | title:((depriv* NEAR/2 (liberty or freedom))) OR ab:((depriv* NEAR/2 (liberty or freedom))) | 113 |
| 4 | title:((solitary NEAR/2 confine*)) OR ab:((solitary NEAR/2 confine*)) | 108 |
| 5 | title:((secure* NEAR/2 (facilit* or center* or centre* or complex*))) OR ab:((secure* NEAR/2 (facilit* or center* or centre* or complex*))) | 2392 |
| 6 | title:((holding NEAR/2 (facilit* or center* or centre* or complex*))) OR ab:((holding NEAR/2 (facilit* or center* or centre* or complex*))) | 15870 |
| 7 | title:((punitive NEAR/2 (system* or setting* or centre* or complex* or facilit* or service* or unit* or environment*))) OR ab:((punitive NEAR/2 (system* or setting* or centre* or complex* or facilit* or service* or unit* or environment*))) | 219 |
| 8 | title:((correction* NEAR/2 (system* or setting* or centre* or complex* or facilit* or service* or unit* or environment*)) OR ab:((correction* n2 (system* or setting* or centre* or complex* or facilit* or service* or unit* or environment*)) | 426 |
| 9 | title:((closed NEAR/2 (setting or environment* or facilit* or unit* or camp* or institut*))) OR ab:((closed NEAR/2 (setting or environment* or facilit* or unit* or camp* or institut*))) | 60978 |
| 10 | title:((legal NEAR/2 (system* or setting* or facilit* or involve* or service*))) OR ab:((legal NEAR/2 (system* or setting* or facilit* or involve* or service*))) | 14700 |
| 11 | title:((penal NEAR/2 (system* or setting* or centre* or center* or complex* or facilit* or service* or unit* or environment*))) OR ab:((penal NEAR/2 (system* or setting* or centre* or center* or complex* or facilit* or service* or unit* or environment*))) | 160 |
| 12 | title:((reform*-school* or delinquent)) OR ab:((reform*-school* or delinquent)) | 560 |
| 13 | title:((justice NEAR/2 (youth* or juvenile* or adolescen* or system* or setting*))) OR ab:((justice NEAR/2 (youth* or juvenile* or adolescen* or system* or setting*))) | 2250 |
| 14 | title:((forensic NEAR/2 (psych* or order* or patient* or inpatient* or setting* or centre* or center* or containment* or facilit* or unit*))) OR ab:((forensic NEAR/2 (psych* or order* or patient* or inpatient* or setting* or centre* or center* or containment* or facilit* or unit*))) | 1159 |
| 15 | subject: ("Correctional institutions") | 3039 |
| 16 | subject: ("Prisoners") | 4363 |
| 17 | or/1-16 | 84378 |
| 18 | title:((migrant* or refugee* or migrat* or immigrant* or emigrant* or emigrat* or transient* or asylum* or immigrat*)) OR ab:((migrant* or refugee* or migrat* or immigrant* or emigrant* or emigrat* or transient* or asylum* or immigrat*)) | 188904 |
| 19 | subject: ("Immigration" or "Immigrants") | 9514 |
| 20 | subject: ("Refugees") | 4702 |
| 21 | or/18-20 | 189745 |
| 22 | title:((containment NEAR/3 (center* or centre* or facilit* or complex* or policy or policies or authorit*))) OR ab:((containment NEAR/3 (center* or centre* or facilit* or complex* or policy or policies or authorit*))) | 1441 |
| 23 | title:((holding NEAR/3 (center* or centre* or facilit* or complex* or policy or policies or authorit*))) OR ab:((holding NEAR/3 (center* or centre* or facilit* or complex* or policy or policies or authorit*))) | 23788 |
| 24 | title:((provisional NEAR/3 (center* or centre* or facilit* or complex* or policy or policies or authorit*))) OR ab:((provisional NEAR/3 (center* or centre* or facilit* or complex* or policy or policies or authorit*))) | 762 |
| 25 | title:((deportation NEAR/3 (center* or centre* or facilit* or complex* or policy or policies or authorit*))) OR ab:((deportation NEAR/3 (center* or centre* or facilit* or complex* or policy or policies or authorit*))) | 70 |
| 26 | title:((removal NEAR/3 (center* or centre* or facilit* or complex* or policy or policies or authorit*))) OR ab:((removal NEAR/3 (center* or centre* or facilit* or complex* or policy or policies or authorit*))) | 19723 |
| 27 | title:((ICE NEAR/3 (enforcement* or operation* or center* or centre* or facilit* or complex* or policy or policies or authorit*))) OR ab:((ICE NEAR/3 (enforcement* or operation* or center* or centre* or facilit* or complex* or policy or policies or authorit*))) | 4335 |
| 28 | or/22-27 | 49416 |
| 29 | 28 and 21 | 1457 |
| 30 | 29 or 17 | 85715 |
| 31 | title:((COVID19* or COVID-19* or SARS-CoV* or 2019-novel-coronavirus* or 2019-novel-corona-virus* or 2019-nCOV or 2019nCOV or coronavirus-disease* or corona-virus-disease or ncovid* or new-coronavirus or new-corona-virus or novel-coronavirus or novel-corona-virus or ncov* or covid* or sars-coronavirus* or sars-corona-virus* or 2019-novel-cov* or sarscov*)) OR ab:((COVID19* or COVID-19* or SARS-CoV* or 2019-novel-coronavirus* or 2019-novel-corona-virus* or 2019-nCOV or 2019nCOV or coronavirus-disease* or corona-virus-disease or ncovid* or new-coronavirus or new-corona-virus or novel-coronavirus or novel-corona-virus or ncov* or covid* or sars-coronavirus* or sars-corona-virus* or 2019-novel-cov* or sarscov*)) | 2788 |
| 32 | subject: ("Coronavirus") | 5498 |
| 33 | 31 or 32 | 6978 |
| 34 | 33 and 30 | 85 |

Criminal Justice Abstracts

| 1 | TX (COVID19* or COVID-19* or SARS-CoV* or 2019-novel-coronavirus* or 2019-novel-corona-virus* or 2019-nCOV or 2019nCOV or coronavirus-disease* or corona-virus-disease or ncovid* or new-coronavirus or new-corona-virus or novel-coronavirus or novel-corona-virus or ncov* or covid* or sars-coronavirus* or sars-corona-virus* or 2019-novel-cov* or sarscov*) | 278 |
| --- | --- | --- |

LILACS

| # | Search | Results |
| --- | --- | --- |
| 1 | (tw:(prison* or incarcerat* or decarcer* or custod* or imprison* or internment or detention* or detain* or sentenc* or inmate* or jail* or penitentiary or gaol* or correctional* or corrections or probation* or parole* or remand* or offend* or convic* or felon* or criminal* or migrant* or refugee* or migrat* or immigrant* or imigrat* or asylum* or "closed ward" or "closed unit" or "forensic psychiatry")) AND (tw:(COVID19* or COVID-19* or SARS-CoV* or 2019-novel-coronavirus* or 2019-novel-corona-virus* or 2019-nCOV or 2019nCOV or coronavirus-disease* or corona-virus-disease or ncovid* or new-coronavirus or new-corona-virus or novel-coronavirus or novel-corona-virus or ncov* or covid* or sars-coronavirus* or sars-corona-virus* or 2019-novel-cov* or sarscov*)) | 716 |
| 2 | (tw:(prison* or incarcerat* or decarcer* or custod* or imprison* or internment or detention* or detain* or sentenc* or inmate* or jail* or penitentiary or gaol* or correctional* or corrections or probation* or parole* or remand* or offend* or convic* or felon* or criminal* or migrant* or refugee* or migrat* or immigrant* or imigrat* or asylum* or "closed ward" or "closed unit" or "forensic psychiatry")) AND (tw:(COVID19* or COVID-19* or SARS-CoV* or 2019-novel-coronavirus* or 2019-novel-corona-virus* or 2019-nCOV or 2019nCOV or coronavirus-disease* or corona-virus-disease or ncovid* or new-coronavirus or new-corona-virus or novel-coronavirus or novel-corona-virus or ncov* or covid* or sars-coronavirus* or sars-corona-virus* or 2019-novel-cov* or sarscov*)) | 84 |

LitCovid

| # | Search | Records |
| --- | --- | --- |
| 1 | prison or incarcerate or decarcerate or custody or imprison or detention or detain or sentence or inmate or jail or penitentiary or gaol or probation or parole or remand or offend or convict or felon or criminal or immigrant or refugee or asylum or "correctional facility" or "forensic psychiatry" or migrant or "closed unit" or "closed ward" | 236 |

Google Scholar

| # | Search | Results |
| --- | --- | --- |
| 1 | (prison OR incarceration OR decarceration OR detention OR detain OR inmate OR parole OR "correctional facility" OR "forensic psychiatry" OR "closed unit" OR "closed ward") AND (COVID OR coronavirus OR sars-cov) | 5280 |
| Note: Limited search to publications since 2020. First 30 pages of search results were scanned for relevant publications. | | |

WorldWideScience

| # | Search | Results |
| --- | --- | --- |
| 1 | (COVID* OR "SARS-CoV" OR "coronavirus" OR ncov* OR "2019nCoV" OR "Corona virus" OR "Sars-coronavirus" OR Sarscov*) AND (prison* OR Detention* OR Inmate* OR Jail* OR "Penitentiary" OR "secure facility" OR "secure center" OR "secure centre" OR "secure complex" OR forensic psych* OR forensic order* OR forensic patient* OR forensic inpatient* OR forensic setting* OR forensic centre* OR forensic center* or forensic containment* OR forensic facilit* OR forensic unit*) | 872 |
| 2 | 1, restricting to year 2020 and English language only | 229 |
| 3 | 2, excluding PubMed Central and Europe PubMed | 88 |
| 4 | (COVID* OR "SARS-CoV" OR "coronavirus" OR ncov* OR "2019nCoV" OR "Corona virus" OR "Sars-coronavirus" OR Sarscov*) AND (prison* OR Detention* OR Inmate* OR Jail* OR "Penitentiary" OR "secure facility" OR "secure center" OR "secure centre" OR "secure complex" OR forensic psych* OR forensic order* OR forensic patient* OR forensic inpatient* OR forensic setting* OR forensic centre* OR forensic center* or forensic containment* OR forensic facilit* OR forensic unit*) AND ("policy" OR "policies" OR "guidance" OR "recommend" OR "guideline" OR "position" OR "measure" OR "statement" OR "announce" OR "letter" OR "monitor" OR "advice" OR "control" OR "measure" OR "statement" OR "note" OR "press release" OR "practice" OR "consideration" OR "update" OR "declar" OR "call" OR "plan" OR "manage" OR "report" OR "prevent") | 837 |
| 5 | 4, restricting to year 2020 and English language only | 179 |
| 6 | 5, excluding PubMed Central and Europe PubMed | 68 |

TRIP Database

| # | Search | Results |
| --- | --- | --- |
| 1 | (COVID* OR "SARS-CoV" OR "coronavirus" OR ncov* OR "2019nCoV" OR "Corona virus" OR "Sars-coronavirus" OR Sarscov*) AND (prison* OR Detention* OR Inmate* OR Jail* OR "Penitentiary" OR "secure facility" OR "secure center" OR "secure centre" OR "secure complex" OR forensic psych* OR forensic order* OR forensic patient* OR forensic inpatient* OR forensic setting* OR forensic centre* OR forensic center* or forensic containment* OR forensic facilit* OR forensic unit*) | 51 |
| 2 | (COVID* OR "SARS-CoV" OR "coronavirus" OR ncov* OR "2019nCoV" OR "Corona virus" OR "Sars-coronavirus" OR Sarscov*) AND (prison* OR Detention* OR Inmate* OR Jail* OR "Penitentiary" OR "secure facility" OR "secure center" OR "secure centre" OR "secure complex" OR forensic psych* OR forensic order* OR forensic patient* OR forensic inpatient* OR forensic setting* OR forensic centre* OR forensic center* or forensic containment* OR forensic facilit* OR forensic unit*) AND ("policy" OR "policies" OR "guidance" OR "recommend" OR "guideline" OR "position" OR "measure" OR "statement" OR "announce" OR "letter" OR "monitor" OR "advice" OR "control" OR "measure" OR "statement" OR "note" OR "press release" OR "practice" OR "consideration" OR "update" OR "declar" OR "call" OR "plan" OR "manage" OR "report" OR "prevent") | 51 |

Google Search Engine

| # | Search | Results |
| --- | --- | --- |
| 1 | (covid OR sars-cov OR coronavirus) AND (prison OR detention OR jail OR "secure facility" OR parole OR incarceration OR "forensic psychiatry") AND (policy OR guidance OR recommend OR "guideline" OR "measure" OR "statement" OR "announce" OR "letter" OR "advice" OR "control" OR "measure" OR "statement" OR "press release" OR "practice" OR "consideration" OR "manage" OR "report" OR "prevent") | 998M |
| Note: Limited search to publications since 2020. First 30 pages of search results were scanned for relevant publications. | | |

**Additional Grey Literature Searches (manual search)**

Targeted organisations for manual ‘site’ searches

| **Targeted organisation name** | **Web address** |
| --- | --- |
| Association for the Prevention of Torture (APT) | <https://www.apt.ch/en> |
| Amnesty International | <https://www.amnesty.org/en/> |
| CPT – European Committee for the Prevention of Torture and Inhuman or Degrading Treatment or Punishment (CPT) | <https://www.coe.int/en/web/cpt> |
| Human Rights Watch (HRW) | <https://www.hrw.org> |
| Inter-Agency Standing Committee (IASC – UN) | [https://interagencystandingcommittee.org](https://interagencystandingcommittee.org/) |
| International Corrections and Prisons Association (ICPA) | <https://icpa.org> |
| International Detention Coalition (IDC) | <https://idcoalition.org> |
| International Committee of the Red Cross (ICRC) | <https://www.icrc.org/en> |
| International Federation for Human Rights | <https://www.fidh.org/en/about-us/What-is-FIDH/> |
| UN Human Rights Office of the High Commissioner (OHCHR) | <https://www.ohchr.org/EN/pages/home.aspx> |
| World Organisation Against Torture (OMCT) | <https://www.omct.org> |
| Penal Reform International (PRI) | [https://www.penalreform.org](https://www.penalreform.org/)  <https://www.penalreform.org/covid-19/resources-related-to-covid-19/> |
| Prison Insider | <https://www.prison-insider.com/en> |
| Prison Watch International | <https://prisonwatch.org> |
| UN | <https://www.un.org> |
| UNICEF | <https://www.unicef.org> |
| UNITAR | <https://www.unitar.org> |
| UNODC | <https://www.unodc.org> |
| Worldwide Prison Health Research & Engagement Network (WEPHREN) | <https://wephren.tghn.org/covid-19-prisons-and-places-detention/> |
| WHO.int | <https://www.who.int> |
| WHO - Africa | <https://www.afro.who.int> |
| WHO - Americas | <https://www.paho.org/en> |
| WHO - Eastern Mediterranean | <http://www.emro.who.int/index.html> |
| WHO - Europe | <https://www.euro.who.int/en/home> |
| WHO - South-East Asia | <https://www.who.int/southeastasia> |
| WHO - Western Pacific | <https://www.who.int/westernpacific> |
| World Prison Brief | <https://www.prisonstudies.org> |
|  | <https://www.prisonstudies.org/news/international-news-and-guidance-covid-19-and-prisons> |
| **Google site search keywords** | |
| COVID-19, coronavirus, sars-cov | prison, detention, custody |
| **Google site search**conducts a keyword search of the above websites | |
| Example search: site:who.int (("COVID-19" OR "coronavirus" OR "sars-cov") AND ("prison" OR "detention" OR "custody")) | |

Information hubs for manual searching

| Information hub name | Web address |
| --- | --- |
| APT - The COVID-19 and Persons Deprived of Liberty Information Hub and Collaborative Platform | <https://datastudio.google.com/reporting/c686bea7-3152-4dd2-b483-fce072f3ddbf/page/UkoKB> |
| World Prison Brief | <https://www.prisonstudies.org/news/international-news-and-guidance-covid-19-and-prisons> |
| WEPHREN | <https://wephren.tghn.org/covid-19-prisons-and-places-detention/> |
| EuroPris | <https://www.europris.org/covid-19-prevention-measures-in-european-prisons/> |
| ICPA – Covid-19 Information | <https://icpa.org/covid-19-information/covid-news/> |
| Prison Insider | <https://www.prison-insider.com/en/articles/coronavirus-la-fievre-des-prisons> |
| Lachsz et al. (compiled sources) | <https://www.publicdefenders.nsw.gov.au/Documents/lachsz-covid-19-resources.pdf> |
| National Juvenile Justice Network | <https://www.njjn.org/article/resources-compiled-regarding-covid-19-and-vulnerable-populations> |
| NIPH Live Map of Covid-19 evidence | <https://www.fhi.no/en/qk/systematic-reviews-hta/map/> |
| Independent Advisory Panel on Deaths in Custody | <https://www.iapondeathsincustody.org/covid-19> |
| OPCAT – International Covid-19 news | <https://canadaopcatproject.ca/international-covid-19-news-views/> |
| Urban Institute – Resources for Correctional Institutions Regarding COVID-19 | <https://urbanorg.app.box.com/s/yqsqcepd1ryvp9ntekls6ims2fmsjnr7> |
| *National Commission on Correctional Health Care* | <https://www.ncchc.org/> |
| National Institute of Corrections | <https://nicic.gov/coronavirus> |
| Prison Policy Initiative (lobby group advocating for rights of people in prison) | <https://www.prisonpolicy.org/virus/> |
| Danish Institute Against Torture | <https://www.dignity.dk/en/nyheder/reducing-overcrowding-in-pre-trial-detention-and-prison-in-the-context-of-covid19/> |
| Royal Society of Canada | <https://rsc-src.ca/en/voices/canadian-prisons-in-time-covid-19-recommendations-for-pandemic-and-beyond> |
| Children of Prisoners Europe | <https://childrenofprisoners.eu/covid-19-call-to-action-protect-vulnerable-families-and-children-in-alt-care-europe/> |
| Human Rights and Democracy Network | <https://hrdn.eu/covid-19/prisons-and-detention-2/> |
