# Supplementary material for: A rapid review of early guidance to prevent and control COVID-19 in custodial settings
Source: Health Justice. 2021 Oct 15;9:27. doi: 10.1186/s40352-021-00150-w (PMC8518275; doi:10.1186/s40352-021-00150-w)
Supplement: Supplementary file 1 [file 40352_2021_150_MOESM1_ESM.docx]

**Appendix S1** List of recommendations by domain and sub-domain

| Domain/sub-domain |  | Recommendations* |
| --- | --- | --- |
| Domain 1: Planning and preparedness | Facility-level | 1. Comprehensive and regularly updated local risk assessments should form the basis of preparedness and response plans 2. Publicly disclose COVID-19 preparedness and response plans, including to facility staff and people in custody 3. Develop infection prevention and control protocols early and identify designated teams/staff members responsible, timelines, and strategy to ensure fidelity to the protocol 4. Establish outbreak management teams comprised of health and correctional staff, and the local public health service 5. All plans should comply with relevant legislation and infection control standards 6. Security incidents, riots and unrest must be anticipated and incorporated into planning 7. Establish close collaboration between correctional staff and facility health care staff for early detection and action 8. Review and revise existing emergency response, hazard, and disaster plans to account for changes due to COVID-19 9. Ensure adequate stock levels of materials including personal protective equipment (PPE), hand hygiene products, cleaning supplies and disinfectants, diagnostic materials, medications. Identify equipment providers and supply chains 10. Maintain accurate lists of people in custody for monitoring, to safeguard against overcrowding and to accurately identify individuals at higher risk of COVID-19 infection 11. Develop contingency plans to prepare for staff shortages 12. Utilise the World Health Organization’s newly developed preparedness and planning checklist(1) |
|  | Regional | 13. Include prison health and correctional authorities in the overall public health response, rather than planning and operating in isolation 14. Establish close contact with other correctional facilities for disease surveillance and between-facility transfers 15. Establish close contact with hospitals and health services for medical care of confirmed cases or for non-COVID cases if prison health services become overwhelmed during the pandemic 16. Establish close contact with regional health departments to be involved in regional planning and communication 17. Establish good coordination between stakeholders at national and subnational levels involved in risk communication, to allow for rapid clearance of timely, transparent and consistent communication messaging 18. Liaise with political leaders and policymakers to ensure testing is available to correctional systems in their jurisdictions 19. Liaise with Federal, State, and Territory Governments for information sharing, surveillance, and local epidemiology 20. Consider transitioning the governance of facility health services to a ministry of health rather than ministry of justice |
| Domain 2: Creating safer physical environments | Personal and hand hygiene | 21. Consider relaxing restrictions on allowing alcohol-based hand sanitiser (at least 60% alcohol) in custodial settings where security concerns allow, or provide disinfectant wipes if the provision of alcohol-based sanitiser is not possible 22. Provide no-cost access to soap, regularly changed personal towels, hot water, and tissues to support personal hygiene 23. Continuously restock hygiene supplies throughout the facility 24. Train people in custody and staff on how to maintain adequate hygiene (including good cough etiquette, hand washing protocols(2)) and ways of COVID-19 transmission, in a language and medium they understand 25. Consider allowing staff to carry individual-sized bottles of alcohol-based hand sanitiser 26. Establish mandatory hand washing or sanitising stations at entrances and throughout the facility |
|  | Cleaning and sanitation | 27. Implement intensified cleaning and disinfection procedures, increasing frequency to several times per day in communal areas (e.g. showers, lavatories, canteens) and high touch surfaces (e.g. phones, switches, door handles) 28. Increase frequency of laundry and waste management 29. Increase the number of staff or people in custody trained to clean and disinfect common areas 30. Use EPA-registered disinfectants effective against the virus that causes COVID-19(3) 31. Ensure adequate oversight and supervision of all individuals responsible for cleaning and disinfecting 32. Ensure adequate stocks of cleaning supplies 33. Safely and securely store all bleach or chlorine-based cleaning products 34. Follow established standards and protocols for cleaning and disinfection(3, 4) |
|  | Physical distancing | 35. Implement strict physical distancing rules to maintain a 1.5-2 meter distance between individuals at all times 36. Limit the number of operational entrances and exits to the facility 37. Implement staggered timetables for lock-in/lock-up, meals, and recreation to prevent crowding and contact 38. Enforce increased space between individuals in holding cells, waiting areas, and line ups (e.g. at intake and in medical facilities) 39. Adapt recreation by (a) choosing recreation spaces that allow for adequate physical distancing (e.g. 1 person per 4 square meters) and (b) restricting recreation space usage to one housing unit or ‘cohort’ (*see ‘Cohorting’*) 40. Adapt meal time protocols by (a) rearranging seating in canteens to support physical distancing (e.g. remove every other chair or seat on one side of each table only) or (b) distribute meals to individuals in housing units or cells, rather than in common areas 41. Adapt group activities by (a) limiting their size and frequency, (b) increasing space between individuals, (c) suspend group activities when physical distancing is not possible, (d) move group activities to outdoor venues where possible 42. Adapt housing by (a) reassigning sleeping spaces to provide adequate space between individuals, and thoroughly cleaning them prior to reassignment; (b) arrange bunks for head-to-foot sleeping; (c) staggering scheduled movements of housing units throughout the day to prevent population mixing; (d) provide single accommodations with solid walls wherever possible, especially for people in custody displaying COVID-19 symptoms or are otherwise medically vulnerable 43. Designate a room near each housing unit to evaluate individuals with COVID-19 symptoms, rather than having them walk through the facility to be evaluated in the medical unit 44. Ensure at the very minimum one hour of ‘out-of-cell’ time per day, as per the United Nations Standard Minimum Rules for the Treatment of Prisoners(5) |
|  | Cohorting | 45. *Cohorting* is defined as the practice of isolating multiple laboratory-confirmed COVID-19 together as a group(3, 6); this principle can be applied to group and separate high risk people or other groups of people in custody to reduce the risk of transmission 46. Apply cohorting to separate vulnerable people who are at higher risk of COVID-19 such as people with existing comorbidities (e.g. chronic respiratory disease, diabetes, HIV) and/or compromised immune systems, and the elderly 47. Apply cohorting to separate new admissions from the rest of the population of people in custody for 6-14 days(7, 8) or 14 days(3) 48. People in custody should remain within cohorted groups for all daily activities (e.g. meal times, recreation), and cohorts should maintain absolute physical distancing from other cohorts 49. Staff designated to specific cohorts should not move across cohorted units especially during an outbreak 50. Do not cohort confirmed COVID-19 cases with suspected COVID-19 cases, and do not cohort suspected COVID-19 cases together Cohorting of suspected, confirmed, or close contacts of COVID-19 cases should occur only if individual medical isolation of suspected or confirmed COVID-19 cases is absolutely not possible 51. Any cohorting arrangements must observe the same separation and protective rules regularly in place to protect the physical integrity of people at higher risk of suffering violence and discrimination from other people in custody 52. In immigration detention settings, cohorting must preserve family units to the fullest extent possible |
|  | Day-to-day PPE | 53. Refer to guidance on recommended PPE for people in custody and staff in custodial settings(3) 54. Ensure that people in custody and staff are properly trained to correctly store, don, doff, and dispose of PPE 55. Ensure that PPE (including facemasks, eye protection, gowns, and gloves) are available immediately outside of housing units or cells 56. Post signage on the door or wall outside of housing units or cells that clearly describe the type of precautions and PPE required 57. Staff must be property ﬁtted for PPE items such as masks and face shields 58. PPE should be worn for all contact, not just for contact with suspected or confirmed COVID-19 cases 59. Ensure that sufficient stocks of PPE (including facemasks, N95 respirators, eye protection, disposable medical gloves, and disposable gowns/one-piece coveralls) are maintained, and develop contingency plans in the probable event of PPE shortages 60. All used PPE must be disposed of as hazardous clinical waste 61. Cloth masks are not recommended |
|  | Ventilation | 62. Increase the number of air exchanges per hour and supply as much outdoor air as possible to decrease any potential risk of aerosol transmission (recommended to open windows at least twice per day for 30 minutes) 63. Regularly clean and disinfect air inlets of air conditioners or air exchanges 64. In the event a possible, probable, or confirmed case of COVID-19 has been present in a specific indoor space, the space should be well ventilated with fresh air for a minimum of one hour before others can enter |
|  | Non-medical transfers | 65. Restrict all transfers of people in custody to and from other jurisdictions and facilities unless necessary for medical evaluation, medical isolation/quarantine, clinical care, extenuating security concerns, or to prevent overcrowding  66. If a transfer is absolutely necessary, arrange adequate time for COVID-19 testing and results before transfer. At the very minimum, if testing is unavailable, perform verbal screening and a temperature check (guidance provided by the US CDC(3)). 67. If transfer of a suspected, probable, or confirmed case of COVID-19 must occur, ensure the receiving facility has capacity to properly isolate the individual upon arrival 68. Ensure that staff transporting a suspected, probable, or confirmed case of COVID-19 wear adequate PPE and the vehicle is cleaned thoroughly after transport 69. Consider suspending work release and other programs that involve the movement of people in custody in and out of the facility |
| Domain 3: Case identification and screening |  | 70. Increase capacity for immediate, rapid on-site testing and medical diagnosis 71. Where resources allow, install thermal scanners or utilise no-contact thermometers to monitor anyone entering the facility 72. Testing must be freely, equitably, and universally accessible to all people in custody and staff 73. Make regular testing available for all people in custody, regardless of symptoms 74. Conduct verbal screening and temperature checks at intake (including new intakes from other facilities), prior to attending the healthcare unit, and prior to any change in housing arrangements within the facility utilising existing questionnaires(3, 9) 75. Record travel history and possible contact with COVID-infected persons at intake 76. Universal access to COVID-19 testing for all symptomatic and asymptomatic people in custody is strongly recommended where resources allow. If testing is severely limited, it should be prioritised for individuals at high risk of COVID-19, individuals at high risk of transmitting COVID-19 (e.g. cellmates or residents of housing units with at risk individuals, individuals being transferred out of the facility, and healthcare staff), and individuals displaying COVID-19 symptoms (e.g. syndromic surveillance). Note that relying on symptomatic testing will miss asymptomatic and pre-symptomatic cases and result in a greater number of outbreaks at the facility. 77. Provide all staff with appropriate COVID-19 training and resources to ensure testing and diagnosis protocols are maintained 78. Encourage people in custody and staff to self-report if they experience any COVID-19 symptoms, and ensure they are educated on common COVID-19 symptoms and transmission pathways 79. Mitigate violence, stigma, and discrimination against suspected or confirmed cases 80. Perform verbal screening and temperature checks for all staff arriving for shift, on a daily basis 81. Consider quarantining all new intakes for 14 days, where space and resources allow. Recommendations on routine intake quarantine were mixed, with the World Health Organization discouraging its use due to unnecessary and negative impacts on mental health and preferred cost-effectiveness of screening. |
| Domain 4: Case management | Clinical management | 82. Ensure that all suspected or confirmed cases of COVID-19 have immediate access to necessary health care 83. Assess whether the confirmed case of COVID-19 can be medically managed within the facility, otherwise, arrange transfer to hospital, specialist facilities, or other health services 84. Designate a team of health care workers and other staff to care exclusively for suspected or confirmed COVID-19 cases 85. Designate specific housing areas and bathrooms to isolate confirmed COVID-19 cases individually, or in groups if space is limited 86. If a suspected or confirmed case is due to be released and further healthcare is determined to be required by healthcare staff, arrange transfer to a healthcare facility under the jurisdiction of local health authorities. If healthcare is not required, they should ideally be released to continue home isolation and follow up by local medical services as necessary. 87. If a suspected or confirmed case is due to be released and is homeless, continue treatment in place or arrange with temporary accommodation staff to continue isolation and arrange health care if required(10, 11) 88. Include plans for managing COVID-19 related deaths in custody 89. Refer to the World Health Organization’s newly developed checklist for responding to and managing COVID-19 cases(1) |
|  | Medical isolation | 90. Medical isolation must only be applied in cases where it is medically necessary and result in living conditions that are distinct from punitive solitary confinement; solitary confinement should only be used in very exceptional cases, for as short a time as possible, and only as a last resort 91. Isolation has a disproportionate impact on those with mental illness or other disabilities and indigenous peoples, and this needs to be considered when implementing isolation and quarantine measures 92. Provide meals directly to individuals in medical isolation, rather than in common spaces, and directly dispose of food wares in the isolation room; non-disposal food service items should be handled with gloves and washed in hot water 93. Exclude all individuals in medical isolation from any group activities during the period of isolation 94. Ensure that the purpose and conditions of medical isolation and quarantine are clearly described to people in custody to encourage reporting of exposure and symptoms, as well as their advocates and the staff that oversee them 95. Ensure that people in isolation have access to technologies allowing for communication with their family and friends (e.g. telephones, video conferencing) to combat the potentially harmful psychological impact of confinement 96. Ensure that people in medical isolation are granted the right to notify a third party and communicate with legal counsel 97. Ensure that people in medical isolation are provided with additional psychological support if it is needed, both during and after isolation 98. Ensure that people in medical isolation have meaningful human contact every day 99. Ensure that people in medical isolation receive an absolute minimum of one hour of fresh air per day, as per human rights standards(5, 12, 13) 100. Inform family members of people in custody who are placed in medical isolation 101. Implement strategies to release or transfer people to locations that have capacity to meet acceptable safety and ethical standards of quarantine and medical isolation if it is not possible at the current facility 102. Isolate suspected or confirmed cases individually, ideally with their own bathroom, solid walls, and a solid door that closes fully 103. Avoid isolating confirmed COVID-19 cases as a group; if absolutely necessary, isolate as a cohort in a well-ventilated space with solid walls and a solid door that closes fully and implement physical distancing strategies (*see ‘Physical distancing’*) 104. Avoid transfers of confirmed COVID-19 cases to other facilities for medical isolation, unless no other options are available. 105. Consider installing temporary solutions such as large tent blocks away from areas frequented by the general facility population to isolate confirmed COVID-19 cases, if no such space is available in existing buildings 106. Document and communicate all decisions regarding medical isolation with local health officials 107. Terminate medical isolation when the following criteria have been met: (When COVID-19 testing is available) the individual has been free from fever for at least 72 hours AND the individual’s symptoms have improved AND the individual has received two negative COVID-19 test conducted at least 24 hours apart; (Or, where COVID-19 testing is unavailable) the individual has been free from fever for at least 72 hours AND the individual’s symptoms have improved AND at least 7 days have passed since the first symptoms appeared(1, 3, 14) |
|  | PPE | 108. Ensure that all suspected or confirmed COVID-19 cases wear a clean face mask at all times when outside of the medical isolation space, and whenever another individual enters; masks should be changed at least daily or when visibly soiled or wet 109. Ensure staff who are in direct contact with a suspected or confirmed COVID-19 case are protected by at least the minimum level of PPE: medical mask, full gown, gloves, eye protection (goggles or face shield), clinical waste bag, hand hygiene supplies, disinfectants 110. Handle all laundry and food items of a suspected or confirmed COVID-19 case with full PPE 111. Ensure that all relevant staff are trained and deemed competent in the proper use of PPE, including donning and doffing procedures. 112. Provide refresher training for all existing health staff including non-clinical support staff and new staff 113. Ensure all PPE is changed every 2-4 hours or after each interaction with a suspected or confirmed case 114. Ensure that PPE is immediately discarded into hazardous waste bins |
|  | Medical referral and transfer | 115. Notify correctional facilities or health care services prior to external transfer of a suspected or confirmed COVID-19 case 116. Thoroughly clean and disinfect vehicles used to transfer a suspected or confirmed case of COVID-19 before it is used again 117. Dedicate specific vehicles for transfer of suspected or confirmed COVID-19 cases 118. Thoroughly clean and disinfect the room where a suspected or confirmed COVID-19 case was housed prior to transfer before it is occupied by another individual 119. Inform receiving facilities of the individual’s COVID-19 status to ensure that they are prepared to provide proper isolation |
|  | Contact tracing | 120. Develop in-facility contact tracing teams (CTTs) to quickly respond to suspected or confirmed cases of COVID-19 that occur. CTTs should include health care staff with the assistance or guidance of local public health authorities. 121. Close contacts should be defined as any individual that has been within close proximity of a suspected or confirmed COVID-19 case for a prolonged period of time, or has had direct contact with infectious secretions of a COVID-19 case 122. Contact tracing should begin immediately after a suspected COVID-19 case is identified in the facility to avoid delays in reducing transmission, not after a positive test is confirmed 123. If an individual is determined to have had close contact with a suspected or confirmed case, place the individual under quarantine for 14 days and monitor for symptoms twice per day(3) 124. If an individual is quarantined due to contact with a suspected case who is subsequently tested for COVID-19 and receives a negative result, the quarantined individual should be released from quarantine restrictions 125. Develop an observation registry for recording prisoners demonstrating symptoms of COVID-19 and identifying prisoners who were in close contact to that prisoner. Update at least twice a day (e.g. morning and evening). |
| Domain 5: Communicating to people in custody, staff, and families |  | 126. Provide regular, prompt, accurate, transparent, accessible, and up-to-date information on COVID-19 risk reduction, signs and symptoms, health risks, and testing/diagnosis to prevent fears, rumours, and misperceptions from circulating among persons in custody, impede people from seeking medical care 127. Provide timely, transparent, and consistent information on the COVID-19 situation in the facility and local community 128. Provide comprehensive information to people in custody and their loved ones on new preventive measures implemented in the facility, the reasons behind them, their time frame, their impact on daily routine, and alternative mechanisms or compensatory measures in place to offset their impact (e.g. increased access to free telephone/video calls in place of in-person visitation) 129. Provide specific communication to staff and people in custody regarding procedures for medical isolation 130. Openly communicate departures from initial COVID-19 preparedness and response plans to staff, people in custody, and the public 131. All communications to persons in custody, families, and staff must be accessible to people with cognitive, intellectual, or physical disability and consider their diverse linguistic needs, and health literacy levels, and native languages. 132. Use simple and consistent messaging, utilising imagery wherever possible 133. Utilise a variety of communication strategies, including signage, movies, and information sessions 134. Provide ample opportunities to people in custody and staff to ask any questions on COVID-19 135. Engage people in custody in the development or assessment of communication strategies and develop multidirectional communication pathways – people in custody will identify areas for improvement and can also provide information about unrest 136. Tend towards sharing more versus less information to people in custody and their families to maximise transparency, and maintain and establish trust 137. Utilise the World Health Organization’s newly developed communication tools and briefing notes for people in custody and staff(1) |
| Domain 6: External access and visitation | In-person access & visitation | 138. Restrict all non-essential visitors from entering the facility, including: vendors, volunteers, tours, hairdressers, clergy, allied health professionals, and intimate partners, family, and friends of people in custody 139. Conduct verbal screening and temperature checks for all visitors (*see ‘Case identification and screening’*) 140. Provide essential visitors with information prior to in-person visitation regarding screening and infection control protocols in place 141. Instruct essential visitors to postpone their visit if they have even mild flu-like symptoms 142. Display signage at facility entry points clearly explaining screening processes and mandatory infection control measures in place 143. Provide alcohol-based hand sanitizer with at least 60% alcohol in all visitor entrances, exits, and waiting areas  144. Perform contactless temperature checks and verbal screening for COVID-19 symptoms, close contact with cases, and travel history for all visitors at entry; visitors who decline screening or do not pass screening must not be allowed to enter the facility 145. Ensure appropriate physical distancing and infection control measures during essential in-person visits, including: hand hygiene protocols, PPE including fitted mask and gloves, and physical separation via a glass/plastic screen or wall 146. Consider restricting in-person visits from individuals over 60 years old, individuals who suffer from chronic illnesses, pregnant women, and minors based on the current facility COVID-19 situation and recognising the potential higher risk of infection in custodial settings 147. Ensure that there are ways for families and loved ones to deliver food and other essential supplies (e.g. food and drinks, clothing, medication, money, sanitary items) to people in custody and that they are safely accepted, disinfected, and distributed following best practice protocols to minimise the risk of transmission 148. Ensure that restrictions on in-person visitation are not extended for longer than epidemiologically necessary 149. Utilise the World Health Organization’s newly developed COVID-19 visitor brief(1) |
|  | Remote access & visitation | 150. Ensure that any visitor restrictions implemented do not affect the availability of legal counsel, physical and mental health services, or other essential services 151. Ensure that any restrictions on contact with the outside world are offset by providing increased frequency of access to alternative forms of communication, including telephone, video calls, and e-mail 152. Increase the standard permitted length of phone calls for people in custody 153. Ensure that all access to telephones, video calls, e-mail, and other alternative forms of communication are provided free of charge during the time period that any restrictions are imposed on regular in-person visitation 154. Recognise the essential role that contact with loved ones plays in the mental and emotional wellbeing of people in custody, particularly during a public health crisis |
| Domain 7: Psychological and emotional support | People in custody | 155. Ensure continued care and pharmacotherapy for people in custody with existing mental health diagnoses 156. Provide additional psychological support services to all people in custody during periods of additional restrictions on freedom, especially if in medical isolation 157. Provide additional bereavement support and counselling to account for increase in loss of loved ones due to COVID-19 158. Brief psychological intervention and/or psychoeducation should be offered and undertaken to address increased anxiety faced by those worried about COVID-19 both in respect to themselves and their family 159. Consider delaying planned changes to or withdrawal from medicines since anxiety, depressive, and psychotic symptoms are likely to worsen due to stress and social disruption; patients may be at increased risk of relapse or recurrence of affective and psychotic illness 160. People in custody should be given timely, transparent, consistent, informative, proactive, and meaningful information about how to stay safe and healthy during imprisonment 161. Train healthcare workers in psychological first aid 162. Consider the use of online counselling tools and web platforms as an additional support for people in custody, without replacing professional support where it is required |
|  | Staff | 163. Monitor stress, burnout, and fatigue among staff and normalise discussions about difficulties faced in their roles 164. Provide additional, no-cost access to psychological support and counselling programs for all staff in need and ensure it is clear how to access them 165. Psychological and therapeutic staff on site should provide informal opportunities for debrief and discussion 166. Create informal opportunities for staff debriefing with colleagues to build resilience and maintain social connection |
|  | Families | 167.  Contact family members of children in detention to educate them about their rights and to facilitate remote or in-person visitation 168. Support children with imprisoned parents, who may face heightened anxiety about the well-being of their parents and experience traumatic loss due to restrictions on visitation during the pandemic |
| Domain 8: Adapting healthcare provision |  | 169. Ensure that existing health care services continue to be provided as usual for non-COVID-19 patients and health concerns, particularly in regard to uninterrupted access to care for people with specific needs (e.g. mental health, disability, infectious disease, substance use, other chronic health conditions, and pregnant women) 170. Review each patient’s pre-existing physical health vulnerability to support clinical management and patient-level decisions 171. Coordinate facility-level healthcare and psychiatry with general medical and psychiatric care authorities in the community 172. Use telehealth/telemedicine wherever appropriate and where resources allow 173. Offer the seasonal influenza vaccinations free of charge to all persons in custody including new intakes to speed the detection of COVID-19 and reduce pressure on healthcare resources 174. Consider increasing keep-on-person medication orders to cover 30 days in case of healthcare staff shortages 175. Improve on-entry health screening measures to ascertain level of risk from COVID-19 infection and support ‘cohorting’ (*see ‘Creating safer physical environments’*) 176. Facilities without onsite healthcare capacity should plan for how they will ensure that suspected COVID-19 cases will be isolated, evaluated, tested (if indicated), and provided necessary medical care 177. Suspend or eliminate co-payment and other fees for health services; at a minimum, eliminate co-payment for respiratory symptoms 178. Implement standard disinfection, cleaning, and infection control measures for medical transfers outside of the facility 179. Form strong support networks of mental health clinicians to support the mental health needs of people in custody 180. If in-person visitation is restricted, replace direct access to allied health professionals (e.g. occupational therapists, physiotherapists, social workers) with remote access where possible, and encourage the development of individual activity or health plans that can be completed independently by the patient |
| Domain 9: Adapting recreation, programming, and services |  | 181. Ensure access to fresh air does not fall below an absolute minimum of one hour per day, as per human rights standards(5, 12, 13) 182. Consider suspending all congregate activities for at least 4 weeks pending confirmation of COVID-19 in the facility 183. Completely suspend group and vocational activities if they are determined to pose perceivable risk, including those that require visitors, and identify alternative forms of activity to support the mental health of incarcerated/detained persons 184. Adapt essential group activities or group activities deemed to be an acceptable level of risk by: limiting the size of group activities, moving them to outdoor areas or larger spaces that permit physical distancing and adequate ventilation, limiting participation to only within housing units or established cohorts (*see ‘Cohorting’*) 185. Adapt essential vocational activities or vocational activities that are deemed to be an acceptable level of risk by: prohibiting any individuals with flu-like symptoms to attend, restricting the number of individuals in the work space, and regularly disinfecting equipment and work spaces before and after each shift 186. Consider suspending all work release programs that involve movement of people in custody in and out of the facility 187. Adapt vocational programs to support pandemic relief and preparedness, where possible and when it can be done safely, such as shifting towards cleaning and disinfection of the facility or the production of PPE 188. Ensure that people in custody receive continued compensation if vocational activities are suspended for reasons due to the pandemic 189. Replace suspended leisure activities (e.g. recreational or sport activities) by providing additional privileges to people in custody, such as additional TV or entertainment options, additional out-of-cell activities that allow for social distancing 190. Sub-divide recreation spaces or stagger access to recreation space by housing units or cohorts rather than restricting access entirely 191. Replace in-person education with remote learning, especially where exams are scheduled 192. Replace all in-person religious services and social activities with access to virtual religious services and networks within the facility, social media, and peer-to-peer support networks 193. Consider implementing new programming that can be conducted remotely and supports the emotional and mental wellbeing of people in custody, such as mindfulness, wellbeing applications, and in-cell exercise programs 194. Ensure that all recreational, cultural, leisure, and vocational spaces have visual information (e.g. posters) relating to respiratory hygiene, social distancing, and general information on COVID-19 symptoms and transmission |
| Domain 10: Adapting legal services and processes | Hearings and proceedings | 195. Ensure timely access to legal counsel and court hearings 196. Use alternative strategies to limit in-person court hearings (e.g. video conferencing) as long as they do not compromise an individual’s fundamental right to a fair trial; individual needs assessments should be completed to assess whether defendants are at heightened risk of vulnerability in a remote trial (e.g. young people, people with disabilities) 197. Implement standard infection prevention and control when transferring people in custody to and from in-person court hearings 198. Prosecutors should carefully consider the health risk of any custodial sentence, and increase the use of non-custodial alternatives to detention (both pre-trial and post-trial) 199. Courts should continue to hold all hearings. Emergency courts should be established to address backlogs of unsentenced people held in custody awaiting sentencing. |
|  | Access to legal representation | 200. Ensure continued, secure, no-cost, and confidential access to legal counsel via telephone or video conferencing 201. All legal representatives attending in-person should be screened for COVID-19 symptoms 202. People in custody should have access to secure spaces for confidential discussions with their legal representative 203. People in custody must be able to exercise the right to challenge the legality of deprivation of liberty during the pandemic 204. Additional in-kind resources may be needed to support Indigenous legal services to meet the increased need during the pandemic |
|  | Bail, remand, probation, parole, and community supervision | 205. Reduce intake into probation and parole to only those who absolutely need and can benefit from community supervision, or those who may benefit from a more limited-contact arrangement 206. Courts and paroling authorities should release people from supervision who are at low risk or have shown they no longer need to be under supervision; persons on probation or parole for more than two years who are in compliance should be considered for discharge 207. Reduce the term of probation and parole to only as long as necessary to achieve the goals of supervision 208. In-person meetings with probation staff should be replaced by remote communication via phone or video conferencing 209. Review all cases of pre-trial detention to identify cases determine whether it is strictly necessary, and extend the use of bail and other alternatives to pre-trial detention for all individuals who do not present high flight risk or risk to the public 210. Lift cash bail systems to ensure pre-trial detention is not excessively used 211. Community supervision staff should tailor their interactions with clients, focusing on identifying and targeting the most significant needs for each person on their caseloads 212. Temporarily suspend detention for technical violations or noncompliance with terms of community supervision (e.g. failing a drug test, missing probation appointments, violating curfew) |
| Domain 11: Decarceration | Reducing system involvement | 213. Reduce the number of people in custody to protect the health of people in custodial settings, staff, and the broader community 214. Limit the use of pretrial detention wherever possible  215. Increase the use of non-custodial alternatives to detention at the pretrial, sentencing, and posttrial stage (e.g. grant bail to people on remand, house arrest, electronic monitoring, probation or judicial supervision, community service, diversion to treatment programmes) 216. Consider inequitable impacts of financial-based non-custodial alternatives to detention (e.g. fines) 217. Raise the age of criminal responsibility  218. Decriminalise COVID-19-related offences and technical offences |
|  | Releasing people in custody | 219. Reduce the number of people in custody so that (a) all people in custody can engage in physical distancing and (b) there is sufficient capacity to put all people with COVID-19, and their close contacts, in non-punitive isolation or quarantine with access to appropriate medical care  220. Review existing cases of detention in all custodial settings to determine whether detention is still justified, necessary, and proportionate in the prevailing context of the COVID-19 pandemic, particularly for those at high risk of complications from COVID-19  221. Develop early release policies and criteria that are grounded in data; fully communicated to staff, people in custody, and the public; and consistently followed. Criteria for determining the eligibility for such emergency release measures should be based on a careful balancing of vulnerability of people in custody against public safety and be accompanied by appropriate safeguards to the safety and the rights of witnesses and victims. 222. People released from custody during the pandemic should not be required to appear in person for check-ins and non-essential court proceedings where infection could spread 223. Develop communications strategies to ensure communities are informed and educated on the reasons for early release in order to support community reintegration and reduce stigma and discrimination against people released from custody 224. Priority populations for release include: people in custody who pose no to very low risk to the public (e.g. those detained for minor or non-violent offenses, those with sentences nearing completion), people serving pretrial detention for non-violent or lesser offenses that are considered to have a low likelihood of reoffending, juveniles, pregnant women and women with dependent children, older persons, and people in custody with underlying medical conditions that increase their risk of complications from COVID-19 infection 225. Authorities must not release people in custody without their consent 226. Early release should never subject the individual to a greater risk of COVID-19, homelessness, destitution, or immediate deportation |
| Domain 12: Release and community re-integration | Pre-release needs assessment | 227. Invest in discharge planning and re-entry transitions to facilitate prison release of people under newly developed early release policies  228. Have programs in place to ensure that expediated release does not lead to rushed discharge and a lack of discharge planning, which may lead to poorer health outcomes during the pandemic, increase the risk of COVID-19 infection and community transmission, and exacerbate existing epidemics of opioid use, HIV, and inequality(15) 229. Identify people in custody at higher risk of homelessness, unemployment, substance abuse, or becoming victims of violence and arrange for appropriate resources, including medications for addiction treatment, at release 230. Screen for symptoms and/or test for COVID-19 prior to release to prevent spread from custodial settings to the community 231. Create transition plans that ensure basic needs and access to services are addressed including: health coverage, food security, access to communication (e.g. mobile phones), health care, medications, and safe housing where they can comply with public health advice and local COVID-19 restrictions |
|  | Post-release support | 232. For individuals who test positive for COVID-19 at release: arrange for medical isolation (minimum 14 days) either in the facility or in the community setting and ensure medical follow-up during the isolation period and assess need for continued medical care 233. Notify and coordinate with local public health authorities if releasing a confirmed case to isolation and medical follow-up in the community 234. Maintain up-to-date referral information and ensure that referring services are equipped to serve justice-involved people 235. Ensure adequate housing for all people in custody upon release, regardless of COVID-19 status 236. Conditions of release should account for specialised support to enable individuals’ safe reintegration into their family and community environments, accounting for existing or potential lockdown measures 237. Arrange for the safe transit from custody to residence to minimize community contact 238. Liaise with probation and parole officers to support the transition from custody to the community and navigation of health and social services in the community during the pandemic 239. Ensure people with substance use disorder and other chronic conditions receive continued access to medications after release 240. Disseminate prevention kits with masks, soap, hand sanitiser, disinfectant wipes, and local public health contacts at the time of release |
| Domain 13: Workforce logistics | Staff briefings and trainings | 241. Ensure that staff are regularly made aware of protocols issued by the facility, government, health department, and other authorities 242. Provide staff with up-to-date information about COVID-19 and facility policies on a regular basis, including: COVID-19 symptoms and transmission, expectations for facility and individual infection prevention (e.g. hand hygiene, cough etiquette, physical distancing), vulnerable populations at high risk of severe illness, appropriate use of PPE (*see ‘Day-to-day PPE’*), protocols for self-identification of symptoms or close contact with a COVID-19 cases, changes to facility cleaning and disinfection and other procedures, and sick leave policies (*see ‘Staffing polices and protocols’*) 243. Ensure staff understand and are trained on facility-wide infection prevention and control protocols and outbreak management plans, and are prepared to take action if required 244. Ensure that relevant staff understand and workshop how the practice of self-control and restraint will be affected by PPE and infection control measures such as physical distancing 245. Educate and train staff on recognising signs of mental distress, burnout, and agitation among people in custody and colleagues 246. Provide staff with clear guidance for pathways to care and support, including those they can access without disclosing their distress to management 247. Remind staff of the special vulnerability of prisoners and obligations to uphold human rights standards(5, 12, 13) 248. Utilise the World Health Organization’s newly developed staff briefing and training checklists for COVID-19(1) |
|  | Staffing policies and protocols | 249. Fulfil the obligations to adequately protect staff against COVID-19 infection and respect their human rights in regard to their wellbeing 250. Review which routine policies and practices needlessly increase contact between staff and people in custody or with surfaces in highly trafficked areas 251. Minimise the movement of staff across facility areas and cohorted people in custody without compromising security and quality of care 252. Minimise the number of staff work across multiple facilities in the region, or units within the facility, wherever possible 253. Encourage relevant staff to work from home whenever possible 254. Consider offering revised duties to staff who are at higher risk of severe illness due to COVID-19 255. Minimise contact between staff by suspending roll-call and using videoconferencing and e-mail for briefings and advisories 256. Develop contingency plans in the event of a COVID-19 outbreak, and plan for a minimum 20-30% staff absentee rate 257. Retain additional contingency staff and plan to secure additional staff if absenteeism due to COVID-19 threatens facility functioning; establish agreements with other agencies or ministries to allocate temporary personnel (including health providers) if required 258. Ensure staff have sufficient paid sick leave and encourage staff to stay home if they or anyone in their household is symptomatic 259. Maintain a daily logbook of staff who enter and leave the facility, and lists of contact information for contact tracing purposes |
|  | Managing staff as confirmed cases and contacts | 260. Require staff with any flu-like symptoms to stay home or leave work immediately if unwell, and follow public health guidance 261. Ensure that staff who test positive for COVID-19 immediately inform management and personal contacts, and only return to work once they have been confirmed as meeting criteria for release from medical isolation by local public health professionals 262. Ensure that staff members who are identified as close contacts of confirmed cases immediately self-isolate and self-monitor for symptoms until, in the case of symptom presentation, a negative COVID-19 test result is received 263. During a COVID-19 outbreak, ensure that staff only attend work if they are asymptomatic and continuously monitor for symptoms 264. Staff without symptoms of COVID-19 but who share a household with symptomatic or conﬁrmed cases of COVID-19 must follow local public health guidance (e.g. to stay home and self-isolate for 14 days) 265. Alert local public health officials about large increases in staff absenteeism, particularly if due to suspected or confirmed COVID-19 |
| Domain 14: Surveillance and information sharing |  | 266. Develop a data collection plan to index all suspected cases of COVID-19 and track people in custody through diagnosis, treatment, medical isolation (when indicated), and release 267. Ensure privacy of medical information is maintained at all times 268. Create a reporting process for staff to report suspected infected people in custody (i.e. those displaying flu-like symptoms) 269. Establish or adopt outbreak definitions for the purposes of public health reporting, such as that developed by the Communicable Diseases Network of Australia(16): two or more cases of acute respiratory infection in people in custody or staff within 72 hours AND at least one case of COVID-19 confirmed by laboratory testing 270. Continue surveillance of, or include to new surveillance processes, consequential health outcomes of COVID-19 such as self-harm 271. Seek consent of all people in custody prior to sharing their private information or medical files to a third party for surveillance purposes 272. Maintain a register of people coming in and out of the facility including people in custody, staff, and visitors (template developed(1)) 273. Develop or adapt existing data collection systems to analyse trends and inform service delivery during the pandemic 274. Communicate actively and regularly with local health departments regarding suspected and confirmed cases of COVID-19 275. Enhance public communications during the pandemic, and allow senior staff or management to address the media on a regular basis providing real-time information, updates, and situation reports 276. Prepare to collect the following data for public health purposes, depending on jurisdictional requirements: total number of people in custody or staff with fever or respiratory symptoms, number of people in custody or staff with influenza-like symptoms who have died, total number of staff working in the facility, total number of people in custody in the facility, whether appropriate specimens have been collected, results of any specimens received and tested 277. Develop regional or national systems of case reporting and data sharing across custodial facilities; a phased approach may be necessary and may include either a voluntary system of reporting or a sentinel system based on a selection of facilities in the region |
| Domain 15: Independent monitoring and inspection |  | 278. The COVID-19 pandemic “must not be used as a justification for objecting to external inspection of prisons and other places of detention by independent international or national bodies whose mandate is to prevent torture and other cruel, inhuman, or degrading treatment or punishment”(17) 279. Ensure independent inspection and monitoring activities include review of and monitoring for compliance with COVID-19 prevention outbreak management plans and protocols(18) 280. Ensure that independent monitoring bodies are provided access to the facility following agreed-upon arrangements for either in-person or remote inspection, abiding by do no harm principles 281. If remote inspection is agreed upon, facilitate remote (e.g. video) access to the facility, regular updates to monitoring teams, access to information from staff and/or people in custody when warranted, and attendance to any serious incidents 282. Independent monitoring should be viewed as an ‘essential service’ even in times of lock-down to ensure the protection of the rights of people in custody, as well as staff, during the pandemic 283. Independent monitoring bodies should seek the advice and expertise of relevant professionals (e.g. infectious disease experts) and public health authorities, and include healthcare specialists in the monitoring team 284. Ensure monitoring teams abide by all PPE and infection control procedures in place at the facility 285. Ensure people in custody receive information on how to contact monitoring bodies and complaint mechanisms in a confidential manner 286. Results from independent monitoring and oversight should be made publicly available |
| Domain 16: Compensatory measures |  | 287. Facilitate, encourage, and increase access to alternative means of communication with families and loved ones to offset the potentially harmful impacts of restrictions on contact with the outside world, especially during medical isolation (*see ‘Remote access & visitation’*) 288. Ensure that all individuals placed in medical isolation have meaningful human contact every day (*see ‘Medical isolation’*) 289. Increase frequency of and limits on the length of phone calls for all people in custody 290. Ensure that all people in custody have adequate remote access to legal representatives, clergy, and other individuals with whom they have the legal right to consult at least as frequently as was allowed prior to COVID-19 291. Consider issuing non-internet enabled cell phones and/or tablets pre-programmed for access to approved phone numbers and/or email addresses for the duration of COVID-19 restrictions 292. Eliminate all costs associated with access to these forms of communication for people in custody 293. Suspend co-payment or other fees for incarcerated/detained persons seeking medical evaluation for respiratory symptoms (*see ‘Adapting healthcare provision’*) 294. Provide additional and no-cost access to psychological support services, bereavement support, and counselling (in-person or online) during periods of restriction, especially during medical isolation (*see ‘Psychological and emotional support’*) 295. Replace suspended leisure activities (e.g. recreational or sport activities) by providing additional privileges to people in custody, such as TV, movies, tablets, other forms of electronic entertainment, and social/peer-support platforms (*see ‘Adapting recreation, programming, and services’*) 296. Consider providing free access to educational programs (in-person if determined safe, or online) during the pandemic 297. Ensure that people in custody receive continued compensation if vocational activities are suspended for reasons due to the pandemic 298. In jurisdictions where food parcels are allowed in prison, increase their quantity/frequency, or provide financial means to ensure people are able to purchase food if COVID-19 restrictions limit individuals’ financial means 299. Consider the extenuating circumstances of additional restrictions on freedom in custodial settings put in place during the COVID-19 pandemic in bail, probation, and parole reviews (e.g. early release, shortening of sentences, compassionate release) |
| Domain 17: Lifting control measures |  | 300. Restrictions should be put in place only for the period of crisis, and must be lifted as soon as conditions allow 301. Closely monitor local COVID-19 developments and adjust restrictions as appropriate with respect to human rights and public health 302. Terminate medical isolation once the individual meets pre-established clinical and/or testing criteria, in consultation with clinical providers and public health officials (*see ‘Medical isolation’*) 303. Consult with relevant public health guidance to declare a COVID-19 outbreak to be over; existing guidance differs in defining this as no new cases occurring within 14 days (maximum incubation period) following the date of isolation of the case(16) *or* no new cases occurring for 28 days (two incubation periods) after the date of diagnosis of the last confirmed case(19) 304. Designate officials who will be authorised to make decisions about escalating or de-escalating response efforts as the local epidemiologic context changes |
| Domain 18: Learning systems and evaluative frameworks |  | 305. Conduct a review of facility responses after a COVID-19 outbreak, and when the pandemic is declared over 306. Conduct formal debriefs following a COVID-19 outbreak with outbreak management teams, facility management, staff, and relevant stakeholders to improve upon future outbreak management and uphold human rights and public health to the fullest extent possible 307. Utilise existing guidance for conducting audits of outbreak management processes against established best practices(20) 308. Seek the insights and experiences of people in custody during the pandemic, staff, and other essential personnel in any evaluations to determine what worked well, what responses should be modified, and key challenges and considerations for pandemic response 309. Develop a regional, national, or international forum for dissemination and sharing of knowledge to inform future preparation and planning on health crises in custodial settings 310. Use lessons learned from the COVID-19 pandemic to advocate for broader policy reform including improved conditions in places of detention, reduced overcrowding, and ensured compliance with international health and human right standards(5, 12, 13) via indiscriminate treatment of people in custody 311. Advocate for the continued application of successful policy and program adaptations in place during the pandemic that support people in custody (e.g. use of remote technologies and virtual access; prison decongestion strategies; low-resource peer support, mental health, educational programming; and basic infection prevention measures such as improved hygiene and sanitation) 312. Engage in the movement to ‘rethink’ of detention and sentence policies to support public health, safety, and human rights, and to fully realise the vision of the Optional Protocol to the Convention against Torture and other Cruel, Inhuman or Degrading Treatment or Punishment (OPCAT)(21) with regard to best practice in oversight and transparency in places of detention 313. Seek new levels of engagement with prison services or detention authorities to address areas of reform and improve the ability to respond to future public health crises 314. Engage with police, other law enforcement institutions, and the judiciary about risks and opportunities related to pre-trial detention 315. Engage with immigration, law enforcement, border, and other relevant agencies or officials to reduce the use of immigration detention, establish sound alternatives to immigration detention, and to end the immigration detention of children, families, and other migrants such that immigration detention is an exceptional measure of last resort that is legal, necessary, and proportionate to risk 316. Support data-driven evaluation of COVID-19 response in custodial settings by investing in and committing to the systemic collection and dissemination of population counts, population characteristics, COVID-19 testing results (including for staff and contractors), infection rates, death data, infection prevention and control measure applied, contingency and outbreak management plans, and incidents of harm and disturbances, disaggregated by gender, disability status, age, and ethnicity where possible and appropriate 317. Working with Federal, State, and Territory governments for the regular distribution of this information to the public 318. Conduct policy analysis to examine (a) new legal powers relating to detention introduced as part of the response, and their impact on treatment and conditions and (b) impact of policy responses (e.g. early or compassionate release of people in custody) 319. Continue to support independent monitoring activities after the pandemic response 320. Refer to existing guidelines for monitoring during the COVID-19 pandemic(22, 23) 321. Include consultation with people in custody in evaluation activities 322. Robust, ethical research in custodial settings is required to compare approaches and interventions and develop an evidence base for COVID-19 infection(24, 25) |
| Domain 19: Key populations and settings^†^ | Youth detention | 323. Young people should only be detained as a last resort; alternatives to detention and release from custody should be prioritised 324. Immediately release children who can safely return to their families and communities 325. Engage with child protection authorities and other services in reintegration planning to ensure needs are met post-release 326. Remote legal hearings are generally not recommended for young people. This needs to be considered on a case-by-case basis with the full consent of young people and defence attorneys.(26) 327. Support safe in-person visitation for youth remaining in detention; the majority of functions carried out by appropriate adults cannot be performed remotely 328. Ensure uninterrupted access to developmentally appropriate education, leisure, and recreational activities 329. Provide age-appropriate mental health and psychological support for those experiencing COVID-19 related fear and distress 330. Provide additional resources and funding to expand community-based services and supports for youth released from custody 331. Engage young people to meaningfully contribute to and refine COVID-19 response measures 332. Raise the age of criminal responsibility(27) |
|  | Immigration detention | 333. Authorities should stop arbitrary detention and deportation of migrants and asylum seekers, seek alternatives to detention for people currently in immigration detention, and opt for release where possible to reduce incarcerated populations to the lowest possible level 334. Prioritise for early release medically vulnerable people in immigration detention and those who are being held with no prospect for imminent, safe, and legal deportation 335. Enact safe and multifaceted processes to secure legal status and provide case resolution. This could be achieved by extending deadlines; granting bridging visas or issuance of stay permits to allow people to live in the community; expediting the processing of detained individuals’ existing visa applications; and providing alternate methods for filing, reporting and hearings. 336. Ensure people released from immigration detention have appropriate access to income support, social services, and health care in the community 337. Ensure people released form immigration detention have continued protection from immigration authorities after release (e.g. when accessing health or social services) 338. Provide multilingual information and support for migrants in detention and in the community |
|  | Forensic psychiatric | 339. Reconsider involuntary placement of psychiatry patients during the pandemic 340. Implement more robust testing, cohorting, and isolation measures because early release of patients from forensic mental health settings may not be feasible 341. Consider release with appropriate support and management planning for patients who are able to safely return to the community 342. Consider the disproportionate impact of medical isolation on people with mental illness during case management (*see ‘Medical isolation’*) |
|  | LMIC | 343. Address severe overcrowding in low-middle income country (LMIC) prisons by reducing prison populations and prioritising release for people held in pre-trial detention and those at high risk of complications from COVID-19, to address severe overcrowding in LMIC prisons 344. Consider implementing a syndromic approach to case identification and management when testing capacity is severely limited and universal and regular testing is not possible, noting that relying on symptomatic testing will miss asymptomatic and pre-symptomatic cases and result in a greater number of outbreaks at the facility(28) 345. Enact measures for people in custody to safely received food and other supplies from their families 346. Strengthen access to legal support and independent monitoring and oversight |
|  | Women | 347. The United Nations Rules for the Treatment of Women Prisoners and Non-custodial Measures for Women Offenders (the ‘Bangkok Rules’)(13) must be considered in all planning and a gender-responsive approach applied to all facets of the COVID-19 response in custodial settings 348. Provide separate facilities for women and ensure that staff are of the same sex 349. Apply a gender-responsive approach when planning for early release and reintegration into safe environments that considers the gendered impact of COVID-19 and resulting economic crises 350. Consider the risk of gender-based violence, which may be exacerbated by community-based COVID-19 restrictions, when planning for release 351. Prioritise pregnant women and women with dependent children for early release |
|  | Elderly | 352. Due to their vulnerability to COVID-19 infection and low rates of recidivism, older people are a high priority group for early or compassionate release from custody 353. Provide additional support for older people post-release, as it is likely that most will have spent many years in custody and will have experienced destabilisation of their support networks 354. Consider cohorting older and other medically vulnerable people in custody to reduce risk of COVID-19 exposure, following best practice principles (*see ‘Creating safer physical environments’)* |
|  | Indigenous peoples | 355. Avoid over-policing of Indigenous communities and prioritise non-custodial options 356. Prioritise Indigenous peoples for early release and provide robust, comprehensive, culturally safe support following release 357. Include Indigenous-led organisations and community leaders in decision-making via transparent and robust consultation processes 358. Strengthen arrangements for family and cultural contact 359. Ensure Indigenous people in custody are prioritised for testing and treatment and have access to culturally appropriate care 360. Ensure all changes to policies and protocols are communicated in relevant languages and with engagement from relevant community leaders and organisations |
|  | People with a disability | 361. All response measures must align with the obligations stipulated under the Convention on the Rights of Persons with Disabilities(29) 362. Develop individualised plans to ensure continuous access to appropriate care and support, both in custody and following release 363. Be aware of the disproportionate impact of medical isolation on people with disabilities during case management (*see ‘Medical isolation’*) |
|  | People who use AOD | 364. Ensure people who use alcohol or other drugs (AOD) continue to receive harm reduction services in custody and following release 365. Prioritise intravenous drug users and those convicted of drug-related offences for release, ensuring continuity of pharmacotherapy and other supports 366. Ensure people released from custody with opioid dependence have access to harm reduction and overdose prevention services, as COVID-19 may exacerbate risk of overdose and the risk of overdose is high immediately following release from custody(15) 367. Abstinence should be prohibited as a condition of release 368. Loosen drug regulations to reduce populations in custody |
|  | People with mental illness | 369. Anticipate increased mental health diagnoses and exacerbation of existing conditions due to fear, stress, and uncertainty due to COVID-19 370. Preserve continuity of psychological and psychiatric care by implementing online and telephone support services and planning for disruptions to the clinical workforce 371. Develop clear guidance for remote or in-person visitation by psychological and psychiatric care providers 372. Consider the disproportionate impact of medical isolation on people with mental illness during case management (*see ‘Medical isolation’*) |
|  | Other | 373. COVID-19 emergency measures must not be used to silence dissent or criticism or as justification for arbitrary detention 374. Prioritise political prisoners and other prisoners of conscience for early release |
| *Abbreviations: PPE: personal protective equipment; CTT: contact tracing teams; LMIC: low-middle income country; AOD: alcohol and other drugs* *References to source publications are not provided in this table with exception of specific references to checklists or guidelines. Refer to Appendix S2 ‘Summary of included publications’ for classification of publications according to region, type of author, target audience, and target setting. ^†^Key populations were identified in the data when a specific recommendation was made for a population subgroup, and therefore do not represent all populations of people in custody. |  |  |

**References Appendix S1**

1.	Justice and Corrections Service. COVID-19 preparedness and response information package. United Nations Department of Peace Operations; 2020.

2.	World Health Organisation. Guidelines on Hand Hygiene in Health Care. 2009.

3.	US Centres for Disease Control and Prevention. Interim Guidance on Management of Coronavirus Disease 2019 (COVID-19) in Correctional and Detention Facilities. 2020.

4.	Pan American Health Organization. Recommendations for the Cleaning and Disinfection of Sites Where People are Closely Confined and Deprived of Their Liberty: Penitentiaries, Prisons, and Migrant Detention Centers. 2020.

5.	United Nations Office on Drugs and Crime (UNODC). The United Nations Standard Minimum Rules for the Treatment of Prisoners (the Nelson Mandela Rules). 2015.

6.	Public Health England. COVID-19: Prisons and other prescribed places of detention guidance: Government of the United Kingdom; 2020. Available from: [https://www.gov.uk/government/publications/covid-19-prisons-and-other-prescribed-places-of-detention-guidance/covid-19-prisons-and-other-prescribed-places-of-detention-guidance](https://www.gov.uk/government/publications/covid-19-prisons-and-other-prescribed-places-of-detention-guidance/covid-19-prisons-and-other-prescribed-places-of-detention-guidance). 

7.	Commonwealth Human Rights Initiative. COVID-19 and prisons in the commonwealth: Ensuring an effective response. 2020.

8.	European Centre for Disease Prevention and Control. Infection prevention and control and surveillance for coronavirus disease (COVID-19) in prisons in EU/EEA countries and the UK. 2020 3 July 2020.

9.	General Directorate “Execution of Sentences” Bulgaria. Protocols for action in case of COVID-19 in places of deprivation of liberty 2020.

10.	Emory Center for the Health of Incarcerated Persons. Provisional Guidance on Management of COVID-19 in Jails, Prisons and Other Detention Settings. 2020.

11.	The International Legal Foundation. Coronavirus Pandemic: Guidance for Legal Aid Providers to Protect Health and Human Rights of Detainees. 2020.

12.	United Nations Human Rights Office of the High Commissioner. United Nations Standard Minimum Rules for the Administration of Juvenile Justice ("The Beijing Rules") 1985.

13.	United Nations Office on Drugs and Crime. United Nations Rules for the Treatment of Women Prisoners and Non-custodial Measures for Women Offenders (the Bangkok Rules). 2011.

14.	United Nations Institute for Training and Research (UNITAR). Operational toolbox: COVID-19 preparedness and response in places of detention.; 2020.

15.	Mukherjee TI, El-Bassel N. The perfect storm: COVID-19, mass incarceration and the opioid epidemic. International Journal of Drug Policy. 2020:102819.

16.	Communicable Diseases Network Australia. National Guidelines for the Prevention, Control and Public Health Management of COVID‐19 Outbreaks in Correctional and Detention Facilities in Australia. 2020.

17.	World Health Organization. Preparedness, prevention and control of COVID-19 in prisons and other places of detention: Interim Guidance. 2020 15 March 2020.

18.	Manthorpe M. Statement by the Commonwealth Ombudsman Michael Manthorpe on the management of COVID-19 risks in immigration detention facilities. Australia: Commonwealth Ombudsman; 2020 3 June 2020.

19.	Government of Canada Office of the Correctional Officer. COVID-19 Status Update. 2020 23 April 2020.

20.	Dalton CB, Merritt TD, Durrheim DN, Munnoch SA, Kirk MD. A structured framework for improving outbreak investigation audits. BMC Public Health. 2009;9(1):472.

21.	United Nations Human Rights Office of the High Commissioner. Optional Protocol to the Convention against Torture and other Cruel, Inhuman or Degrading Treatment or Punishment (OPCAT). 2002.

22.	Inspectorate of Prisons for Scotland. Alternative approach to scrutiny during the COVID-19 pandemic. 2020 20 April 2020.

23.	New Zealand Office of the Ombudsman. Report on inspections of mental health facilities under the Crimes of Torture Act 1989. 2020.

24.	Montoya-Barthelemy AG, Lee CD, Cundiff DR, Smith EB. COVID-19 and the Correctional Environment: The American Prison as a Focal Point for Public Health. Am J Prev Med. 2020;58(6):888-91.

25.	Nature. Tackle coronavirus in vulnerable communities. Nature. 2020;581(7808):239-40.

26.	National Juvenile Defender Center. Guidance to juvenile courts on conducting remote hearings during the COVID-19 pandemic 2020. 27 March 2020.

27.	Lachsz A, Hurley M. OPCAT, places of detention, and covid-19: Joint submission to the Select Committee on covid-19. 2020 27 May 2020.

28.	The Kirby Institute. Report on COVID-19 and the impact on New South Wales prisoners. 2020.

29.	United Nations. Convention on the Rights of Persons with Disabilities and Optional Protocol.
